# Beaconet: A Reference‐Free Method for Integrating Multiple Batches of Single‐Cell Transcriptomic Data in Original Molecular Space

**DOI:** 10.1002/advs.202306770

**Published:** 2024-05-06

**Authors:** Han Xu, Yusen Ye, Ran Duan, Yong Gao, Yuxuan Hu, Lin Gao

**Affiliations:** ^1^ School of Computer Science and Technology Xidian University Xi'an 710126 China; ^2^ School of Electrical and Information Engineering Beijing University of Civil Engineering and Architecture Beijing 102616 China; ^3^ Department of Computer Science The University of British Columbia Okanagan Kelowna British Columbia V1V 1V7 Canada

**Keywords:** batch effects, large‐scale, molecular feature space, reference‐free, single‐cell datasets

## Abstract

Integrating multiple single‐cell datasets is essential for the comprehensive understanding of cell heterogeneity. Batch effect is the undesired systematic variations among technologies or experimental laboratories that distort biological signals and hinder the integration of single‐cell datasets. However, existing methods typically rely on a selected dataset as a reference, leading to inconsistent integration performance using different references, or embed cells into uninterpretable low‐dimensional feature space. To overcome these limitations, a reference‐free method, Beaconet, for integrating multiple single‐cell transcriptomic datasets in original molecular space by aligning the global distribution of each batch using an adversarial correction network is presented. Through extensive comparisons with 13 state‐of‐the‐art methods, it is demonstrated that Beaconet can effectively remove batch effect while preserving biological variations and is superior to existing unsupervised methods using all possible references in overall performance. Furthermore, Beaconet performs integration in the original molecular feature space, enabling the characterization of cell types and downstream differential expression analysis directly using integrated data with gene‐expression features. Additionally, when applying to large‐scale atlas data integration, Beaconet shows notable advantages in both time‐ and space‐efficiencies. In summary, Beaconet serves as an effective and efficient batch effect removal tool that can facilitate the integration of single‐cell datasets in a reference‐free and molecular feature‐preserved mode.

## Graphical abstract

The batch effect among scRNA‐seq datasets is commonly corrected based on reference and in embedding space. An algorithm is presented that enables removing batch effect in a reference‐free manner and preserving molecular feature space. By extensive comparison with 13 methods, its superior performance in terms of both effectiveness and efficiency is demonstrated. Datasets with more than 10 000 cells can be integrated in 5 min. Additionally, the gene expression space is preserved.

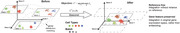

## 1 Introduction

With the advancement of high‐throughput single‐cell sequencing technologies and the decreasing cost of experiments, large‐scale projects such as Human Cell Atlas,^[^ ^1^ ^]^ Mouse Cell Atlas,^[^ ^2^ ^]^ Tabula Muris,^[^ ^3^ ^]^ and Accelerating Medicines Partnership^[^ ^4^, ^5^, ^6^ ^]^ have successfully obtained transcriptional profiling for hundreds of thousands of cells. These projects provided valuable insights into complex biology systems at single‐cell resolution and greatly enhanced the understanding of cell heterogeneity.^[^ ^7^, ^8^ ^]^ However, single‐cell transcriptomic datasets were often generated in multiple experimental laboratories,^[^ ^9^ ^]^ used different sequencing protocols,^[^ ^10^ ^]^ and comprised numerous samples.^[^ ^11^ ^]^ Consequently, batch effect arose among the single‐cell transcriptomic datasets, which distorted the genuine biological signals and potentially led to misleading conclusions.^[^ ^8^, ^9^, ^12^, ^13^ ^]^ The Batch effect referred to the undesired systematic variations in single‐cell datasets that resulted from handling the cells in distinct batches.^[^ ^9^, ^14^ ^]^ These variations can stem from different sequencing lanes, plates or flow cells, laboratories, samples, protocols, and reagent lots.^[^ ^14^ ^]^ As the size and number of single‐cell datasets increase rapidly,^[^ ^15^ ^]^ eliminating batch effects has become a crucial challenge for the integrative analysis of single‐cell transcriptomic datasets.^[^ ^9^, ^14^, ^16^ ^]^

In recent years, several computational methods have been developed to remove batch effects while preserving biological variations in the joint single‐cell datasets of multiple batches. Mutual nearest neighbors (MNN) based methods such as Seurat^[^ ^12^ ^]^ and MNNCorrect^[^ ^17^ ^]^ characterized the cells belonging to the same cell state or type in different batches by matching MNN pairs. However, their performance was affected by the merge ordering of batches when integrating multiple datasets because the correction of batch effect was dominated by the accuracy of a few MNN pairs and the detection of MNN pairs can only be applied to two batches at once, in which the cell population composition is not identical in practice.^[^ ^17^ ^]^ To improve the accuracy of the matching of MNNs, iMAP^[^ ^18^ ^]^ introduced random‐walk mutual nearest neighbors (rwMNN) pairs in deep‐embedding space and corrected batch effect by a Wasserstein Adversarial Network^[^ ^19^ ^]^ (WGAN) based on the matched cell pairs, while FastMNN^[^ ^17^ ^]^ identified the MNN pairs in the principle component (PC) embedding space. Besides, two supervised MNN methods SMNN^[^ ^20^ ^]^ and iSMNN^[^ ^21^ ^]^ improved the effectiveness of MNN pairs by extra cell type labels but their application scenarios were limited. Integration methods such as RPCI^[^ ^22^ ^]^ and Harmony^[^ ^23^ ^]^ tackle the dependency of merge ordering by selecting individual batches as references. Scanorama^[^ ^24^ ^]^ generalized the idea of MNN to multiple batches with the inspiration of panorama stitching algorithms in the field of computational vision and performed well on complex integration tasks.^[^ ^14^ ^]^ DESC^[^ ^25^ ^]^ was an embedding‐based method that needed no information about the batch labels but required the batch effect to be smaller than the biological variations. There were also integration methods with extra goals besides removing batch effects, such as scDML^[^ ^26^ ^]^ and Portal.^[^ ^27^ ^]^ scDML endeavored to preserve the subgroup structure in embedding space when removing batch effect, and Portal was developed for atlas‐level large‐scale datasets integration in PC embedding space. However, as the embedding features were difficult to interpret, the application scenario of these methods which changed the original molecular features was usually limited.^[^ ^14^ ^]^ Although there was a recent proposal^[^ ^28^ ^]^ that attempted to bridge the gap between embedding space with molecular features, it was still a promising direction to integrate multiple batches of datasets in the original feature space.^[^ ^26^ ^]^ Among the existing methods, Scanorama did not require pre‐selected reference or merge‐ordering and also performed integration in the original feature space. However, its performance may be influenced by the scarcity of matched cell pairs.^[^ ^18^ ^]^ Taken together, there were mainly three challenges in integrating multiple batches of single‐cell datasets requiring to be solved simultaneously: 1) the dependence on individual reference or merge ordering; 2) the loss of original molecular feature space for integrated data; 3) accurate integration of datasets without extra information.

In this work, we developed Beaconet (Batch Effect Adversarial Correction Network), a reference‐free method for integrating multiple batches of single‐cell transcriptomic data in the original gene‐expression feature space. Beaconet does not require cell type labels for integration or the matching of cell pairs as anchors. Beaconet learns to match the global distributions of multiple batches of datasets simultaneously and removes the batch variations by a global correction function for cells. Besides this, we also proposed a quantitative metric, Positive Merge Divergence (PMD), which was designed for unbiased evaluation of batch effect removal methods on datasets with batch‐specific cell types. We demonstrated the effectiveness of the PMD metric by comparing it with 4 existing popular metrics on 2 integration tasks with public available datasets. Through extensive comparison with existing state‐of‐the‐art methods, we then demonstrated that Beaconet enables accurate integration in a reference‐free manner and is superior to the compared methods using any possible references or merge orderings. Furthermore, the preserved gene‐expression features enable not only the characterization of cell types and subpopulations but also downstream differential expression analysis. Additionally, applying to Tabula Muris, Beaconet exhibits superior time‐ and space‐efficiency in dealing with large‐scale datasets. The Python implementation of Beaconet is freely available on GitHub (https://github.com/GaoLabXDU/Beaconet). All code scripts, preprocessed data, and the results of our experiments are freely accessible on Figshare (https://doi.org/10.6084/m9.figshare.20764843) to ensure reproducibility.

## 2 Results

The structure of the experimental results is described as follows: We first demonstrated the impact of reference for integration in Section 2.1, and then presented the overview of our reference‐free method Beaconet in Section 2.2. To fairly evaluate the performance of integration methods, we compared Beaconet and other methods using the proposed metrics PMD and other four popular metrics and illustrated the advantages of PMD by analyzing the difference between PMD and other metrics using the integration results in Section 2.3. The reference‐free property of Beaconet was evaluated in Section 2.4 by a comprehensive comparison with other methods using all possible references and merge orderings based on the PMD metric. The effectiveness of preserved molecular features was validated in Section 2.5. The computational efficiency of Beaconet was evaluated in Section 2.6. An ablation for demonstrating that the BS‐Norm module improves the performance of Beaconet was performed in Section 2.7.

### 2.1 The Impact of Selected References on the Integration Performance

**Figure 1:**
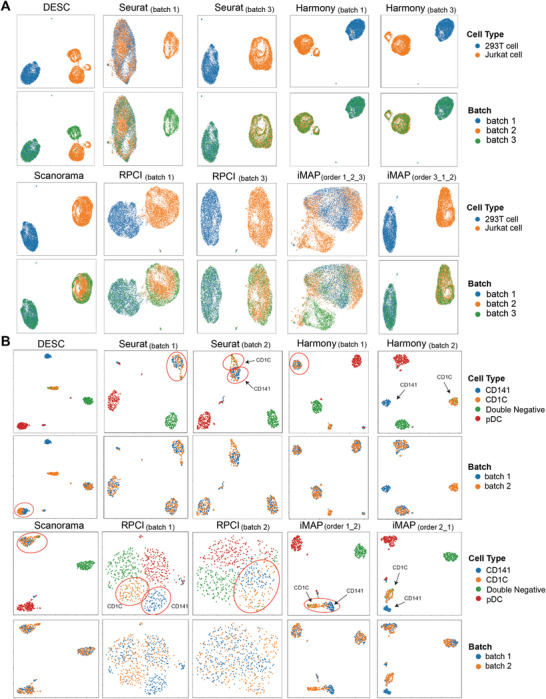
The dependence on the selection of references impacts integration performance. A) UMAP projection of integrated cell line datasets by DESC, Seurat, Harmony, RPCI, iMAP, and Scanorama. The panels of odd rows are colored by the cell types, and the panels of even rows are colored by the batches of datasets (see Figures S1 and S2 (Supporting Information) for all figures of 13 methods on cell line datasets). B) UMAP projection of integrated DC datasets by DESC, Seurat, Harmony, RPCI, iMAP, and Scanorama (see Figure S3 and S4 for all figures of 13 methods on DC datasets). The panels of odd rows are colored by the cell types, and the panels of even rows are colored by the batches of datasets.

To demonstrate the impact of selecting reference batches or the merge orderings on integration performance, we applied 13 well‐known methods, including Harmony,^[^ ^23^ ^]^ Seurat,^[^ ^29^ ^]^ MNNCorrect,^[^ ^17^ ^]^ iMAP,^[^ ^18^ ^]^ RPCI,^[^ ^22^ ^]^ FastMNN,^[^ ^17^ ^]^ LIGER,^[^ ^30^ ^]^ FIRM,^[^ ^31^ ^]^ BBKNN,^[^ ^32^ ^]^ scVI,^[^ ^33^ ^]^ scDML,^[^ ^26^ ^]^ DESC,^[^ ^25^ ^]^ and Scanorama^[^ ^24^ ^]^ on two integration tasks. The first task is to integrate three batches of cell line datasets, and the second task is to integrate two batches of human blood dendritic cell (DC) datasets. Among these methods, the first eight methods required a single reference batch or an ordering of reference batches, while the last five methods needed no reference. The integrated datasets of 13 methods using all possible (ordered) references (if required) were visualized by UMAP projection in Figures S1–S4 (Supporting Information). For the purpose of clarifying, we selected parts results of six representative integrated data for **Figure** **1**.

The results of the integration of cell line datasets (Figure 1A) show that the performance of reference‐based methods depends on the selected reference. Seurat, RPCI, and Harmony obtained three possible integrated results using different reference batches. MNNCorrect and FastMNN integrated datasets based on the ordering of batches, and got six integrated datasets. iMAP got nine integrated results as it received either individual batches or an ordering of batches for integrating datasets. We found that these methods integrated datasets well when batch 3 was selected as a reference or integrated in the early stage of the merge iterations. For example, Seurat and RPCI performed better when using batch 3 as a reference dataset compared to batch 1 or batch 2 (Figure 1A; Figure S1, Supporting Information). iMAP, FastMNN, and MNNCorrect performed well with the merge ordering “2_3_1”, but worse with the merge ordering “1_2_3” (Figure S1, Supporting Information). It may result that the data in batch 3 is more informative than the other two batches (File S2, Supporting Information). We noted that Harmony was slightly impacted by the selection of the reference batch, but it separated the Jurkat cells into two groups (Figure S1, Supporting Information), which were not observed in the three batches of original cell line datasets (Figure S2, Supporting Information). DESC although needs no reference for integrating datasets, the remaining batch effect of its integrated data could still be observed (Figure S1, Supporting Information). We further identified the clusters of cells using the integrated data of these methods by *k*‐means. The ARI scores and NMI scores also indicated that the performance of reference‐based methods varies in terms of the selection of the reference batches and ordering (Table S1, Supporting Information).

Using the two batches of human blood dendritic cell (DC) datasets, we further found that the selection of references also could impact the performance of reference‐based methods even when the batch effect is relatively small. We visualized all integrated results of the 13 methods on DC datasets by UMAP projection in Figures S3 and S4 (Supporting Information), and selected parts of the results of 6 representative methods in Figure 1B. The ARI scores and NMI scores are also provided in Table S2 (Supporting Information). These results indicated that reference‐based methods, such as Harmony, Seurat, MNNCorrect, iMAP, RPCI, and FastMNN, can yield two integrated results with varying performance depending on the chosen references or merge orderings. Furthermore, we observed that Seurat and Harmony achieved higher ARI scores when using batch 2 as a reference compared to batch 1, whereas iMAP and RPCI obtained higher ARI scores when using batch 1 as a reference instead of batch 2 (Table S2, Supporting Information). This discrepancy of preferred references indicated that the optimal choice of reference differs across batch effect removal methods. On the other hand, DESC and Scanorama produced unique integrated results without relying on the reference (Figure 1B; Figure S3, Supporting Information). DESC successfully preserved the distinction between the batch‐specific CDC141 DC and CD1C DC whereas the pDC in two batches was not mixed well (Figure 1B). We note that Scanorama mismatched the batch‐specific CD141 DC and CD1C DC, although avoiding the impact of reference selection (Figure 1B; Table S2, Supporting Information). This phenomenon is known as overcorrection or over‐alignment.^[^ ^24^, ^34^ ^]^

In summary, we demonstrated that the dependence on the selection of reference could lead to inconsistent integrated data with different integration quality on conditions of both large and small batch effects. Additionally, the reference‐based methods preferred different reference batches or orderings of the batches, suggesting there are two factors determining the selection of reference: the difference of captured information among batches and the computational methodologies of integration algorithms.

### 2.2 Overview of Beaconet

**Figure 2:**
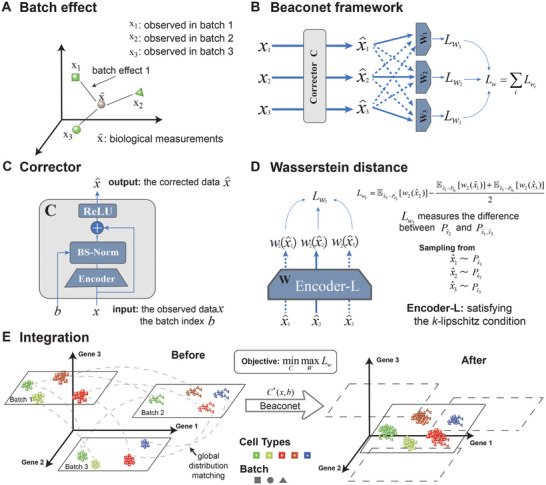
Overview of Beaconet. A) An illustration of a batch effect in three batches of datasets. B) The framework of Beaconet. An example for three batches of datasets. C) The architecture of the corrector for learning the correction function. D) An encoder satisfying *k*‐Lipschitz condition for estimating the Wasserstein distance between the distributions of batch 2 and the joint distribution of other batches, e.g., integrating 3 batches. E) The multiple batches of datasets with batch‐specific cell types are merged together in the original gene expression space.

The major characteristics of Beaconet is to integrating multiple batches of single‐cell datasets in original molecular feature space in a reference‐free manner (**Figure** **2**). As shown in Figure 2A, the observed single‐cell transcriptomic data was modeled as two independent components: the batch variations among different datasets (e.g., sequencing technologies, laboratories) and the underlying biological measurements. The main idea of Beaconet is to correct the batch effect among datasets using a correct function approximated by Corrector *C*, which is guided by Wasserstein distance‐based objective Equation (8). In the framework of Beaconet (Figure 2B), there are two components, including a corrector *C* (Figure 2C), a collection of auxiliary encoder‐L *W* = {*W*_1_,*W*_2_,…, *W_M_*} (Figure 2D). The Corrector *C* accepts log‐scaled gene expression features *x* and batch index *b* as its input and outputs the corrected data $\hat{x}$. The Corrector *C* consists of three components: a fully‐connected encoder that learns the unscaled correction vector from the expression data *x*, a BS‐Norm module to adjust the scale and bias of the learned correction vector for each batch *b*, and a ReLU function to filter the nonpositive expression values of the corrected data. Corrector *C* is learned with the guidance of the collection of encoder‐L *W*, in which each *W_i_* is a simple encoder with fully connected‐layers satisfying the *1*‐lipschitz condition. The lipschitz condition is useful for approximating the Wasserstein distance between the distribution of batch *i* and the joint distribution of the other batches in the objective function. The training process of Beaconet mainly consists of two steps: First, these encoder‐L in *W* are pretrained using the original uncorrected batches of data. Second, the corrector *C* and the collection of encoder‐L *W* are trained alternatively and cooperatively to learn the correction function for cells in mini‐batch manner. The changing of its integration ability during training process is visualized in Figure S18 (Supporting Information) for better understanding the behavior of Beaconet. After training, Beaconet is able to integrate the cells in the whole given datasets by the optimized corrector C* (Figure 2E). After integration, batch effect of the given batches of datasets are corrected, and the integrated dataset is ready for downstream analysis. The details of Beaconet is available in Section 5 (Methods), and our implementation of Beaconet using Python is available at GitHub (https://github.com/GaoLabXDU/Beaconet).

### 2.3 Positive Merge Divergence is Effective to Evaluate Integration Performance

**Figure 3:**
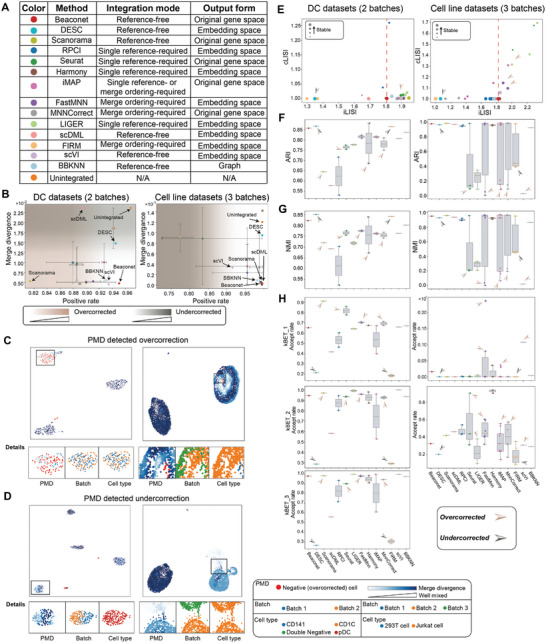
Comparison of PMD with NMI, ARI, kBET and LISI. For the methods that require a single reference or the ordering of batches, we run them with all possible references (orderings). For all single‐reference‐based methods, we run 2 times on DC datasets, three times on cell line datasets, and five times on human pancreatic datasets for traversing all references. For all merge‐ordering‐based methods, we run them two times on DC datasets, and 6 times on cell line datasets for traversing all merge orderings. A) Description of Beaconet and other 13 integration methods. B) PMD evaluated the performance of Beaconet and 13 compared methods on two‐batch DC datasets and three‐batch cell line datasets. C) PMD detected overcorrection on the integrated data (Scanorama). D) PMD detected under‐correction on the integrated data (DESC). E) LISI evaluated the performance of Beaconet and 13 integration methods on two‐batch DC datasets and three‐batch cell line datasets. F) ARI evaluated the performance of Beaconet and 13 integration methods on two‐batch DC datasets and three‐batch cell line datasets. G) NMI evaluated the performance of Beaconet and 13 integration methods on two‐batch DC datasets and three‐batch cell line datasets. H) kBET evaluated the performance of Beaconet and 13 integration methods on two‐batch DC datasets and three‐batch cell line datasets.

To fairly evaluate the integration performance of the integration method, we compared the effectiveness of the proposed PMD metric and four popular quantitative metrics, including kBET, LISI (i.e., cLISI and iLISI), ARI, and NMI, to analyze the results of 13 existing integration methods described in **Figure** **3**A and Beaconet on two integration tasks, integrating the two‐batch DC datasets and the three‐batch cell line datasets.

As shown in Figure 3B, PMD can clearly exhibit how the integration methods balance batch effect removal and bio‐conservation by separating these two goals onto two orthogonal axes. For example, Scanorama had both a low positive rate and merge divergence on integrated DC datasets, suggesting it may tend to mix the cells in different batches more than preserving the purity of cell types. On both two integration tasks, DESC had a higher positive rate and merge divergence, suggesting it may tend to preserve the purity of cell types but the remaining batch variations were more than other methods. The behavior of the reference‐based method was affected by the selected reference, which failed to integrate the multiple‐batches of single‐cell datasets when using unfavorable reference (Figures S5–S7, Supporting Information). The failed integration is typically indicated by a lower positive rate or higher merge divergence, which means that the purity of cell types was disturbed and the batch variations were not reduced respectively. The best integration method appears at the bottom right corner of Figure 3B. To intuitively illustrate PMD detecting overcorrection and undercorrection, we visualized the PMD metric on the integrated data of Scanorama (Figure 3C) and DESC (Figure 3D). The mismatched cells were identified as negative cells and colored red. The cells merged with the same cell type are identified as positive cells, the deeper blue color indicated the better mixture of batches. More cases were provided in Figures S5–S7. LISI is an existing popular metric assessing integration methods using two sub‐metrics, iLISI for mixing of batches and cLISI for purity of cell types. In this framework, well‐integrated data was expected to get higher iLISI and lower cLISI, that is, the bottom right corner in Figure 3E. However, compared to the optimal integrated data, the overcorrected data may get a better iLISI score by slightly worsening the cLISI score. For example, the integrated results, such as Harmony using batch 1 as a reference, Scanorama and Seurat on DC datasets, mismatched the batch‐specific CD1C and CD141 cells (Figure 3C; Figure S3, Supporting Information) but appeared in the bottom left corner in the left panel of Figure 3E. NMI and ARI were clustering metrics and applied as batch effect removal metrics in recent studies.^[^ ^9^, ^10^ ^]^ The higher ARI and NMI scores were expected to be assigned to the better‐integrated data (Figure 3A; Tables S1 and S2, Supporting Information). However, they can only detect the batch variations that distort the structure of clusters, but ignore the batch variations within the same cluster. It thus failed to distinguish the optimal integration data and under‐corrected data (Figure 3F,g; Figures S5 and S6, Supporting Information). kBET was a hypothesis testing‐based metric measuring batch effect by the accept rate (or reject rate). The well‐integrated data was expected to get a higher accept rate (or lower reject rate). We applied kBET in three different strategies in a previous study,^[^ ^9^, ^22^, ^35^ ^]^ including directly calculating, averaging of kBET scores by cell type, and down sampling by cell type before calculating (we called them kBET_1, kBET_2, and kBET_3 below). As kBET only considered whether the batches were fully mixed, it failed to distinguish overcorrected data and optimal integrated data (Figure 3H).

In summary, PMD decomposes the overcorrection and under‐correction into two orthogonal axes and thus is fairer for evaluating the integration methods on datasets with non‐identical cell compositions, especially when batch‐specific cell types exist. Cluster‐based metrics, such as ARI and NMI, are related to the downstream clustering analysis, but ignore the batch effect within clusters. kBET and LISI can capture the fine‐grained batch effect at the individual‐cell level but may fail to identify overcorrected results. PMD metric shows Beaconet outperforms or comparable the existing methods with their most suitable reference on the two integration tasks (Figure 3B). We note that, despite the difference in these metrics, they are consensus that the selection of reference can lead to inconsistent integration results.

### 2.4 Beaconet Accurately Integrates Single‐Cell Datasets Without Reference

**Figure 4:**
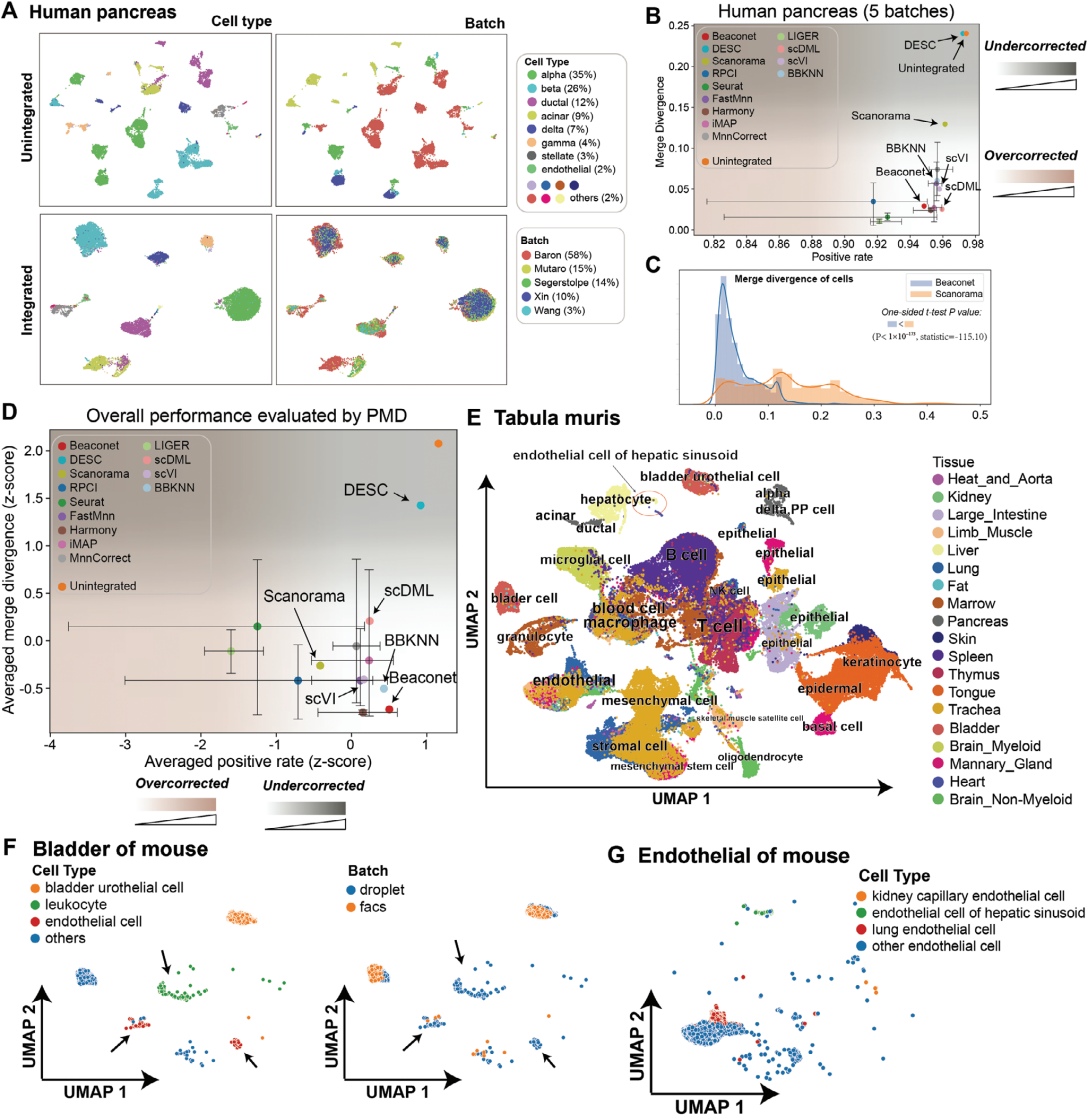
Beaconet accurately integrates multiple batches of datasets without reference. A) UMAP projection of the five human pancreatic datasets before and after integration by Beaconet. B) PMD evaluated Beaconet, five reference‐free methods, and 7 reference‐based methods with all possible references on integrated five human pancreatic datasets. C) Comparing the distribution of merge divergence of positive cells in the integrated human pancreatic datasets processed by Beaconet and Scanorama. The details of a statistical test are available in Section 5.4: Statistical Analysis. D) The overall performance comparison of Beaconet and 12 integration methods using the averaged PMD scores on the three integration tasks. The error bar indicates the uncertainty of performance caused by the selection of reference (ordering). E) UMAP projection of the 2‐batch Tabula muris datasets integrated by Beaconet. The tissues are indicated with different colors and the major cell types are annotated. F) UMAP projection of an individual tissue (bladder) in Tabula muris (See all figures of 19 tissues in Figures S12 and S13, Supporting Information). The cell types are indicated by different colors. g) UMAP projection of four distinct cell types of endothelial cells across all tissues in integrated data.

In this section, we validated the reference‐free integration performance of Beaconet qualitatively and quantitatively. We applied Beaconet and 12 existing methods (FIRM was excluded due to its computational complexity) for integrating five human pancreatic datasets. These datasets were generated from five different experimental laboratories^[^ ^36^, ^37^, ^38^, ^39^, ^40^ ^]^ using four different sequencing protocols, including inDrops,^[^ ^41^ ^]^ CEL‐seq2,^[^ ^42^ ^]^ SMART‐seq2^[^ ^43^ ^]^ and SMARTer,^[^ ^44^ ^]^ and were clearly separated by batch label (**Figure** **4**A).

The single reference batch‐based methods (e.g., Seurat, Harmony) generated 5 different results with different reference batches, and the merge ordering‐based methods (e.g., FastMNN) generated 120 results with different ordering of batches, especially iMAP had 125 (i.e., 125 = 120+5) different results at most. As shown in Figures 4B and S7 (Supporting Information), the integration performance of reference‐based methods was influenced by the selection of reference batches or the orderings of batches, which appear to arise from the difference in captured cell types of the five datasets (File S2, Supporting Information). These methods performed better when the reference was fitting, but worse when the reference was unfitting (Figure 4B; File S3, Supporting Information). By comparing with altogether 392 different integrated results of the 12 existing methods, we found that, in a reference‐free manner, Beaconet achieved nearly the optimal integration performance compared with reference‐based methods (Figure 4A,B) on the integration of multiple human pancreas datasets. We noted that the optimal results for the reference‐based methods were the upper bound of them, which were achieved by traversing all possible references and may be difficult to achieve in practice, especially when lacking necessary background knowledge for the selection of references for specific algorithms on novel integration tasks. Besides Beaconet, there are other five reference‐free methods, including Scanorama, DESC, BBKNN, scVI, and scDML. As shown in Figure 4B, BBKNN, outputting an integrated cell‐cell graph, performs well across all three tasks and is ranked just after Beaconet in overall performance. scDML achieved the best performance in the integration of the five‐batch human pancreatic datasets, followed by Beaconet, but it failed to integrate the two DC datasets (Figure 3B). Beaconet consistently performed well across the three tasks, exhibiting its superior integration capacity regardless of task characteristics. Furthermore, out of all compared methods, only Scanorama and Beaconet have properties of both reference‐free and original gene space correction, however, Beaconet also outperforms Scanorama with a clear margin (Figure 4B,C). To draw a more general conclusion, we summarized the PMD scores of all methods on the three integration tasks after eliminating the difference of the numerical scaled evaluation scores among different tasks by z‐score (Figure 4D). We found Beaconet outperforms existing methods on the overall performance, even for most optimal integration results using reference‐based methods using their favorable (ordered) reference(s) for each respective task (Figure 4D; File S5, Supporting Information). To further confirm the capacity of Beaconet to relieve the overcorrection, we manually removed five cell types in batch “Baron” in turn, including the alpha cells, beta cells, delta cells, ductal cells, and gamma cells, and applied Beaconet to integrate the “Baron” batch and other four batches. The results show that Beaconet still integrates the five batches of data correctly, which did not mismatch these cell types in other batches to batch “Baron” (Figure S8, Supporting Information).

Furthermore, we applied Beaconet to integrate two mouse atlas datasets in Tabula Muris,^[^ ^3^ ^]^ which contains cells of 19 tissues sequencing from two distinct technical approaches, droplet (microfluidic droplet‐based 3’‐end counting) and FACS (full‐length transcript analysis based on fluorescence‐activated cell sorting). In the two batches of datasets, there were batch‐specific tissues, such as limb_muscle, pancreas, brain_myeloid, and brain_non‐myeloid (Figure S9; File S2, Supporting Information). In the batch‐common tissues, there were both batch‐specific cell types and batch‐common cell types (File S2, Supporting Information). For example, in the bladder tissue, the leukocyte and endothelial cells were batch‐specific cell types of the “droplet” batch, while bladder urothelial cells are sequenced by both of “FACS” and “droplet” batch (Figures S10 and S11; File S2, Supporting Information). In mammary gland tissue, macrophage, B cell, and T cell were batch‐specific cell types of “droplet” batch, while basal cell, endothelial cell, luminal epithelial cell of mammary gland, and stromal cell were batch‐common cell types (Figures S10 and S11; File S2, Supporting Information). Beaconet correctly merged the batch‐common tissues, which were separated by the batch effect in unintegrated data, and distinguished the batch‐specific tissues (Figure 4E; Figure S9, Supporting Information). Moreover, in the individual batch‐common tissue, Beaconet mixed the batch‐common cell types and preserved the batch‐specific cell types for these tissues. For example, Beaconet mixed the batch‐common bladder urothelial cells from different platforms and preserved the batch‐specific leukocyte and endothelial cells in the ‘droplet’ batch (Figure 4F). We provided the results for each tissue in Figures S12 and S13 (Supporting Information), which suggested Beaconet enables accurately integrating the single‐cell transcriptomic datasets that contained batch‐specific cell types. Besides this, we noticed that Beaconet could also recognize the difference between the major endothelial cells and other distinct subtypes of endothelial cells, including the kidney capillary endothelial cells, endothelial cells of the hepatic sinusoid, and lung endothelial cells (Figure 4G), which mainly existed in kidney, liver, and lung (File S2, Supporting Information).

### 2.5 Beaconet Enables Preservation of Original Molecular Features

**Figure 5:**
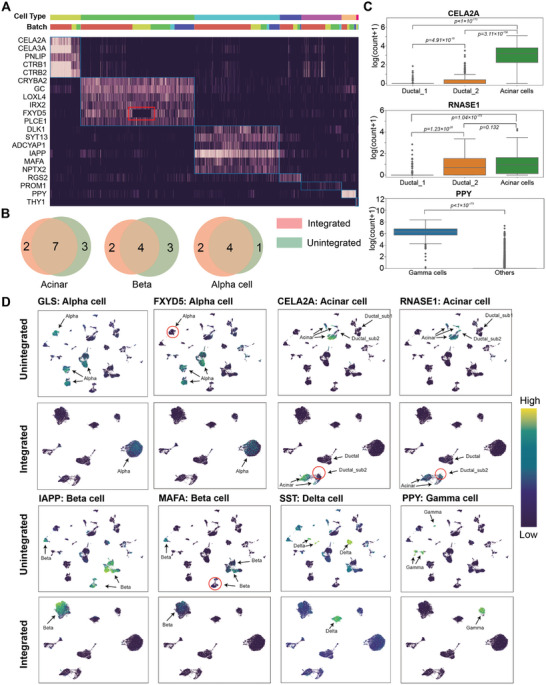
The integrated molecular feature space of Beaconet is effective for characterizing the heterogeneity of cells. A) The heat map of marker genes of major cell types on the integrated human pancreatic datasets. B) The overlap of detected marker genes in top‐10 differentially expressed genes of integrated and original data. C) The integrated data preserves the difference of the features of the two subpopulations of ductal cells and conserves the expression pattern of PPY of gamma cells. The details of a statistical test are available in Section 5.4: Statistical Analysis. D) Comparison of the integrated and unintegrated gene features using UMAP projection, including *GLS, FXYD5, CELA2A, RNASE1 IAPP MAFA SST*, and *PPY*.

In this section, we tested whether the preserved gene‐expression features in integrated data are effective by further investigating the features in integrated five‐batch human pancreatic datasets. **Figure** **5**A preliminary shows that, in the integrated data, the marker genes derived from the CellMarker database^[^ ^45^ ^]^ could identify the major cell types in the human pancreas. We then compared the expression pattern of the differentially expressed genes and marker genes on integrated and unintegrated data (Figure S14, Supporting Information), suggesting the molecular features in the integrated data of Beaconet are effective for characterizing cells while removing the batch variations among these datasets (Figure 5D). The top‐10 differentially expressed genes for each cell type on both integrated data and unintegrated data were reported in Tables S3–S17 (Supporting Information), in which the genes were detected and ranked using function ‘scanpy.tl.rank_genes_groups’ in scanpy.^[^ ^46^ ^]^ For detecting differentially expressed genes and finding marker genes, the molecular features preserved by Beaconet are comparable to the original expression features, in which we used the marker genes derived from the CellMarker database as the gold standard. For example (Figure 5B), Beaconet detected nine marker genes in top‐10 differentially expressed genes for acinar cells, including *REG1A, SPINK1, CTRB2, CPA2, PRSS1, CTRC, CTRB1, PNLIP* and *PRSS3*, in which seven marker genes are detected in both of the integrated and original data, and two are integrated‐specific marker genes, including *PNLIP* ^[^ ^37^, ^47^, ^48^ ^]^ and *PRSS3*.^[^ ^48^ ^]^ For alpha cells, in the top‐10 differentially expressed genes, there are six marker genes detected by the integrated data, and five marker genes in original unintegrated data, in which *IRX2, GC, TTR, GCG* are detected by both integrated and unintegrated data and *GLS*,^[^ ^48^ ^]^ *LOXL4* ^[^ ^37^, ^48^ ^]^ are integrated‐specific. Gamma cells accounted for only 4% of the total number of cells in the human pancreatic datasets (Figure 4A), which is one of the rarest pancreatic islet cell‐type.^[^ ^49^ ^]^ Beaconet still clearly preserved the cluster of gamma cells (Figure 5D) and its marker gene, *PPY*, as the top‐1 differentially expressed gene in integrated data (Figure 5C; Table S5, Supporting Information).

The molecular features preserved by Beaconet are also able to distinguish the subpopulation of the same cell type. In the literature of the “Baron” dataset,^[^ ^36^ ^]^ it was found that the ductal cells had two subpopulations, in which one connected to the acinus and the other formed the terminal duct.^[^ ^36^, ^50^ ^]^ In the original dataset, the first subpopulation is closer to acinar cells in UMAP projection (Figure 5D) and highly expressed a marker gene of acinar cells, *RNASE1* (Figure 5C,D), while the second subpopulation of ductal cells did not highly express *RNASE1*. The acinar cells and the first subpopulation of ductal cells could be distinguished by the marker gene of acinar cells, *CELA2A* (Figure 5C,D). In the integrated data of Beaconet, the difference between the 2 distinct subpopulations of ductal cells was preserved (Figure 5C,D). Furthermore, *RNASE1* is still highly expressed in the first subpopulation of ductal cells, while not expressing in the second subpopulation. The first subpopulation of ductal cells is closer to the cluster of acinar cells of their higher similarity in the original data,^[^ ^36^ ^]^ however, we could also distinguish acinar cells from the first subpopulation of ductal cells by *CELA2A* (Figure 5C). These results suggested that Beaconet was able to preserve the expression pattern of rare cell types and the subpopulations of the same cell type. It is worth noting that Beaconet does not impose to obscure the characteristics of individual genes in integrated data but merges the datasets by aligning the global distribution of data in different batches. For example, there were usually batch‐specific marker genes in different datasets. Before integration, one marker gene of alpha cells, *FXYD5*, was not highly expressed in the “Mutaro” batch (Figure 5D; Figure S14, Supporting Information). After being integrated by Beaconet, the alpha cells from five batches were merged well, and the alpha cells in the “Mutaro” batch were not imposed to highly express *FXYD5* (Figure 5A; Figure S14, Supporting Information). UMAP visualization of over 180 marker genes before and after integrated is available in Figshare (https://doi.org/10.6084/m9.figshare.20764843). In summary, the molecular features in the integrated data of Beaconet could not only characterize cell types and subpopulations but also enable differential expression analysis on the human pancreatic datasets.

### 2.6 Beaconet is Efficient for Large‐Scale Datasets

In terms of large‐scale datasets, the common strategy^[^ ^23^, ^26^, ^27^, ^51^ ^]^ to improve efficiency is to integrate the data in inexplicable embedding space and match cell pairs by approximate nearest neighbor searching methods. In contrast to them, Beaconet directly removes the batch effect in the high‐dimensional molecular feature space, which is a more difficult computation task.

**Figure 6:**
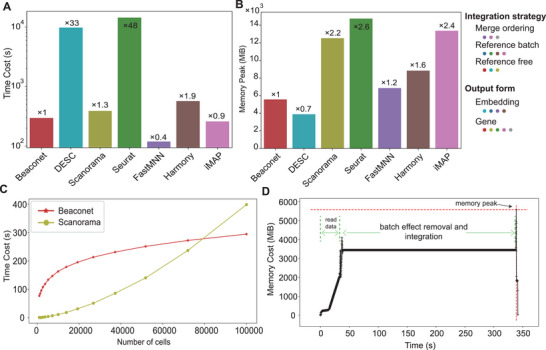
The computational efficiency of Beaconet to integrate the 2 datasets sequenced by distinct platforms in large‐scale Tabula Muris. A) Comparison of the time cost of Beaconet, DESC, Scanorama, Seurat, FastMNN, Harmony, and iMAP on the full Tabula Muris. B) The comparison of peak memory consumption of Beaconet, DESC, Scanorama, Seurat, FastMNN, Harmony, and iMAP on the full Tabula Muris. C) The comparison of time cost‐cell number curves of Beaconet and Scanorama on down‐sampling Tabula muris. D) The memory consumption of Beaconet is stable during the processing of integration (see Figure S15, Supporting Information for the results of other methods).

To evaluate the computational efficiency of Beaconet, we assessed the efficiency of Beaconet by comparing it with 6 efficient batch‐effect removal methods, including Scanorama, Seurat, iMAP, DESC, FastMNN, and Harmony, on the large‐scale Tabula muris, which contains the single‐cell transcriptomic data of 100 605 cells in total.^[^ ^3^ ^]^ In the baseline methods, Scanorama, Seurat, MNNCorrect, and iMAP integrated data in the gene‐expression space, and Harmony, RPCI, FastMNN, and DESC integrated data in low‐dimensional embedding space. We run Beaconet five times to exclude the jitter of computer operation efficiency. The results show that the time consumption of Beaconet was stable, integrating the two datasets using 296(±2) seconds. As shown in **Figure** **6**A, Beaconet was the fastest in three reference‐free methods, followed by Scanorama (388 s). In the gene‐expression space batch‐effect removal methods, the time consumption of Beaconet was secondary to iMAP (258 s), which used an individual batch as a reference to integrate datasets (Figure 6A). Seurat integrated data well in about 4 h using a batch of data as a reference. FastMNN is the fastest method (119 s), which integrated datasets in embedding space using an ordering of batches (Figure 6A).

To further investigate the time cost curve of Beaconet with respect to the number of cells, we tested Beaconet on down‐sampled Tabula Muris with sampling rate $\frac{10^{\theta }}{100605}$, where θ the change from 3 to 5 with 15 equal intervals, and compared with Scanorama. We selected Scanorama as the baseline because it was an efficient method for integrating datasets in the original feature space without reference.^[^ ^24^ ^]^ Figure 6C shows that, with the number of cells increasing, the time consumption of Beaconet grew slower than Scanorama. To assess the memory efficiency of Beaconet, we monitored the running memory of each method and recorded the used memory every 0.1 s. The peak memory cost of Beaconet and these existing methods on full Tabula Muris is shown in Figure 6B, indicating that Beaconet used the lowest memory (5570 MiB) in gene‐expression space batch‐effect removal methods. DESC used the lowest memory in these nine methods by embedding cells into a low‐dimensional space. The peak memory cost of Scanorama, iMAP, and Seurat (the other three gene‐expression space batch‐effect removal methods) were 12525 MiB, 13374 MiB, and 14750 MiB respectively. Figure 6D shows that the memory cost of Beaconet was stable in the integration process and the peak of memory usage was reached when inferencing its result. The fluctuation of the memory usage of other methods is available in Figure S15 (Supporting Information). We noted that the running time of Beaconet and these baseline methods were compared by performing a single run, while the reference‐based methods may need to be run many times using different (ordered) references for selecting and obtaining a reliable result in practice. Thus, as a reference‐free method, Beaconet may be more efficient in practice. In summary, Beaconet, in gene‐expression space and in reference‐free manner, achieved superior computational efficiency in speed and memory cost, being capable of integrating the large‐scale atlas datasets.

### 2.7 BS‐Norm is Necessary for Improving the Integration Performance of Beaconet

It was practical to design task‐related architecture to improve the effectiveness of neural networks in specific computational tasks.^[^ ^52^, ^53^, ^54^ ^]^ To better capture the potential batch variations of cells in different batches, we designed BS‐Norm (Batch‐Specific Normalization) in the corrector to improve the performance of Beaconet.

**Figure 7:**
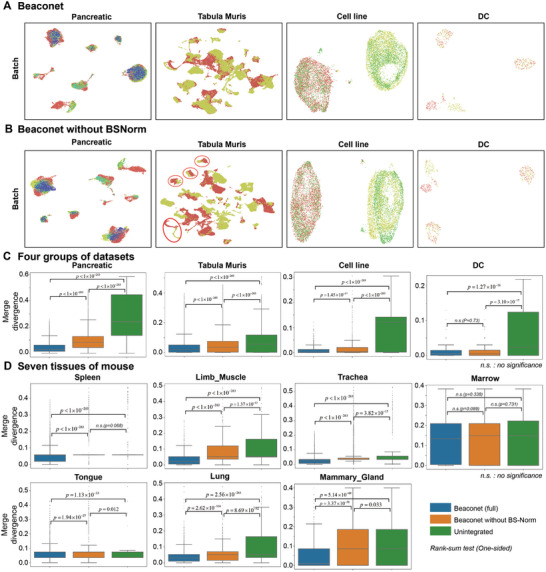
Ablation study for demonstrating the improvement of Beaconet by BS‐Norm. The details of a statistical test are available in Section 5.4: Statistical Analysis. A) UMAP projection of integrated five‐batch human pancreatic datasets, 2‐batch Tabula muris, three‐batch cell line datasets, and 2‐batch DC datasets using Beaconet. Each panel is colored by the batch labels of datasets. B) UMAP projection of integrated five‐batch human pancreatic datasets, two‐batch Tabula Muris, three‐batch cell line datasets, and 2‐batch DC datasets using the variant Beaconet without BS‐Norm. Each panel is colored by the batch labels of datasets. C) Comparing the distribution of merge divergence of the full Beaconet and the variant Beaconet without BS‐Norm on four groups of datasets, including five batches of human pancreatic datasets, two mouse atlas datasets in Tabula Muris, three batches of cell line datasets and two batches of DC datasets. D) Comparing the distribution of merge divergence of the full Beaconet and the variant Beaconet without BS‐Norm on seven mouse tissues, including marrow, limb_muscle, trachea, spleen, tongue, lung, and mammary_gland.

To empirically demonstrate the necessity of BS‐Norm for Beaconet, we compared the full Beaconet and a variant of Beaconet that disables the BS‐Norm module on four tasks, including two‐batch Tabula Muris, five‐batch human pancreatic datasets, three‐batch cell line datasets, and 2‐batch DC datasets. For Tabula Muris, we assess Beaconet and the variant Beaconet on both full datasets and seven groups of the largest sub‐datasets with individual tissue, including marrow, limb muscle, trachea, spleen, tongue, lung, and mammary gland. We first visualized the integrated data of Beaconet (full) and the variant Beaconet by UMAP projection in **Figures** **7**A,B and S16 (Supporting Information). These figures show that both Beaconet (full) and the variant Beaconet can reduce the batch effect among datasets, while Beaconet (full) performed better than the variant Beaconet. For example, on the five‐batch human pancreatic datasets, the variant Beaconet made the cluster centers of the same cell types in different batches overlap with each other, while the full Beaconet mixed them batches better (Figure 7A,B). On the integrated cell line datasets, the variant Beaconet made the cell types in different batches overlapped correctly but the batch variation of Jurkat cells still was observed. To confirm the improvement of BS‐Norm for Beaconet, we then used PMD metric to compare full Beaconet with the variant method and performed both the permutation test (Table S19, Supporting Information) and Wilcoxon rank‐sum test (Table S20, Supporting Information) to assess whether Beaconet reduced the merge divergence for cells by leveraging BS‐Norm. The boxplot and histogram show (Figure 7C; Figure S17, Supporting Information), with comparable positive rate (Table S18, Supporting Information), the distributions of merge divergence of Beaconet (full) were significantly decreased on these integrated datasets, except the integrated Marrow dataset and DC dataset. These results demonstrated that BS‐Norm in the corrector of Beaconet improves the performance of Beaconet. We note that Beaconet (full) and the variant Beaconet did not show significant differences in the merge divergence (rank‐sum test P = 0.73; one‐sided permutation test P = 0.47; 2‐sided permutation test: P = 0.955) on the integrated DC dataset. They both correctly merged the batch‐shared pDC and double negative cell respectively, preserved the difference between batch‐specific CD141 DC and CD1C DC (Figure 7A–C; Figure S16, Supporting Information), suggesting that the ability to suppress the overcorrection of Beaconet may be independent from the BS‐Norm module. In summary, the designing of the BS‐Norm module could significantly improve the integration performance of Beaconet, and both Beaconet with or without BS‐Norm enables integrating datasets from different batches.

## 3 Discussion

Batch effect correction is critical for the integrative analysis of single‐cell transcriptomic datasets produced by different platforms or experimental laboratories.^[^ ^9^, ^13^ ^]^ When integrating multiple batches of datasets, the selection of reference batches or orderings of datasets has a significant impact on the performance of most reference‐based batch‐effect removal methods, since the captured information in different datasets typically is different (e.g., in terms of the number of cells and the composition of cell types).^[^ ^17^ ^]^ The inappropriate reference for the integration may lead to negative consequences for downstream analysis tasks such as clustering,^[^ ^25^ ^]^ visualization,^[^ ^9^ ^]^ and cell type identification.^[^ ^55^, ^56^ ^]^ Furthermore, it is difficult to recommend an ordering of reference that works well for batch‐effect removal methods, since the number of possible orderings of the batches increases exponentially with the number of batches (e.g., there are 5! = 120 possible ordering for the 5‐batch human pancreatic datasets). Our study also suggests that different methods may prefer different reference batches or orderings of batches (Figure 1A,B; Figure 3B–E; Figure 4B; Figures S1–S7; Tables S1–S3, Supporting Information). Besides this, the state‐of‐the‐art methods were typically designed to address the integration problem in a compact embedding space to improve computational efficiency.^[^ ^23^, ^25^, ^26^, ^27^, ^34^, ^57^ ^]^ However, recent studies^[^ ^14^, ^26^ ^]^ indicate that it limits the potential downstream applications of integrated data to project datasets into embedding space.

In this paper, we have introduced a reference‐free method, Beaconet, for integrating multiple batches of single‐cell transcriptomic data in the original molecular feature space. There are two major innovations in Beaconet. First, it integrates scRNA‐seq datasets without reliance on reference. Second, Beaconet could preserve the gene‐expression features for more potential downstream analysis. By comparing with 13 representative batch‐effect removal methods on several real‐world datasets, ranging from single tissue to multiple tissues, we demonstrated that Beaconet is capable of removing the batch effect in multiple batches of single‐cell transcriptomic data in a reference‐free manner efficiently and effectively. Besides the biological difference of cell types, the distinct subpopulations of the same cell types are preserved in the integrated data (Figure 5D). Moreover, Beaconet can preserve the original molecular features, allowing performing gene‐based analysis (e.g., detecting differentially expressed genes) using the integrated data (Figure 5; Figure S14, Supporting Information). Another advantage of Beaconet is that Beaconet could achieve superior integration performance without fine‐tuning hyper‐parameters for specific datasets. We fixed all hyper‐parameters as default values for all datasets in our experiments, which differ in the volume of cells, the diversity of cell types, the number of batches, and the sequencing technologies, to emphasize the utility of Beaconet in practice. These results show Beaconet is particularly beneficial for researchers who lack prior knowledge about the selection of reference batches or hyper‐parameters of machine learning to integrate multiple batches of single‐cell transcriptomic datasets. Overall, Beaconet is a powerful and practical batch effect removal method for the integration of multiple datasets, and thus, facilitates the understanding of heterogeneity of cells in the single‐cell transcriptomic data analysis.

From a methodological perspective, we supposed that the accurate integration for batch‐specific cell types may be related to the global correction function, which may leverage the information of major batch‐common cell populations to improve the integration performance of batch‐specific cell populations. In the results of our experiments, we observed that, besides Beaconet, three of the previous methods (i.e., RPCI, Harmony, and iMAP) and the variant Beaconet disabling BS‐Norm also exhibit the ability to suppress overcorrection with suitable reference (if required). These methods share a common property of employing some global process for all cells in different batches. For example, Harmony integrates datasets using a global linear mixed correction function. On the other hand, the local cell pairs‐based methods may take a risk on the mismatched cell pairs in a small number of matched cell pairs (also reported by Wang et. al.^[^ ^18^ ^]^ and Yang et. al.^[^ ^21^ ^]^). Based on our observation of these methods, we supposed that it is crucial for solving the overcorrection problem to utilize a global smooth function for cells in each batch. It is an important direction to explore better training strategies for global correction functions for balancing the risk of under‐correction and overcorrection, as it is hard to distinguish whether the unlabeled cells are batch‐common or batch‐specific. We hope this observation may serve as inspiration for exploring novel batch‐effect removal methods.

One limitation of Beaconet is that its time complexity is quadratic in terms of the number of batches, and Luecken MD et al.^[^ ^14^ ^]^ shows that the number of batches in single‐cell datasets is increasing rapidly in the research community of single‐cell omics. When the number of batches is particularly large, the time complexity for batch number is a bottleneck for Beaconet. The quadratic time complexity of Beaconet mainly results from the need to construct linear interpolation sampling from each pair of datasets to maintain the encoder‐L satisfying the *k*‐Lipschitz condition. Therefore, we anticipate more efficient, useful, and accurate batch‐effect removal methods for the single‐cell datasets will be proposed, following the idea of reference‐free and original feature space integration. In this study, our main focus has been on the integration of single‐cell transcriptomic data from multiple sources. However, recent research^[^ ^14^ ^]^ indicates that most batch‐effect removal methods perform even worse than unintegrated data when transferring the application scenario from single‐cell transcriptomic data to scATAC‐seq data, due to its special challenges (e.g., close‐to‐binary nature, extreme sparsity and higher dimensions).^[^ ^58^ ^]^ Consequently, it is a meaningful direction to improve Beaconet for integrating such more challenging omics data and analyzing its effectiveness for future exploration.

## 4 Conclusion

Most batch effect removal methods for multiple batches of single‐cell transcriptomic data rely on a given reference batch or ordering of batches and need to project data into embedding space. In this study, we present a reference‐free method, Beaconet, for integrating single‐cell transcriptomic data in the original feature space. To evaluate its performance, we compared Beaconet with 455 integrated outputs obtained from 13 existing batch‐effect removal methods, including eight well‐known existing reference‐based methods with all possible reference batches or ordering of batches and five popular reference‐free methods. We have demonstrated that Beaconet achieves superior performance to existing methods, even for most reference‐based methods with any possible reference. Besides this, Beaconet also preserves the original molecular feature space. Beaconet is an effective and efficient batch effect removal tool for integrating multiple batches of datasets and thus facilitates the understanding of cell heterogeneity in the analysis of single‐cell transcriptomic data. We also verified the necessity of the designing of the architecture of Corrector in Beaconet by ablation experiments. Additionally, PMD metric is useful to assess batch‐effect removal methods, especially on datasets with batch‐specific cell types, which is capable of complementing the existing evaluation strategies for the batch‐effect removal problem.

## 5 Experimental Section

### Problem Formulation

Given *X* = {*X*_1_,*X*_2_,…, *X_M_*} the collection of *M* unlabeled single‐cell transcriptomic datasets, in which each dataset $X_{i}\in R^{n_{i}\times p}$ contains the log‐scaled single‐cell transcriptomic data with *p* genes of *n_i_* cells. The aim of this work was to learn a correction function for cells of the given dataset *X* to remove the batch effect among these datasets in the original feature space.

*Wasserstein distance*. Suppose there were 2 batches of datasets with expression matrices, *X*_1_ and *X*_2_, that follow 2 unknown probability distributions *P*_1,_ and *P*_2_ respectively. The batch effect as the unwanted variations in single‐cell datasets caused by handling the cells in distinct batches can be roughly measured by the “distance” between the 2 probability distributions.^[^ ^18^ ^]^ Thus, the aim of removing the batch effect can be approximated to fit a smooth function that minimizes the measured difference of the distributions that the cells in datasets. While there were many metrics for probability distributions, such as KL divergence and JS divergence, the Wasserstein distance *D*(*P*_1_,*P*_2_) between 2 distributions *P*_1,_ and *P*_2_was choosen, because the Wasserstein distance allows us to avoid the unstable problem of adversarial learning by inducing a weaker topology when it makes it easier for a sequence of distribution to converge comparing to other measurements for the difference of distributions.^[^ ^19^, ^59^ ^]^

(1)

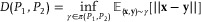

where π(*P*_1_,*P*_2_) is the set of all possible joint distributions with the marginal distributions *P*_1_ and *P*_2_, γ is one of the possible joint distributions *P*_1_ and *P*_2_, || · || represents the *l*_1_‐norm, the infimum is taken over all possible joint distributions in π(*P*_1_,*P*_2_), ***x*** and ***y*** are the random samples following distribution γ. Because it is intractable to calculate Wasserstein distance by its definition, the form of Wasserstein distance is equivalently reformulated refer to Arjovsky M et. al.,^[^ ^19^ ^]^

(2) $$D\left(P_{1},P_{2}\right)=\underset{\left||W\right|{|}_{L}\leq k}{\sup}E_{x∼P_{1}}\left[W\left(x\right)\right]-E_{y∼P_{2}}\left[W\left(y\right)\right]$$

where *W* is an auxiliary function for estimating the difference of the two distributions, the supremum is taken over all functions *W* satisfying the *k*‐lipschitz condition. In this formulation, this difference can by approximated by neural networks in a differentiable way based on the random sampling cells from *X*_1_ and *X*_2_ without explicitly estimating the distributions. Furthermore, the gradient information in the network *W* can guide the optimization of the correction function. The research of Arjovsky et. al. (WGAN)^[^ ^19^ ^]^ demonstrates the power of Wasserstein distance for adversarial generative learning, which aims to generate novel samples following the same distribution (the distribution was typically unknown and hard to estimate) with finite training datasets. Wang, et. al.^[^ ^18^ ^]^ applied a variant of WGAN on batch effect correction problems in a reference‐based manner by rwMNN‐pair‐based training. Inspired by these 2 articles, Beaconet was proposed, which was a reference‐free method for batch effect correction of scRNA‐seq data and does not rely on reference and the identification of cell pairs such as rwMNN pairs.

*Penalty term for 1‐lipschitz condition*. As proven by Gulrajani, I. et al.,^[^ ^60^ ^]^ there exists a 1‐Lipshitz function W* that is the optimal solution of 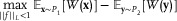. Assuming π is the optimal joint distribution of *P*_1_ and *P*_2_, which is defined by Equation (1). $P_{(x,y)∼\pi }\left[\nabla W^{*}\left(x_{t}\right)=\frac{y-x_{t}}{||y-x_{t}\left|\right|}\right]=1$ is guaranteed if π(x = y) = 0 and x_*t*_ = (1 − *t*)x + *t*y, in which *t* ∈ [0, 1]. Then, the gradient norm of W* is 1 almost everywhere under *P*_1_ and *P*_2_. This is useful for designing regularization terms for keeping the smooth property of neural networks.

### Algorithm of Beaconet

Beaconet contains three components: an optimization objective for measuring the difference among batches, a corrector network for correcting the data of cells in *X* (cell‐by‐gene matrix) by estimating the correction vectors, a collection of auxiliary encode‐L ${\left\{W_{i}\right\}}_{i=1}^{M}$ (neural network, described in Section 5.2.3) for supporting the objective function to measure the batch variations in a differentiable way. Beaconet accepts the collection of log‐scaled expression matrices $X={\left\{X_{i}\right\}}_{i=1}^{M}$ as its input and outputs an integrated expression matrix $\hat{X}={\left[{\hat{X}}_{1}^{T},{\hat{X}}_{2}^{T},\text{…},{\hat{X}}_{M}^{T}\right]}^{T}$, in which each ${\hat{X}}_{i}$ (cell‐by‐gene matrix) is the corrected data for *X_i_*. The training process of Beaconet consists of two major steps (Algorithms S1–S4, Supporting Information). In the first step, the corrector *C* (neural network, described in Section 5.2.2) is disabled and the encoder‐L ${\left\{W_{i}\right\}}_{i=1}^{M}$ is pre‐trained to approximate the batch effect in the original data *X*. In the second step, the corrector *C* and the encoder‐L ${\left\{W_{i}\right\}}_{i=1}^{M}$ are trained together incrementally and iteratively: 1) update *C* with the guidance of the current ${\left\{W_{i}\right\}}_{i=1}^{M}$ and the objective function, 2) update and refine ${\left\{W_{i}\right\}}_{i=1}^{M}$ to approximate the current integrated expression matrix based on the current *C*. During training, the parameters of *C* and ${\left\{W_{i}\right\}}_{i=1}^{M}$ was optimized in mini‐batch manner, in which the cells are sampled from each batch in ${\left\{X_{i}\right\}}_{i=1}^{M}$. Beaconet does not impose any prior bias on the importance of different batches in the network architecture, the training strategy and the objective function. It thus avoids the influence of reference selection. The details of hyperparameters setting are available in Information.

Beaconet was not a simple application of GANs to single‐cell transcriptomic data. There were 3 major differences between Beaconet and standard GANs: 1) Beaconet was designed for data‐correction problems and aims to learn a function that corrects batch‐effect in the given datasets, while GANs were designed to learn a random‐generative model for data from an unknown distribution, 2) GANs deals with two distributions, i.e., the distribution of the training samples and the reference distribution, while the task of Beaconet to integrate data from multiple distributions; and 3) for GANs, the training data were fixed as reference, while in Beaconet, the corrected data of each batch slowly moves.

In the following, on the details of the objective function and the architecture of Corrector *C* and Discriminator *W* (that is the Encoder‐L) in Beaconet was elaborated.

### Objective Function

The objective function of Beaconet is capable of adapting to *M* batches generally. For the intuitive understanding of the objective function, consider the situation where there were only 2 batches of data with expression matrices, *X*_1_ and *X*_2_, and then extend it to a general version for *M* batches. The objective function of Beaconet for two‐batch data (or its payoff function if treated as a zero‐sum game between the corrector C and the encoder‐L *W*) can be written as:

(3) $$\begin{array}{ccc} L & = & E_{x∼P_{2}}\left[W\left(C_{2}\left(x\right)\right)\right]-E_{x∼P_{1}}\left[W\left(C_{1}\left(x\right)\right)\right]\\ & & +\lambda E_{s∼Q}\left[{\left(||\nabla_{s}W\left(s\right)|{|}_{2}-1\right)}^{2}\right] \end{array}$$

In Equation (3), *C*_1_ and *C*_2_ are the Corrector function for cells in batch *1* and batch *2*, To keep the 1‐lipschitz condition, *W* is specified as a fully connective network with specific activate functions such as ReLU and LeakyReLU,^[^ ^19^ ^]^ and the third term is needed. Network *W* is named as encoder‐L for convenience. The aim of Correction function *C* is to minimize the difference of the distributions captured by encoder‐L *W*. The third term in Equation (3) is a penalty, introduced in Section 5.1, for keeping the 1‐lipschitz condition of *W*. Distribution s∼Q is implied by linear interpolation between cells in batch *1* and batch *2*. The default value of the hyperparameter λ is 10, which refers to Gulrajani et. al.^[^ ^60^ ^]^ Then, the optimization objective of 2‐batch version Beaconet is:

(4) $$\begin{array}{ccc} & & \max_{C}\min_{W}E_{x∼P_{2}}\left[W\left(C_{2}\left(x\right)\right)\right]-E_{x∼P_{1}}\left[W\left(C_{1}\left(x\right)\right)\right]\\ & & \;+\lambda E_{s∼Q}\left[{\left(||\nabla_{s}W\left(s\right)|{|}_{2}-1\right)}^{2}\right] \end{array}$$

We next extend the above idea to data with *M* batches. It is assumed that *P_j_* is the distribution of batch *j*, and *P*_{*i*|*i* ≠ *j*}_ is the distribution of the mixture of batch {*i*|*i* ≠ *j*}. Similar to Equation (2) and Equation (3), the Wasserstein distance between *P_j_* and *P*_{*i*|*i* ≠ *j*}_ can be written as Equation (5):

((5) $$\begin{array}{c} L_{w_{j}}=E_{x∼P_{j}}\left[W_{j}\left(C\left(x,j\right)\right)\right]-E_{x∼P_{\left\{i|i\neq j\right\}}}\left[W_{j}\left(C\left(x,i\right)\right)\right]\\ =E_{x∼P_{j}}\left[W_{j}\left(C\left(x,j\right)\right)\right]-\frac{1}{M-1}\sum_{i=1}^{M}I(i\neq j)E_{x∼P_{i}}\left[W_{j}\left(C\left(x,i\right)\right)\right] \end{array}$$

In Equation (5), *C* is the Corrector for correcting the cell ***x*** of batch *i*, *P_i_* is the distribution of batch *i*, *W_j_* is the Discriminator for estimating Wasserstein distance between *P_j_* and *P*_{*i*|*i* ≠ *j*}_. It is noted that Equation (5) as an objective function is able to simultaneously integrate *M* batches but the importance of batch *j* and other batches is asymmetrical. Therefore, the objective function of the Beaconet was defined as the sum of $\left\{L_{w_{j}}|j=1,\text{…},M\right\}$, that is:

(6) $$\begin{array}{c} L_{w}=\sum_{j=1}^{M}\left\{E_{x∼P_{j}}\left[W_{j}\left(C\left(x,j\right)\right)\right]-\frac{1}{M-1}\sum_{i=1}^{M}I(i\neq j)E_{x∼P_{i}}\left[W_{j}\left(C\left(x,i\right)\right)\right]\right\}\\ =\sum_{i=1}^{M}\sum_{j=1}^{M}f\left(i,j\right)E_{x∼P_{i}}\left[W_{j}\left(C\left(x,i\right)\right)\right] \end{array}$$

where the function *f* is defined as follows:

(7) $$f\left(i,j\right)=\left\{\begin{array}{c} 1,\;\;\;i=j,\\ -\frac{1}{M-1},\;i\neq j \end{array}\right.$$

After adding the soft penalty term for 1‐lipschitz condition to *L_w_*, the final form of the objective function of Beaconet is Equation (8):

(8) $$\begin{array}{ccc} \max_{C}\min_{W}L & = & \sum_{i=1}^{M}\sum_{j=1}^{M}\{f\left(i,j\right)\cdot E_{x∼P_{i}}\left[W_{j}\left(C\left(x,i\right)\right)\right]\\ & & +\lambda E_{s∼Q_{i,j}}\left[{\left(||\nabla_{s}W_{j}\left(s\right)|{|}_{2}-1\right)}^{2}\right]\} \end{array}$$

In which *Q*_*i*,*j*_ is implicitly defined by the linear interpolation between the cells in *P_i_* and *P_j_*.

With the objective function described above, Beaconet does not depend on the pre‐selected ordering or individual reference batch and prior knowledge about the content of these datasets. By optimizing Equation (8), the batch effect among given batches will be captured by Discriminator *W* and corrected by Corrector *C* simultaneously in a reference‐free fashion.

### Corrector

The corrector takes the original expression of cells ***x*** (each cell in *X*) as inputs and output the corrected $\hat{x}$ by estimating a correction vector. It consists of three parts linked together sequentially. The first part was a stack of three fully‐connected layers. In this part, while the first two layers use LeakyReLU activation functions, the third layer uses a hyperbolic tangent function as its activation function that does not impose prior knowledge about the direction of the correction vector. The second part was a layer that implements Batch‐Specific Normalization (BS‐Norm), one for each batch, defined as z = *a_i_* · y + b_i_ where the input *y* is the output for the batch *i* from the first part, *z* is the output, *a_i_* and *b*_*i*_ are the scaling factor and bias for the batch *i*. With the inspiration of Trans‐Norm in transfer learning,^[^ ^61^ ^]^ the BS‐Norm reduces the fitting burden of the fully‐connected layers by separating the learning of the parameters unique to individual batches from the learning of those that were common to all batches. It thereby helps improve the effectiveness of Beaconet significantly. The third part is a layer that adds the scaled correction vectors ***z*** to the corresponding expression vector ***x*** followed by a ReLU operation to filter out incorrect values that lead to negative corrected expression features in $\hat{x}$.

### Encoder‐L

There is a collection of encoders ${\left\{W_{i}\right\}}_{i=1}^{M}$ satisfying 1‐lipschitz condition as the auxiliary function for the objective function described above. Each encoder‐L *W_i_* is a stack of three fully‐connected layers to project the features of cells to a real number. The first two layers use LeakyReLU as an activate function and the last layer has no activate function, as this network architecture is beneficial for the holding of *1‐*lipschitz condition.^[^ ^19^, ^60^ ^]^

### Positive Merge Divergence

PMD was a 2‐stage quantitative metric for evaluating integration methods, especially when the datasets have different compositions of cell populations. PMD has two submetric, positive rate and merge divergence. It first identifies cells that belong to the same cell types as their local neighbors as positive cells. The cells overcorrected with other cell types were assigned as negative cells. Second, the mixture degree of batches for positive cells was measured by merge divergence, which was the Jensen‐Shannon (JS) divergence of the proportion of cells in different batches between the local neighborhoods and the global batch proportion of a given cell type. The positive rate indicates the ratio of the cells was not mismatched with other cell types (the larger value shows the purity of cell types was preserved better), and the merge divergence indicates the degree of remaining batch variations of positive cells (zero implies the batch effect was removed).

Given *k* batches of single‐cell datasets, including *m* cell types. The *i‐*th batch has *n*_*i*,*j*_ cell type *j*. *N_p_* is the set of the neighborhoods of cell *p* in *k*NN‐graph, and *c*(*p*) denotes the cell type of cell *p*. In the set *N_p_*, there are ${\hat{n}}_{i,p}$ cells in the batch *i*. In the first stage, PMD identifies the positive cells by check the cells whether surrounded by the same cell type in *k*NN‐graph. In the second stage, PMD calculates the Jensen–Shannon (JS) divergence of ${\hat{f}}_{p}$ and *f*_*c*(*p*)_ for each positive cell *p*.

(9) $${\hat{f}}_{p}=\left(\frac{{\hat{n}}_{1,p}}{\sum_{i=1}^{k}{\hat{n}}_{i,p}},\frac{{\hat{n}}_{2,p}}{\sum_{i=1}^{k}{\hat{n}}_{i,p}},\text{…},\frac{{\hat{n}}_{k,p}}{\sum_{i=1}^{k}{\hat{n}}_{i,p}}\right)$$

(10) $$f_{c(p)}=\left(\frac{n_{1,c(p)}}{\sum_{i=1}^{k}n_{i,c(p)}},\frac{n_{2,c(p)}}{\sum_{i=1}^{k}n_{i,c(p)}},\text{…},\frac{n_{k,c(p)}}{\sum_{i=1}^{k}n_{i,c(p)}}\right)$$

More details of analysis for PMD and existing metrics are available in Supporting Information.

### Experiment Details: Datasets

We collect twelve scRNA‐seq datasets with gold standard cell type labels, including 3 cell line datasets, 2 human blood dendritic datasets, 5 human pancreatic datasets, and 2 mouse atlas datasets. These datasets were widely used in previous studies^[^ ^9^, ^18^, ^23^, ^24^ ^]^ for evaluating integration methods. These three cell line datasets were generated by Zheng, G. et al.,^[^ ^62^ ^]^ and preprocessed by Tran et al.^[^ ^9^ ^]^ for evaluating the integration performance of biological dissimilar monoclonal cell types. Specifically, batch 1 contains only 293T cells (2885 cells), batch 2 contains only Jurkat cells (3258 cells) and batch 3 contains 1605 293T cells and 1783 Jurkat cells. The 2 human blood dendritic (DC) datasets were sorted and analyzed by the same laboratory (Villani et al.)^[^ ^63^ ^]^ using the same sequencing technology (Smart‐Seq2) in 2 batches. These 2 datasets contain CD1C DC, CD141 DC, pDC (plasmacytoid DC), and double negative cells. The CD1C DC in batch 1 and CD1241 DC in batch 2, 2 biological similar cell types, were removed in a previous benchmark study^[^ ^9^ ^]^ for constructing a well‐controlled benchmark task for overcorrection. The integration of these two DC datasets was characterized by the small batch variance and biologically similar batch‐specific cell types. The challenge of the integration of the datasets was that the batch‐effect removal methods must preserve the difference in the batch‐specific cell types (CD141 DC and CD1C DC) while mixing the batch‐shared cell types of 2 batches well (pDC and double negative cell). The 5 human pancreatic datasets were generated in 5 different studies^[^ ^36^, ^37^, ^38^, ^39^, ^40^ ^]^ with accession codes GSE84133, GSE85241, GSE83139, GSE 81608, and E‐MTAB‐5061, respectively. These datasets were different in experimental laboratories, sequencing protocols, and samples, and varied in the number of cell types, the proportion of cell types, and the number of cells. The sequencing technologies include CelSeq2, SMART‐seq2, inDrops and SMARTer. The number of possible references and merge orderings is 5+5! = 125 at most, which may derive different integration results. The preprocessed data for these pancreatic datasets from a benchmark study was downloaded.^[^ ^9^ ^]^ The Marker genes for human pancreatic tissue were downloaded from CellMarker database.^[^ ^45^ ^]^ For the mouse atlas integration, The original data was generated by Tabular muris,^[^ ^3^ ^]^ in which the gene expression profiles of cells were generated by two distinct technical approaches, droplet (microfluidic droplet‐based 3’‐end counting) and FACS (full‐length transcript analysis based on fluorescence‐activated cell sorting). We download the data in loom files from a previous study.^[^ ^18^ ^]^ These two datasets contain the single‐cell transcriptomic data of more than 100 000 cells from 19 tissues of the mouse. The two datasets to evaluate the efficiency of Beaconetb were used. In the ablation experiments for demonstrating the necessity of BS‐Norm for Beaconet, besides the integration tasks described above,7 tissues from the 19 tissues in Tabula Muris were selected for assessment, including marrow, limb_muscle, trachea, spleen, tongue, lung, and mammary_gland. These tissues were selected by three criteria: 1) The selected tissue should contain cells in both 2 batches; 2) The selected tissue should contain more than one cell type; 3) Selecting the tissues with more cells.

### Compared Methods

We selected 13 representative unsupervised batch‐effect removal methods, including Harmony,^[^ ^23^ ^]^ Seurat,^[^ ^29^ ^]^ MNNCorrect,^[^ ^17^ ^]^ iMAP,^[^ ^18^ ^]^ RPCI,^[^ ^22^ ^]^ FastMNN (https://marionilab.github.io/FurtherMNN2018/theory/description.html),^[^ ^17^ ^]^ LIGER,^[^ ^30^ ^]^ FIRM,^[^ ^31^ ^]^ BBKNN,^[^ ^32^ ^]^ scVI,^[^ ^33^ ^]^ scDML,^[^ ^26^ ^]^ DESC,^[^ ^25^ ^]^ and Scanorama^[^ ^24^ ^]^ to assess the performance of Beaconet. The chosen methods covered a wide range of methodology, including deep learning‐based (iMAP, DESC, scVI, scDML), matrix decomposition‐based (RPCI, LIGER), graph‐based (Scanorama, BBKNN), cluster‐based (Harmony, FIRM) and mutual nearest neighbor pairs matching‐based (MNNCorrect, FastMNN, Seurat) methods. The output space of the result of these methods were different. Scanorama, Seurat, MNNCorrect and iMAP correct batch effect in the original gene expression space. Harmony, RPCI, FastMNN, scVI, scDML, LIGER and DESC integrate the batches in the low‐dimensional embedding space. The dimensions the embedding space of RPCI was ordered by the variance of principle component. The output form of BBKNN was graph of cells. For the dependence of reference, they were divided into two types. The first was based on the reference orderings (i.e., merge orderings), in which the different order of data matrix may derive different result. MNNCorrect, iMAP and FastMNN, FIRM were based on the reference ordering. The second type was based on an individual reference dataset. The different batch selected as reference dataset will derive different result. Harmony, Seurat, RPCI, LIGER and iMAP were based on reference batches. In particular, iMAP could receive either a reference sequence or a reference dataset. For the methods that need no reference, Scanorama, DESC, scVI, scDML, and BBKNN were benchmarked. Only Scanorama and the presented Beaconet have both properties of original feature space and reference‐free. “Uncorrected” was also used to indicate the data without processing by any methods as a baseline method. The dimension number of the corrected results of these methods varies from 30 to 2000. As the scale of distance varies in different spaces, all integration metrics after reducing the dimensions of all results into the same number by UMAP to avoid the potential bias of metrics for different scaling of space were calculated. Any parameters for specific datasets for all methods were not fine‐tuned. The *k*‐means clustering was used before calculating ARI and NMI. The version of all packages is provided in Tables S24–S26. The hyper‐parameters of all methods and functions in the experiments in this study are provided in Table S27. To explore the potential best performance of compared methods, all possible references were applied. For the reference ordering (merge ordering) based methods, all possible combination orderings were applied. If *m* batches of datasets required integration, the number of orderings was *m*!, that was *m* × (*m* − 1) × (*m* − 2) × … × 1. For individual reference‐based methods, all possible reference batches were applied. The number of possible reference batches was equal to the number of batches *m*. If the integration method accepts merge ordering or reference batch, it was repeated *m*! + *m* times. Specifically, Beaconet, DESC, Scanorama, scVI, scDML, BBKNN only need one run for each task since they require no reference when integration. RPCI, Seurat, Harmony were run two times on two‐batch DC datasets, three times on three‐batch cell line datasets, and five times on five‐batch human pancreatic datasets. FastMNN and MNNCorrect were run two times on 2‐batch DC datasets, 6 times on 3‐batch cell line datasets, and 120 times on 5‐batch human pancreatic datasets. iMAP could handle both ordering and reference, thus, it was run two times on two‐batch DC datasets (for two‐batch integration, merge orderings “1_2” and “2_1” are equivalent to using batch 1 as a reference to integrate batch 2 and using batch 2 as a reference to integrate batch 1), 9 (9 = 3! + 3) times on three‐batch cell line datasets, 125 (125 = 5! + 5) times on five‐batch human pancreatic datasets. FIRM was not applied on human pancreatic datasets due to its high computational complexity.

### Data Availability Statement 

These three cell line datasets are generated by Zheng, G. et al.^[^ ^62^ ^]^ The two human blood dendritic (DC) datasets were sorted and analyzed by Villani et al.^[^ ^63^ ^]^ using Smart‐Seq2. The five human pancreatic datasets were generated in 5 different studies^[^ ^36^, ^37^, ^38^, ^39^, ^40^ ^]^ with accession codes GSE84133, GSE85241, GSE83139, GSE 81608, and E‐MTAB‐5061, respectively. The processed data of the above datasets was downloaded from this published article: Tran HTN, Ang KS, Chevrier M, et al. A benchmark of batch‐effect correction methods for single‐cell RNA sequencing data. Genome Biol. 2020;21(1):12. doi:10.1186/s13059‐019‐1850‐9.^[^ ^9^ ^]^ The two datasets in Tabula Muris were generated by this published article: Tabula Muris Consortium, et al. Single‐cell transcriptomic of 20 mouse organs creates a Tabula Muris. Nature. 2018;562(7727):367‐372. doi:10.1038/s41586‐018‐0590‐4,^[^ ^3^ ^]^ and the data was downloaded in the format of a loom file provided by: Wang D, Hou S, Zhang L, Wang X, Liu B, Zhang Z. iMAP: integration of multiple single‐cell datasets by adversarial paired transfer networks. Genome Biol. 2021;22(1):63. doi:10.1186/s13059‐021‐02280‐8 in the web site: https://cloud.tsinghua.edu.cn/f/9480867d1223425988a4/?dl=1. The marker genes of human pancreatic tissue analyzed during this study were derived from this database: Xinxin Zhang, Yujia Lan, Jinyuan Xu, Fei Quan. CellMarker database. Accessed June 7, 2022. http://xteam.xbio.top/CellMarker/. The marker gene, PPY, of gamma cells in human pancreatic tissue is included in this published paper: Baron M, Veres A, Wolock SL, et al. A Single‐Cell Transcriptomic Map of the Human and Mouse Pancreas Reveals Inter‐ and Intra‐cell Population Structure. Cell Syst. 2016;3(4):346‐360.e4. doi:https://doi.org/10.1016/j.cels.2016.08.011. Beaconet and the Positive Merge Divergence metric were available as a Python package on GitHub (https://github.com/GaoLabXDU/Beaconet). All code scripts, and preprocessed data of our experiments were freely accessible on figshare (https://doi.org/10.6084/m9.figshare.20764843) to ensure reproducibility.

### Statistical Analysis: Pre‐Processing of Data

For all integration tasks in this work, the obtained datasets using the package SCANPY were reprocessed.^[^ ^46^ ^]^ First, “scanpy.pp.filter_genes” was used to filter the genes that were expressed in at most 3 cells. Second, “scanpy.pp.filter_cells” was used to remove the cells with less than 300 expressed genes as outliers. Since the sequencing depth was different in each cell, the gene expression for library size by “scanpy.pp.normalize_total” was normalized, and then transformed the raw counts into log‐scaled counts. the top 2000 highly variable genes were selected as the most informative features by “scanpy.pp.highly_variable_genes”. For the purpose of a fair comparison of these batch‐effect removal methods, the same input files were used for all methods. The processed datasets and the code scripts are provided in figshare (https://doi.org/10.6084/m9.figshare.20764843) to ensure reproducibility.

### Sample Size for Each Statistical Analysis

For the t‐test of the expression of gene PPY between gamma cells and other cells, there were 656 gamma cells and 14 111 other cells. For the t‐test of expression of CELA2A and RNASE1 among two subpopulations of ductal cells and acinar cells, there were 899 and 178 cells in the two subpopulations, and 958 acinar cells. For the ablation study, there were 14 767 cells in human pancreatic datasets, 565 cells in human blood dendritic cell datasets, 9531 cells in cell line datasets, and 100 605 cells in Tabula Muris. For the t‐test comparing the distribution of merge divergence scores of positive cells in human pancreatic datasets, there were 14 767 cells.

### Data Presentation

For the comparison of Beaconet and baseline methods, the reference‐based methods were applied with all possible reference batches. In each run, the positive rate and median value of merge divergence of cells were the indications for evaluating performance, which are called PMD scores. In Figure 3B and Figure 4D, the results of these methods were shown by the average PMD scores and the max/min scores of all runs with different references, in which the max/min positive rate and merge divergence were reported using error bars in horizontal and vertical directions. The error bars indicated the uncertainty caused by different reference selections, rather than the difference of performance among tasks. For the differential expression analysis, the expression values using a boxplot were shown, including the 25th, 50th, and 75th percentiles. For the ablation study, the merge divergence scores of cells were also shown using a boxplot.

### Statistical Methods

For differential expression analysis, the function ‘scanty.tl.rank_genes_groups’ was applied in SCANPY,^[^ ^46^ ^]^ in which genes were ranked by a score based on the adjusted *p*‐values of Wilcoxon rank‐sum test and logFC for each cell type. For the comparison of the subpopulations of ductal cells and acinar cells, a 2‐sided t‐test was applied. To evaluate whether the BS‐Norm module improves the capacity of Beaconet, a 1‐sided rank‐sum test and a 1‐sided permutation test were applied. To test whether Beaconet and the variant of Beaconet have no significantly different performance on the integration of human blood dendritic cell (DC) datasets, a 2‐sided rank‐sum test was applied, 2‐sided permutation test, and 1‐sided permutation test. For the permutation test, 9999 times for permutation were repeated. Assuming the observed 2 groups of samples are X and Y. In each permutation, the samples were randomly separated into 2 groups (A and B), and the mean values mean(A) and mean(B) were calculated separately. The absolute difference value of the two mean values, abs(mean(A)‐mean(B)), were used to compare with the observed absolute difference value in real groups, abs(mean(X)‐mean(Y)), for the 2‐sided test. The mean(A)‐mean(B) and mean(X)‐mean(Y) is used for a 1‐sided permutation test with the option “larger”, and mean(B)‐ mean(A) and mean(Y)‐ mean(X) is used for 1‐sided permutation test option “smaller”.

### Software for Statistical Analysis

For the differential expression genes identification, function ‘scanpy.tl.rank_genes_groups’ was used in version 1.7.1 of SCANPY,^[^ ^46^ ^]^ in which genes were ranked by a score based on the adjusted *p*‐values of Wilcoxon rank‐sum test and logFC for each cell type. For the 2‐sided t‐test in Figure 5C, ‘scipy.stats.ttest_ind’ function in version 1.5.4 of the Scipy package was used. A permutation test using 1.19.5 version of the numpy package was implemented, the code script is available at https://doi.org/10.6084/m9.figshare.20764843. For two‐sided and one‐sided rank‐sum tests in the ablation study, ‘scipy.stats.ranksums’ function in 1.10.1 version of Scipy package on Python3.10 was used. It is because that version 1.5.4 of Scipy on Python3.6 cannot support 1‐sided rank‐sum test.

## Conflict of Interest

The authors declare no conflict of interest.

## Author Contributions

H.X. performed conceptualization, data curation, formal analysis, investigation, methodology, validation, visualization, and software. H.X., Y.Y., Y.G., Y.H., L.G., and R.D. wrote, reviewed, and edited the original draft. Y.G. performed funding acquisition. Y.H. performed Conceptualization, Funding Acquisition, Visualization, and Validation. L.G. performed Conceptualization, Funding Acquisition, Project Administration, Resources, and Supervision.

## Supporting information

Supporting Information

Supporting Information

Supporting Information

Supporting Information

Supporting Information

Supporting Information

## Data Availability

The data that support the findings of this study are openly available in figshare at https://doi.org/10.6084/m9.figshare.20764843.
