# Supplementary material for: Beaconet: A Reference‐Free Method for Integrating Multiple Batches of Single‐Cell Transcriptomic Data in Original Molecular Space
Source: Adv Sci (Weinh). 2024 May 6;11(26):2306770. doi: 10.1002/advs.202306770 (PMC11234410; doi:10.1002/advs.202306770)
Supplement: Supplementary file 1 — Supporting Information [file ADVS-11-2306770-s004.pdf]

ADVANCED SCIENCE
Open Access

Supporting Information

for *Adv. Sci.*, DOI 10.1002/advs.202306770

Beaconet: A Reference-Free Method for Integrating Multiple Batches of Single-Cell Transcriptomic Data in Original Molecular Space

Han Xu, Yusen Ye, Ran Duan, Yong Gao, Yuxuan Hu and Lin Gao**

Supplementary Information

Contents

Supplementary Method 1

Supplementary Tables 8

Supplementary Figures 42

Reference 75

Supplementary Method

The implementation details of Beaconet

In this section, we describe our implementation of Beaconet in details. This implementation of Beaconet consists of a corrector, a collection of encoder-L, an objective function, the optimizer and the training strategy. The corrector network is shared by all cells from each batch. There are M independent encoder-L with the same architecture to cooperate with the objective function to indicate the difference of the distributions of multiple batches based on the Wasserstein distance. The *i*-th encoder-L is to detect the difference between the distributions of batch *i* and the joint distribution of other batches. The training strategy allows the parameters of corrector and encoder-L update cooperatively.

We first introduce the forward computation process in corrector. The corrector c takes a mini-batch with s samples (the default value is 1024) of the RNA-seq vectors x of cells and the corresponding batch index b as input (the meaning of the term "batch" in "batch index" is corresponding to "batch effect", and the "mini-batch" is corresponding to "mini-batch gradient descent"). We use an encoder with three fully-connected layers to transform the RNA-seq features to the unscaled correction vectors of cells. The number of the neural units in the encoder is d , d and p , in which p is equals to the number

of highly variable genes of cells in datasets (the default value is 2000) and d is the dimension of the latent space in encoder (the default value is 256). They map the number of dimension of vectors to change in the following sequence: $2000 \rightarrow 256 \rightarrow 256 \rightarrow 2000$. The activate function in the first two layers is LeakyReLU with 0.1 negative slope, which is benefit to the gradient flow in the network. The activate function of the third layer is the hyperbolic tangent function, which avoids to impose prior knowledge about the direction of the correction vectors. After getting the unscaled correction vectors, we use the BS-Norm module introduced in "**Error! Reference source not found.**" section to modify the correction vectors by the batch-specific scale and bias, which is learnable factor for each batch. And then we add the scaled correction vectors and the RNA-seq data, and filter the non-positive corrected values by ReLU function.

Each encoder-L W is a three fully-connected network. The activate function of the first two layers is LeakyReLU function, while the final layer have no activate function. The reason of that is the aim of the network W is not to distinguish the batch categories of the input cells but to help the objective function to regress the Wasserstein distance between the distributions. The number of the dimension of latent space in the encoder-L is d. Each encoder-L takes a mini-batch of the RNA-seq data X as input, and transforms each vector to a real number $w = W(x)$. The Wasserstein-*k* distance between the distribution of batch i and the joint distribution of other batches is $\frac{1}{n}\sum_{x \in batch_i} w_i(x) - \frac{1}{(m-1)\times n}\sum_{x \notin batch_i} w_i(x)$ (in this computational manner, the *k* of Wasserstein-*k* distance is usually unknown, therefore the specific value of the distance in objective function is meaningless, but it is still effective to guide the update of parameters in neural network, since the optimizer only need the direction of gradient but ignore the constant scale term *k* [1]). The LeakyReLU activate function in network and the penalty term of objective function could guaranteed constrained condition [1,2].

The training strategy consist of two stage: in the first stage, we disable the corrector and pre-training the encoer-L by the uncorrected single-cell transcriptomic data; in the second stage, the corrector and encoder-L are trained in the adversarial way. The aim of encoder-L is to maximize the objective function to lead the Wasserstein distance more accurate, while the aim of corrector is to modify the RNA-seq data by correction vector to remove the batch effect. The default value of the hyper-parameter λ for holding

1-lipschitz is 10. We use Adam [3] to optimize the objective function to update the parameters in Beaconet and the betas in the optimizer were set to 0 and 0.999. It is important for setting β_1 to 0. The reason of this setting of optimizer is to avoid the potentially non-stationary problem with the distribution of data changing, as reported in [1]. The learning rate is set as 0.0002, which is recommended in WGAN. As the objective function of neural network is the non-convex, Beaconet may get better performance for specific datasets by tune the hyper-parameters. However, we did not fine-turn any hyper-parameters for specific datasets to emphasis the utility of Beaconet in practice. In our experiments, the hyper-parameters were fixed as default values in all datasets, which differ in the volume of cells, the diversity of cell types, the number of batches and the sequencing technologies. We provided the Python implementation of Beaconet at GitHub (https://github.com/GaoLabXDU/Beaconet) [4]. The experimental environment of this study is Python 3.6.8 on Windows 10, Intel(R) Core (TM) i7-9700F. The memory size is 16 GB. The version of CUDA is 10.1.105. The GPU is NVDIA GeForce GTX 1660Ti with 6 GB memory.

For efficiently deal with large-scale datasets in gene-expression space, the idea of Beaconet is to convert the matching of cell pairs to the alignment of the distribution of batches with inspiration of iMAP [5], and then approximates a universal correction function for cells in all batches. During training, Beaconet is optimized by mini-batch stochastic gradient descent methods in a pre-estimated number of epochs, rather than traversing the whole datasets in each step of the optimization processing. After training, the correction function is able to integrate the cells in the different batches of data within linear time complexity. Thus, the computational burden of Beaconet is greatly relaxed including time and memory cost.

Metrics

PMD (Positive Merge Divergence). The composition of cell populations varied across different batches of datasets [5–7]. The cell types without counterpart cell groups in other batches may be overcorrected to merge with other cell types when removing batch effect among datasets. Therefore, besides developing accurate batch effect removal methods, it is also important for fairly evaluating the performance of batch effect removal methods to consider the differences in cell composition among different batches of

datasets. Existing quantitative evaluation metrics for batch effect removal can be categorized into two types [5]: cluster-level metric (e.g., ARI, NMI) and individual cell-level metric (e.g., kBET [8], LISI [9]). Cluster-level metrics were able to measure the batch effect influencing the structure of clusters, but ignore the batch effect of cells within the same cluster. Individual cell-level metrics were useful to capture batch effect at cell level. However, state-of-the-art batch effect removal metrics kBET [8], LISI [9] and their variants in previous studies [10,11] may be bias to the overcorrected results due to insufficient consideration of batch-specific cell types. We propose a novel metric PMD to overcome the disadvantage of existing metrics for datasets with batch-specific cell types. PMD measures the batch effect using positive rate for detecting and filtering the overcorrected cells and merge divergence for capturing the mixture performance of batches for positive cells. The design of PMD is inspired by the evaluating strategy in previous study [5]. We changed a hard threshold for identification of true positive cells to a flexible continuous score based on JS (Jensen-Shannon) divergence. The PMD metric presented in this paper is a two-stage metric, which is adapt at evaluating the integration performance on datasets with different composition of cell populations, especially when batch-specific cell types exist. PMD measures batch effect using two sub-metric positive rate and merge divergence. It first identifies cells that belong to the same cell types as their local neighbors as positive cells. The cells overcorrected with other cell types are assigned as negative cells. Secondly, PMD measures the mixture performance of batches for positive cells by merge divergence, which is the Jensen-Shannon (JS) divergence of the proportion of cells in different batches between the local neighborhoods and the global allocation of the cell type. The positive rate indicates the ratio of the cells are not mismatched with other cell types (the larger value shows the purity of cell types is preserved better), and the merge divergence indicate the degree of remained batch variations of positive cells (zero implies the batch effect is removed).

ARI (Adjusted Rand Index) [12]. This metric was originally used to measure the performance of clustering algorithms by measuring the consensus of the clustering labels and the ground truth labels of cell types. It first calculate the raw RI (Rand Index) to measure the agreement of the clustering label assignment and the ground truth by the counts of each pair of labels. Secondly, adjust the RI score to account for the possibility of the agreement occurs by chance. The value range of this metric is between -

1 and 1, and the larger score indicates the better clustering performance. Many recent studies [5,10,11,13,14] of batch-effect removal methods for multiple batches of single-cell datasets have used the ARI for measure the preservation of the clusters of cell types on the integrated data. The assumption behind this metric is that batch effect can affect clustering performance of integrated data. That is to say, the well-integrated datasets should be assigned higher ARI scores. However, since this metric is measured at the cluster-level, it cannot reflect the differences in batch effect among different integration datasets if the batch effect is not severe enough to affect the structure of the cluster, even if the batch effect could be observed clearly within the cluster.

NMI (Normalized Mutual Information) [15]. This metric was also originally used to measure the performance of clustering algorithms by calculating the mutual information of the clustering labels and the ground truth labels of cell types and adjusting the mutual information score by normalization coefficient. The value range of this metric is between 0 and 1, and the larger score indicates the better clustering performance. The limitation of this metric for evaluating batch effect is similar to ARI.

kBET [8]. kBET is a hypothesis testing-based metric for batch effect. It measures the batch effect for each cell and then uses median scores of all cells for reflecting the ability of batch-effect removal methods to correct the batch effect. For a given cell, we suppose that, in the *k*-neighbors set, $b_1, b_2, ..., b_M$ are the number of cells coming from batch $1, 2, ..., M$. We suppose the number of cells in different batches are $B_1, B_2, ..., B_M$ respectively. The null hypothesis of kBET is that the proportion of $b_1, b_2, ..., b_M$ is approximately equal to the proportion of $B_1, B_2, ..., B_M$. kBET calculates accept rates (or reject rates; reject rates are equal to one minus accept rates) based on the results of hypothesis testing for reflecting the batch effect remaining in the integrated data. The higher accept rates indicate that the datasets are better integrated. The limitation of kBET is that it ignores the difference of the proportion of each cell type in different batches, and thus assumes the optimal merging proportion of batches in the local neighbors of each cell is identical, which was also reported in [9]. Besides directly calculating kBET

on integrated dataset, the existing studies have calculated kBET in other two strategies for reducing the impact of the limitation. The first strategy [11] is to calculate for cells of each cell type separately ($B_1, B_2, ..., B_M$ are also been recalculated for each cell type), and then average the kBET scores for cell types. The limitation of this strategy is that the overcorrected cells are not reflected in the result of metric, since all cell types are separated when evaluating kBET score. The other limitation is that it can only by applied on the cell types existing in at least 2 batches, and thus, the batch-specific cell types are filtered out. The second strategy [10] is to down sample cells to ensure the proportions of each cell type in different batches is identical. In this way, the optimal merging proportion of each cell with $b_1, b_2, ..., b_M$ neighbors is the proportion of $B_1, B_2, ..., B_M$. We suppose $n_{i,j}$ is the number of cells with cell type i in batch j. After down sampling, $\hat{n}_{i,j} = \min_{k=1,2...,M} n_{i,k}$. The limitation of this strategy is that it can only capture the batch effect for the cell types existing in all batches since the cell types that do not exist in at least one batch of data are filtered out. We calculated kBET in the three strategies in this study (kBET_1, kBET_2 and kBET_3, respectively).

LISI (Local Inverse Simpson Index) [9]. LISI is a Simpson index-based metric. It measures the performance of integrated data in two aspect, iLISI for the mixture of batches and cLISI for the purity of cell types. Without loss of generality, we take iLISI as an example. We suppose the fractions of cells from each batch are $p_1, p_2, ..., p_M$ in the local k-neighbors of a given cell. The value of iLISI is calculated as

$$iLISI = \frac{1}{\sum_{i=1}^{M} p_i^2}.$$

The iLISI scores reflect the diversity of batches in the local neighbor set of given cells. The maximum value M can be reached when all p_i is identical for $i = 1, 2, ..., M$ and the minimum value 1 can be reached when $\exists i\ \forall \mathrm{j} \neq \mathrm{i} : p_i = 1,\ p_j = 0$. It is thus expected that the better integrated data will be assigned larger iLISI and smaller cLISI. There are two limitations in LISI metric. The first is that,

although the mismatched cells can be captured by higher cLISI, these cells still are involved for evaluating the performance of mixture of batches in iLISI. It results in the overcorrected data may get better mixture score of batches (iLISI) by slightly abating the purity of cell types (cLISI), and thus shows that the overcorrected data and well-integrated data is comparable. The second is that it only considers the local probability p_i from different batches in numerical terms, but ignores the matching relation of the global probability and local probability of each batches, which may lead to an equal score for different mixing performance of batches.

Averaged PMD metrics across several integration tasks. Suppose there are T integration tasks. In the task t, there are M_t batches of datasets. N represents the number of integration methods, and the integration method i have $r_{i,t}$ different integration results on task t. The PMD scores on different integration tasks may have different numerical scales, since the difficulty of each task is different and the number of batches in each task is also not identical. We must remove the difference of numerical scales of different integration tasks by z-score transformation. As the number of possible integration results varies in different methods, we consider each method has equal contribution for the statistics of mean value and variance. Suppose $pr_{i,j,t}$ and $md_{i,j,t}$ are the positive rate and merge divergence of j-th integration result of method i on task t. For integration task t, we have

$$\mu_{pr,t} = \frac{1}{N}\sum_{i=1}^{N}\frac{1}{r_{i,t}}\sum_{j=1}^{r_{i,t}} pr_{i,j,t}$$

$$\sigma^2_{pr,t} = \frac{1}{N-1}\sum_{i=1}^{N}(\frac{1}{r_{i,t}}\sum_{j=1}^{r_{i,t}} pr_{i,j,t} - \mu_{pr,t})$$

$$\mu_{md,t} = \frac{1}{N}\sum_{i=1}^{N}\frac{1}{r_{i,t}}\sum_{j=1}^{r_{i,t}} md_{i,j,t}$$

$$\sigma^2_{md,t} = \frac{1}{N-1}\sum_{i=1}^{N}(\frac{1}{r_{i,t}}\sum_{j=1}^{r_{i,t}} md_{i,j,t} - \mu_{md,t})$$

And then we can easily get the z-score scaled positive rate and merge divergence for each task.

$$z\_pr_{i,j,t} = \frac{pr_{i,j,t} - \mu_{pr,t}}{\sigma_{pr,t}}$$

$$z\_md_{i,j,t} = \frac{md_{i,j,t} - \mu_{md,t}}{\sigma_{md,t}}$$

After z-score, we respectively average the mean, maximum and minimum positive rate and merge divergence across T integration tasks for each methods. Finally, the overall performance on several integration tasks can be drawn be scatter plot with error bar, in which the error bar indicates the overall uncertainty caused by the selection of reference or merge orderings. The intermediate tables for summarizing the overall performance evaluation is available in Additional file 7.

Supplementary Tables

Algorithm S1. The overview of Beaconet.

Algorithm S2. The initialization of the module in Beaconet.

Algorithm S3. The forward propagation of correction process.

Algorithm S4. The strategy to update the parameters of Beaconet during training.

Table S1. Evaluating the 45 integrated output of the compared methods and Beaconet using 9 metrics on the three batches of cell line datasets.

Table S2. Evaluating the 22 integrated data of the compared methods and Beaconet using 9 metrics on two batches of DC datasets.

Note: We provide the table of the metrics for five human pancreatic datasets in Supplementary file 3: Table S1. (xlsx). It has 392 rows as the diversity of the selection of reference for the compared methods.

Table S3. The top-10 differentially expressed genes of alpha cells.

Table S4. The top-10 differentially expressed genes of ductal cells.

Table S5. The top-10 differentially expressed genes of gamma cells.

Table S6. The top-10 differentially expressed genes of acinar cells.

Table S7. The top-10 differentially expressed genes of beta cells.

Table S8. The top-10 differentially expressed genes of delta cells.

Table S9. The top-10 differentially expressed genes of endothelial cells.

Table S10. The top-10 differentially expressed genes of macrophage cells.

Table S11. The top-10 differentially expressed genes of mast cells.

Table S12. The top-10 differentially expressed genes of mesenchymal cells.

Table S13. The top-10 differentially expressed genes of MHC class II cells.

Table S14. The top-10 differentially expressed genes of epsilon cells.

Table S15. The top-10 differentially expressed genes of schwann cells.

Table S16. The top-10 differentially expressed genes of stellate cells.

Table S17. The top-10 differentially expressed genes of T cells.

Table S18. The positive rates of the full Beaconet and the variant Beaconet without BS-Norm on two-batch DC datasets, five-batch human pancreatic datasets, three-batch cell line datasets, two-batch Tabula muris datasets, limb_muscle, lung, mammary_gland, marrow, spleen, tongue, trachea.

Table S19. The one-sided permutation test P values for determining whether BS-Norm improves the performance of Beaconet.

Note: We selected one-sided rather than two-sided permutation test, since what we are concerned about is whether the merge divergence significantly decreased by applying BS-Norm for Beaconet, rather than whether there were significant difference between the full Beaconet (using BS-Norm) and the variant Beaconet (disabled BS-Norm). For DC datasets, we perform two-sided permutation test, besides the one-sided permutation test, since we would like to demonstrate Beaconet did not performed better or worse by applying BS-Norm on the integrated DC datasets. In fact, on DC datasets, both of the P values of one-sided test and two-sided test are not significant.

For constructing the empirical distribution in permutation test, we repeated 9999 times permutation for each pair of comparison.

Table S20. The rank-sum test P values for determining whether BS-Norm improves the performance of Beaconet.

Table S21. An example of the cell numbers with each cell type and batch.

Table S22. The local neighbors' allocation of a well corrected cell with cell type 1 in an example.

Table S23. The local neighbors' allocation of an overcorrected cell with cell type 1 in an example.

Table S24. The detail information of experimental platform.

Table S25. The R packages and the version number used in our study.

Table S26. The Python packages and the version number used in our study.

Table S27. The hyper-parameters of all methods and functions used in our study.

Algorithm 1 Beaconet

Function BEACONET ($X_1, X_2, ..., X_n, C, W$)

$W \leftarrow$ INITIALIZATION($X_1, ..., X_n$,W)

Repeat

Optimize C by minimizing Eq.**Error! Reference source not found.** in one step using Adam

$W \leftarrow$ UPDATE($X_1, ..., X_n, C, W$)

Until convergence

$\hat{X}_1, ..., \hat{X}_n \leftarrow$ CORRECT($X_1, ..., X_n, C, W$)

$\hat{X} \leftarrow [\hat{X}_1^T; \hat{X}_2^T; ...; \hat{X}_M^T]^T$

Return $\hat{X}$

Algorithm 2. Initialization

Function INITIALIZATION ($X_1,...,X_M,W$)

For i **from** 1 **to** M //disable the Corrector C

$\hat{X}_i \leftarrow X_i$

Optimize W by maximizing Eq.**Error! Reference source not found.**

Return W

Algorithm 3. Correct

Function CORRECT ($X_1,...,X_M,C$)

For i **from** 1 **to** M

$\hat{X}_i \leftarrow C(X_i,i)$

Return $\hat{X}_1,...,\hat{X}_M$

Algorithm 4. Update

Function UPDATE ($X_1,...,X_M,C,W$)

For i **from** 1 **to** n_critic

$\hat{X}_1,...,\hat{X}_n \leftarrow \text{CORRECT}(X_1,...,X_n,C,W)$

Optimize W by maximizing Eq.**Error! Reference source not found.** in one step using Adam

Return W

Table S1. Evaluating the 45 integrated output of the compared methods and Beaconet using 9 metrics on the three batches of cell line datasets.

	NMI	ARI	iLISI	cLISI	Positive rate	Merge divergence	kBET_1(accept rate)	kBET_2(accept rate)	kBET_3 (accept rate)
Seurat_0	0.204	0.092	2.288	1.672	0.724	0.148	0	0.437	NA
Seurat_1	0.214	0.139	2.01	1.448	0.784	0.118	0	0.311	NA
Seurat_2	0.957	0.981	1.823	1	0.995	0.001	0	0.907	NA
Harmony_0	0.925	0.962	1.831	1	0.995	0.001	0	0.942	NA
Harmony_1	0.906	0.949	1.83	1	0.993	0.001	0.004	0.907	NA
Harmony_2	0.926	0.962	1.838	1	0.995	0.001	0	0.942	NA
iMap_0	0.196	0.09	1.418	1.021	0.938	0.109	0	0.236	NA
iMap_1	0.237	0.151	1.384	1	0.979	0.049	0	0.14	NA
iMap_2	0.964	0.985	1.813	1	0.996	0.002	0	0.324	NA
RPCI_0	0.889	0.941	1.656	1	0.99	0.007	0	0.541	NA
RPCI_1	0.892	0.942	1.713	1	0.99	0.006	0	0.431	NA
RPCI_2	0.919	0.958	1.732	1	0.994	0.004	0	0.438	NA
LIGER_1	0.208	0.087	2.333	1.695	0.726	0.093	0.001	0.093	NA
LIGER_2	0.333	0.275	2.231	1.407	0.789	0.117	0	0.379	NA
LIGER_3	0.307	0.347	2.000	1.314	0.810	0.064	0	0.210	NA
FIRM_1_2_3	0.950	0.977	1.000	1	0.994	0.141	0	0.088	NA
FIRM_1_3_2	0.448	0.391	1.000	1	0.997	0.141	0	0.270	NA

FIRM_2_1_3	0.950	0.977	1.000	1	0.994	0.141	0	0.084	NA
FIRM_2_3_1	0.469	0.439	1.000	1	0.998	0.141	0	0.174	NA
FIRM_3_1_2	0.448	0.391	1.000	1	0.997	0.141	0	0.348	NA
FIRM_3_2_1	0.469	0.439	1.002	1	0.998	0.141	0	0.162	NA
FastMNN_1_2_3	0.003	0.005	1.885	1.145	0.862	0.078	0.024	0.317	NA
FastMNN_1_3_2	0.964	0.985	1.787	1	0.996	0.002	0	0.472	NA
FastMNN_2_1_3	0.037	0.053	1.946	1.096	0.885	0.054	0.008	0.402	NA
FastMNN_2_3_1	0.956	0.981	1.767	1	0.995	0.004	0	0.416	NA
FastMNN_3_1_2	0.965	0.985	1.792	1	0.996	0.002	0	0.594	NA
FastMNN_3_2_1	0.928	0.963	1.779	1	0.996	0.002	0	0.605	NA
MNNCorrect_1_2_3	0.195	0.09	1.303	1.006	0.962	0.104	0	0.314	NA
MNNCorrect_1_3_2	0.966	0.986	1.813	1	0.996	0.002	0	0.574	NA
MNNCorrect_2_1_3	0.232	0.147	1.129	1	0.981	0.084	0	0.166	NA
MNNCorrect_2_3_1	0.958	0.982	1.801	1	0.995	0.005	0	0.323	NA
MNNCorrect_3_1_2	0.964	0.985	1.792	1	0.996	0.002	0	0.484	NA
MNNCorrect_3_2_1	0.96	0.982	1.783	1	0.996	0.005	0	0.569	NA
iMAP_0_1_2	0.065	0.086	1.95	1.307	0.817	0.087	0.004	0.234	NA
iMAP_0_2_1	0.97	0.988	1.802	1	0.997	0.002	0	0.554	NA
iMAP_1_0_2	0.164	0.211	1.757	1.144	0.882	0.066	0.003	0.247	NA

iMAP_1_2_0	0.925	0.961	1.811	1	0.996	0.002	0	0.527	NA
iMAP_2_1_0	0.927	0.963	1.794	1	0.996	0.002	0	0.438	NA
iMAP_2_0_1	0.963	0.985	1.799	1	0.996	0.002	0	0.413	NA
**Beaconet**	**0.949**	**0.977**	**1.821**	**1**	**0.994**	**0.004**	**0.002**	**0.323**	**NA**
DESC	0.927	0.963	1.064	1	0.996	0.095	0	0.201	NA
Scanorama	0.959	0.982	1.767	1	0.995	0.004	0	0.417	NA
scVI	0.020	0.028	1.844	1.056	0.915	0.033	0.011	0.306	NA
scDML	0.935	0.969	1.763	1	0.997	0.033	0	0.461	NA
BBKNN	0.862	0.924	1.762	1	0.982	0.009	0.000	0.290	NA

Table S2. Evaluating the 22 integrated data of the compared methods and Beaconet using 9 metrics on two batches of DC datasets.

	NMI	ARI	iLISI	cLISI	Positive rate	Merge divergence	kBET_1(accept rate)	kBET_2(accept rate)	kBET_3(accept rate)
Seurat_0	0.756	0.754	1.91	1.013	0.894	0.006	0.859	0.939	0.901
Seurat_1	0.776	0.775	1.894	1.011	0.878	0.014	0.778	0.933	0.902
Harmony_0	0.673	0.686	1.908	1.001	0.855	0.005	0.676	0.979	0.986
Harmony_1	0.84	0.881	1.799	1.002	0.945	0.005	0.675	0.875	0.912
iMap_0	0.767	0.818	1.881	1.003	0.924	0.005	0.673	0.96	1
iMap_1	0.759	0.81	1.647	1.012	0.931	0.015	0.399	0.529	0.611
RPCI_0	0.703	0.716	1.78	1.038	0.92	0.005	0.464	0.944	0.936
RPCI_1	0.524	0.532	1.82	1.26	0.839	0.015	0.603	0.804	0.717
LIGER_1	0.771	0.779	1.933	1.021	0.888	0.005	0.646	0.984	1.0

LIGER_2	0.725	0.774	1.921	1.012	0.892	0.005	0.639	1.0	1.0
FIRM_1_2	0.841	0.864	1.298	1.0	0.943	0.013	0.176	0.287	0.330
FIRM_2_1	0.841	0.864	1.367	1.0	0.940	0.024	0.197	0.298	0.284
FastMNN_1_2	0.772	0.808	1.88	1.001	0.922	0.005	0.71	0.956	0.987
FastMNN_2_1	0.779	0.823	1.913	1.001	0.899	0.005	0.699	0.961	0.976
MNNCorrect_1_2	0.741	0.75	1.896	1	0.874	0.005	0.714	0.938	0.944
MNNCorrect_2_1	0.769	0.81	1.87	1	0.896	0.013	0.674	0.917	0.945
**Beaconet**	**0.823**	**0.857**	**1.805**	**1.001**	**0.95**	**0.005**	**0.653**	**0.945**	**0.982**
DESC	0.851	0.866	1.349	1	0.945	0.015	0.215	0.288	0.265
Scanorama	0.72	0.646	1.945	1.002	0.814	0.005	0.912	0.97	0.984
scVI	0.764	0.806	1.896	1.001	0.935	0.005	0.660	1.0	1.0
scDML	0.692	0.579	1.690	1.0	0.894	0.024	0.418	0.582	0.559
BBKNN	0.802	0.836	1.837	1.008	0.931	0.006	0.671	0.936	0.978

Table S3. The top-10 differentially expressed genes of alpha cells.

	Integrated data		Unintegrated data	
rank	names	scores	names	scores
0	GCG	97.3726	GCG	98.844154
1	TTR	94.8361	TTR	98.721275
2	CRYBA2	85.19046	GC	86.19552
3	GC	85.17424	CLU	85.616684

4	TMED6	77.0478	TMEM176B	84.62265
5	LOXL4	75.37566	IRX2	82.8414
6	KCTD12	74.57525	PCSK2	79.51732
7	PCSK2	72.60361	ALDH1A1	76.841255
8	GLS	71.83778	GPX3	73.68868
9	IRX2	69.29552	RGS4	72.770905

Table S4. The top-10 differentially expressed genes of ductal cells.

	Integrated data		Unintegrated data	
rank	names	scores	names	scores
0	S100A11	50.569275	TACSTD2	61.861443
1	SERPING1	46.14685	KRT19	61.01072
2	SPNS2	45.044052	KRT7	60.44415
3	CFTR	42.429424	PMEPA1	55.67086
4	SERINC2	41.330227	ANXA2	55.30715
5	MYL12A	38.542847	S100A11	54.285717
6	TMSB4X	38.52914	KRT18	54.100716
7	SLC4A4	37.884666	SDC4	53.969513
8	KLF6	37.62513	SERPING1	53.25547
9	PRSS8	37.617897	SERINC2	52.974068

Table S5. The top-10 differentially expressed genes of gamma cells.

	Integrated data		Unintegrated data	

rank	names	scores	names	scores
0	PPY	43.153763	PPY	43.184826
1	AQP3	28.132662	MEIS2	29.437342
2	MEIS2	27.447643	STMN2	29.281874
3	STMN2	26.406116	ETV1	28.203568
4	ETV1	26.294844	SCG2	28.133951
5	SCG2	24.927275	ID2	27.117723
6	EXPH5	22.828623	SERTM1	24.44084
7	FXYD2	22.605858	AQP3	24.430468
8	PDZK1IP1	21.986603	ARX	24.287954
9	PEG10	20.666544	PEG10	23.612286

Table S6. The top-10 differentially expressed genes of acinar cells.

	Integrated data		Unintegrated data	
rank	names	scores	names	scores
0	CTRB2	60.141922	PRSS1	60.616127
1	REG1A	58.748074	CTRB2	60.299168
2	CPA2	58.583706	REG1A	60.07749
3	CTRB1	58.262222	CPA2	59.962444
4	PRSS1	56.965137	SPINK1	59.83019
5	PLVAP	55.61805	CTRB1	59.600235
6	SPINK1	55.591614	PLA2G1B	59.46734
7	PRSS3	55.468906	CPA1	59.225113

8	CTRC	55.260387	CTRC	59.220528
9	PNLIP	55.117912	CPB1	58.673668

Table S7. The top-10 differentially expressed genes of beta cells.

	Integrated data		Unintegrated data	
rank	names	scores	names	scores
0	INS	90.10458	INS	89.43092
1	IAPP	82.47494	IAPP	82.943306
2	HADH	73.28261	HADH	74.052986
3	SYT13	67.325325	ADCYAP1	72.74722
4	SEL1L	62.73752	PCSK1	63.57074
5	PDZK1IP1	62.355038	UCHL1	63.203407
6	NPTX2	60.45106	MAFA	54.860218
7	ADCYAP1	60.201393	GAD2	54.144802
8	PRICKLE2	58.956547	DLK1	50.57577
9	SORL1	56.62096	NPTX2	49.788155

Table S8. The top-10 differentially expressed genes of delta cells.

	Integrated data		Unintegrated data	
rank	names	scores	names	scores
0	SST	51.146774	SST	51.186413
1	RBP4	45.07029	RBP4	47.01192
2	LEPR	39.464108	PCSK1	35.939495

3	PCSK1	33.82911	LEPR	35.857014
4	PDZK1IP1	29.712446	PCP4	29.477701
5	PCP4	28.439919	DHRS2	28.759796
6	GABRB3	28.147121	RGS2	27.930674
7	RGS2	28.036331	AQP3	27.201979
8	AQP3	27.276472	GABRB3	26.680187
9	ISL1	26.453701	HHEX	26.380062

Table S9. The top-10 differentially expressed genes of endothelial cells.

	Integrated data		Unintegrated data	
rank	names	scores	names	scores
0	PLVAP	27.683455	PLVAP	28.19437
1	PRICKLE2	26.935703	ENG	27.515968
2	PECAM1	26.620047	PECAM1	27.013157
3	ENG	26.435854	CD93	26.091211
4	PXDN	24.955437	PODXL	25.866394
5	SPARC	24.826212	PXDN	25.77541
6	CD93	24.589222	SPARC	25.253897
7	TCF4	24.256676	ACVRL1	25.217802
8	FLT1	24.08584	FLT1	25.11589
9	ESAM	24.01796	ESAM	24.943033

Table S10. The top-10 differentially expressed genes of macrophage cells.

	Integrated data		Unintegrated data	
rank	names	scores	names	scores
0	TYROBP	12.794236	TYROBP	12.795535
1	CD68	12.718531	CD68	12.720891
2	LAPTM5	12.087502	CD74	12.266768
3	ACP5	11.871507	LAPTM5	12.060787
4	PRICKLE2	11.599074	CTSD	11.822072
5	GOLM1	11.581709	ACP5	11.812565
6	SRGN	11.319638	CTSB	11.777216
7	CD74	11.222161	SRGN	11.736543
8	CTSB	11.183691	TMSB4X	11.567195
9	SLC38A2	11.178129	FCER1G	11.332694

Table S11. The top-10 differentially expressed genes of mast cells.

	Integrated data		Unintegrated data	
rank	names	scores	names	scores
0	TPSB2	9.783228	TPSB2	9.78381
1	LAPTM5	9.734658	LAPTM5	9.735821
2	SRGN	9.700203	SRGN	9.702009
3	SERPINB9	9.337549	GATA2	9.44679
4	CPA3	9.167597	CPA3	9.144848
5	GATA2	9.162241	RGS1	9.108482
6	RGS1	8.830535	CAPG	8.671106

7	CAPG	8.774182	AHNAK	8.511034
8	SLC38A2	8.569276	VIM	8.493204
9	AHNAK	8.566578	KIT	8.492581

Table S12. The top-10 differentially expressed genes of mesenchymal cells.

	Integrated data		Unintegrated data	
rank	names	scores	names	scores
0	SPARC	17.296886	COL12A1	17.524008
1	FN1	16.429869	SPARC	17.418924
2	COL4A1	16.4278	COL4A2	17.391602
3	LHFP	16.417192	GPX8	17.357746
4	COL1A1	16.316002	NID1	17.357336
5	COL1A2	16.300251	COL5A2	17.343407
6	COL4A2	16.145712	COL5A1	17.32513
7	COL3A1	16.09111	CDH11	17.300005
8	COL6A3	16.026062	COL3A1	17.293427
9	LGALS1	15.957589	LTBP2	17.252459

Table S13. The top-10 differentially expressed genes of MHC class II cells.

	Integrated data		Unintegrated data	
rank	names	scores	names	scores
0	CAPG	3.8583467	SLAMF7	3.8629632
1	IFI30	3.856353	PLEK	3.8620188

2	HCK	3.8547792	CAPG	3.8597107
3	TYROBP	3.8312764	PIK3R5	3.8588712
4	TNFAIP2	3.816902	GPR183	3.8581367
5	KLF6	3.7649648	IFI30	3.8574023
6	UCP2	3.7464983	BTK	3.8544645
7	CD74	3.7352715	HCK	3.8542547
8	SLC38A2	3.6861675	ITGB2	3.8538349
9	LGALS1	3.6491294	CD74	3.8490083

Table S14. The top-10 differentially expressed genes of epsilon cells.

	Integrated data		Unintegrated data	
rank	names	scores	names	scores
0	GHRL	9.152155	GHRL	9.152376
1	ACSL1	8.635522	ACSL1	8.939853
2	SPTSSB	7.443669	SPTSSB	7.5910754
3	SPINK1	6.9577646	FRZB	7.323086
4	S100A6	6.747327	CLU	7.2659783
5	FGF14	6.69581	FGF14	7.1692452
6	FRZB	6.4534016	SERPINA1	6.1392198
7	HEPACAM2	6.0010653	HEPACAM2	5.9488163
8	CLU	5.972134	S100A6	5.903534
9	CALY	5.242379	MDFIC	5.5278735

Table S15. The top-10 differentially expressed genes of schwann cells.

	Integrated data		Unintegrated data	
rank	names	scores	names	scores
0	CRYAB	6.207996	CRYAB	6.2046113
1	S100A6	5.916205	PLAUR	5.986468
2	PLAUR	5.847927	S100A6	5.876045
3	PDLIM4	5.615432	PMP22	5.6749873
4	SPARC	5.2771025	PDLIM4	5.526521
5	ANXA2	5.272416	PMEPA1	5.333046
6	CD9	5.260928	SPARC	5.2884603
7	TIMP3	5.1797953	CD9	5.1693163
8	DKK3	5.1695766	TIMP3	5.114577
9	PMP22	4.7621236	DKK3	5.1061807

Table S16. The top-10 differentially expressed genes of stellate cells.

	Integrated data		Unintegrated data	
rank	names	scores	names	scores
0	COL6A2	35.526745	COL6A2	36.45094
1	BGN	34.567375	C11orf96	35.26058
2	COL6A1	34.19128	PDGFRB	35.149857
3	PDGFRB	33.825497	BGN	35.09246
4	IGFBP4	33.776936	IGFBP4	35.041
5	C11orf96	32.99586	COL6A1	34.639816
6	SPARC	32.64817	SPARC	33.709602

7	COL3A1	32.212856	SERPINH1	33.06728
8	SERPINH1	31.832418	IGFBP7	32.297226
9	COL1A2	31.39049	COL3A1	32.04699

Table S17. The top-10 differentially expressed genes of T cells.

	Integrated data		Unintegrated data	
rank	names	scores	names	scores
0	CD52	4.5778756	CD52	4.5778756
1	LAPTM5	4.532293	LAPTM5	4.5323815
2	IL32	4.3640623	TMSB4X	4.2923183
3	SERPINB9	4.2324576	IFITM2	3.994035
4	TMSB4X	3.9706671	CCL5	3.8599472
5	IFITM2	3.958695	LCP1	3.8580847
6	CCL5	3.921936	RAC2	3.8522317
7	RAC2	3.9066384	IL32	3.7981799
8	LCP1	3.8885472	ZFP36L2	3.5090308
9	ALDOB	3.8038554	SPOCK2	3.2076435

Table S18. The positive rates of the full Beaconet and the variant Beaconet without BS-Norm on two-batch DC datasets, five-batch human pancreatic datasets, three-batch cell line datasets, two-batch Tabula muris datasets, limb_muscle, lung, mammary_gland, marrow, spleen, tongue, trachea.

	Beaconet (full)	Variant Beaconet (without BS-Norm)

DC	0.950	0.935
Cell Line	0.994	0.993
Pancreatic	0.949	0.967
Tabula muris (tissue-level)	0.650	0.732
Limb_Muscle	0.851	0.897
Lung	0.915	0.940
Mammary_Gland	0.969	0.977
Marrow	0.686	0.778
Spleen	0.950	0.960
Tongue	0.935	0.952
Trachea	0.974	0.978

Table S19. The one-sided permutation test P values for determine whether BS-Norm improves the performance of Beaconet.

	Beaconet2Variant	Beaconet2Unintegrated	Variant2Unintegrated
Pancreatic	0	0	0
DC	0.471347135	0	0
Cell line	0	0	0
Tabula muris	0	0	0
Limb_Muscle	0	0	0
Lung	0	0	0
Mammary_Gland	0	0	0.00480048
Marrow	0.00050005	0.00010001	0.273727373
Spleen	0	0	0

Tongue	0	0	0.00020002
Trachea	0	0	0

Table S20. The rank-sum test P values for determine whether BS-Norm improves the performance of Beaconet.

	Beaconet2Variant	Beaconet2Unintegrated	Variant2Unintegrated
Pancreatic	0	0	0
DC	0.734721	1.27E-16	3.10E-17
Cell line	1.45E-37	0	0
Tabula muris	0	0	0
Limb_Muscle	0	0	1.37E-15
Lung	2.62E-104	2.56E-263	8.69E-52
Mammary_Gland	3.37E-58	5.14E-69	0.033397
Marrow	0.089444	0.338164	0.730912
Spleen	0	0	0.068166
Tongue	1.94E-27	1.13E-33	0.012282
Trachea	0	0	3.82E-15

What is LISI metric?

LISI (**L**ocal **I**nverse **S**impson **I**ndex) is a batch effect correction metric based on Simpson Index. The original aim of SI (**S**impson **I**ndex) is to indicate species diversity. The definition of Simpson Index is the probability that two individuals belong to the same species, if they are randomly selected from an infinite community.

Simpson Index: Assumes there are m species in an environment. The i-th species has n_i samples. The fraction of species i is $f_i = \frac{n_i}{\sum_{j=1}^{m} n_j}$.

If assuming that the environment is big enough, the sampling can be approximate as a sampling with replace. Simpson Index $D = \sum_{i=1}^{m} f_i^2$. The smaller D means the species in the environment is more diverse. The maximum value of D is 1, if and only if there are one species in the environment. The minimum value of D is $\frac{1}{m}$, if and only if the number of each species is equal, which means $f_i = \frac{1}{m}$ for *i=1,2,3,...,m*.

LISI: The definition of inverse of Simpson index in LISI is $\frac{1}{\sum_{i=1}^{m} f_i^2}$.

The 'local' means the inverse of Simpson index is calculated based on the *k*-neighbors of each cell, and the f_i means the fraction of the cells with label *i* in the *k*-neighbors set. When the label is to indicate batch label, the LISI is called iLISI. When the label is to indicate the cell type, the LISI is called cLISI.

The optimal iLISI

The higher iLISI indicates the batch labels of the local neighbors of given cell are more diversity, while we could observed that the bigger iLISI is not means the better batch mixed (**Fig S2**). The question is: what is the maximum value of iLISI *without overcorrection.*

Opinion 1 [11]: if cell type *A* is not detected in batch *B*, the *A* cells could not be merged with the cells in batch *B*.

More generally speaking, the cell types are not identical in each batch. If cell type A not exist in p batches, and do not detected in $m-p$ batches. Hence, for each cell with cell type A, the

maximum value of iLISI for cell *A* is achieved when the cell *A* is surrounding by the cells from the $m-p$ batches uniformly. Therefore, the iLISI could not bigger than p without overcorrection. Liu Y et al. [11] adjusted the iLISI bigger than *p* when evaluating RPCI.

Opinion 2 [9]: even when cell type *A* is detected in *p* batches of total *m* batches, the iLISI could not achieve *p* without overcorrection, because the proportion of cell type *A* in the *p* batches is usually not uniform.

If the batch effect is removed clearly, the batch label should be an irrelative variable for the cell type label. We assume the proportion of cell *A* in the *m* batch is $\hat{f}_1, \hat{f}_2, ..., \hat{f}_m$. ($f_j = 0$ if cell type *A* is not detected in batch *j*). Therefore, it is a tighter upper bound of iLISI without overcorrection for cell type *A*

$$iLISI_{best} = \frac{1}{\sum_{j=1}^{m} \hat{f}_j^2} .$$

Specially, when there are *p* batches uniformly detect cell type *A*, and *m-p* batches do not detect cell type *A*, the maximum iLISI in **Opinion 2** is *p*, which is agree with **Opinion 1**.

Estimate the optimal iLISI for cell line dataset

The cell line dataset contains 2996 293T cells in batch 1, 3258 Jurkat cells in batch 2, and 1605 293T cells and 1783 Jurkat cells in batch 3. The proportion of 293T cells in three batches is 2996:0:1605, and the proportion of the Jurkat cells in three batches is 0:3258:1783. Therefore, for each 293T cell, the optimal iLISI is $\frac{1}{(\frac{2996}{2996+1605})^2 + 0 + (\frac{1605}{2996+1605})^2} = 1.8326$, and for each Jurkat cell, the optimal iLISI is $\frac{1}{0 + (\frac{3258}{3258+1783})^2 + (\frac{1783}{3258+1783})^2} = 1.8423$. The summary of the optimal iLISI of all cells is 1.838 by mean and 1.842 by median.

The drawback of LISI: iLISI may assigns the same score for the datasets with different batch-effect removal quality of batch effect. Especially, it may give an optimal score to overcorrection result. The reason is LISI only considers the local proportion of given cell type in numerical, but ignore the matching of local fraction and global fraction by cell type.

Here, we give a top example to illustrate this problem.

Table S21. The cell numbers with each cell type and batch.

	Batch 1	Batch 2	Batch 3
Cell type 1	100	100	0
Cell type 2	130	0	170
Cell type 3	160	0	90

In this case, for each cell with cell type 1, the optimum iLISI is

$$\frac{1}{(\frac{100}{100+100})^2+(\frac{100}{100+100})^2+(\frac{0}{100+100})^2}=2$$
.

We will display two cell allocations of a well-corrected cell and an overcorrected cell below, while both of their iLISI are 2.

We assume the $k=3$ in k-neighbors. For a well-corrected cell with cell type 1 in batch 1, its local cell allocation is in Table S22. The neighbors of this cell is the cell type 1 from batch 1 and batch 2. The iLISI is $\frac{1}{(\frac{3}{3+3})^2+(\frac{3}{3+3})^2+(\frac{0}{3+3})^2}=2$

Table S22. The local neighbors' allocation of a well corrected cell with cell type 1.

	Batch 1	Batch 2	Batch 3

Cell type 1	3	3	0
Cell type 2	0	0	0
Cell type 3	0	0	0

For an overcorrected cell with cell type 1 in batch 1, its local cell allocation is in Table S23. The cell does not merged with the other cell with types 1 in batch 2, but mismatched with cell type 2 and cell type 3. The iLISI of this cell is $\frac{1}{(\frac{2+1}{2+1+3})^2+0+(\frac{3}{2+1+3})^2}=2$

Table S23. The local neighbors' allocation of an overcorrected cell with cell type 1.

	Batch 1	Batch 2	Batch 3
Cell type 1	2	0	0
Cell type 2	1	0	3
Cell type 3	0	0	0

Therefore, a batch effect correction method get a better iLISI by two ways. The first is that the method get a well corrected result. The second is the result is overcorrected, and this problem is difficult to solve by adjusting iLISI value. We suggest that researchers consider multiple metrics comprehensively when evaluating the batch effect correction methods.

Note: The difference between iLISI and cLISI is that the first one calculates score using batch label and the second one using cell type label.

Table S24. The detail information of experimental platform.

Platform	Version
Windows	Windows 10 64bit 16GB Inter(R) Core(TM) i7-9700F
GPU	NVIDIA GeForce GTX 1660 Ti (14GB memory)
CUDA	10.1.105
R	4.0.4
Python	3.6.8
RStudio	1.2.1335

We noted that FIRM for cell line data integration task was run on a Windows Server since it requires extensive computational resource compared with other methods. The parameter of this Windows Server is described below: ADM Ryzen Treadripper PRO 5955WX 16-Cores 4.00GHZ. This sever has 16 physical cores, 32 logical cores, 256GB RAM memory.

Table S25. The R packages and the version number used in our study.

R package	Version
Seurat	4.0.0
SeuratObject	4.0.2
Umap	0.2.7.0
Batchelor	1.6.3
Scran	1.18.5
Harmony	0.1.0
RISC	1.0

Note: We use the command ‘options(future.globals.maxSize = 6400 * 1024^2)’ to relax the limitation of the size of global object in R environment.

We set the seed of random number as 42 for all R script using command ‘set.seed(42)’.

Table S26. The Python packages and the version number used in our study.

**Python package**	**Version**
Scipy	1.5.4
Seaborn	0.9.0
Pandas	1.1.5
Torch	1.8.1+cu101
Tqdm	4.62.2
Numpy	1.19.5
Matplotlib	3.3.4
Scanpy	1.7.1
Tensorflow	1.7.0
umap-learn	0.5.1
scikit-learn	0.24.1
Imap	1.0.0
Desc	2.1.1
Scanorama	1.7.1

We noted that the environment for scDML integration method is different, as the author specifies the required environment for scDML at their GitHub (https://github.com/eleozzr/scDML).

Table S27. The hyper-parameters of all methods and functions used in our study.

**Method**	**Function**	**Parameters**
Scanorama	scanorama.correct	return_dimred=False,<br>batch_size=BATCH_SIZE, verbose=VERBOSE,

		ds_names=None,<br>dimred=DIMRED,<br>approx=APPROX,<br>sigma=SIGMA,<br>alpha=ALPHA,<br>knn=KNN,<br>return_dense=False,<br>hvg=None,<br>union=False,<br>seed=0
iMAP	stage1.iMAP_fast	n_epochs = 150,<br>num_workers=0,<br>lr = 0.0005,<br>b1 = 0.5,<br>b2 = 0.999,<br>latent_dim = 256,<br>n_critic = 5,<br>lambda_co = 3,<br>lambda_rc = 1,<br>seed = 8,
	stage2.integrate_data	n_top_genes=None,<br>pp=False,<br>n_epochs=150,<br>inc=False,<br>metric='angular',<br>k1=None,

		k2=None,<br>n_batch=2,<br>n_sample=3000,<br>seed=8
RPCI	scMultiIntegrate	eigens = 10,<br>add.Id = NULL,<br>var.gene = NULL,<br>method = "RPCI",<br>align = "OLS",<br>npc = 50,<br>adjust = TRUE,<br>ncore = 1,<br>do.fast = "AUTO",<br>seed = 123
FastMnn	multiBatchPCA	d = 50,<br>subset.row = NULL,<br>weights = NULL,<br>get.all.genes = FALSE,<br>get.variance = FALSE,<br>preserve.single = FALSE,<br>assay.type = "logcounts",<br>BSPARAM = IrlbaParam(),<br>deferred = TRUE,<br>BiocParallel::bpparam()
	reducedMNN	k = 20,

		prop.k = NULL,<br>restrict = NULL,<br>ndist = 3,<br>auto.merge = FALSE,<br>min.batch.skip = 0,<br>BNPARAM = KmknnParam(),<br>BPPARAM = SerialParam()
MNNCorrect	mnnCorrect	restrict = NULL,<br>k = 20,<br>prop.k = NULL,<br>sigma = 0.1,<br>cos.norm.in = TRUE,<br>cos.norm.out = TRUE,<br>svd.dim = 0L,<br>var.adj = TRUE,<br>subset.row = NULL,<br>correct.all = FALSE,<br>auto.merge = FALSE,<br>assay.type = "logcounts",<br>BSPARAM = ExactParam(),<br>BNPARAM = KmknnParam(),<br>BPPARAM = BiocParallel::bpparam()

DESC	environment	os.environ['PYTHONHASHSEED'] = '0'
	sc.pp.scale	max_value=6
	desc.train	dims=[adata.shape[1],64,32],<br>tol=0.005,<br>n_neighbors=10,<br>batch_size=256,<br>louvain_resolution=[0.8,1.0],<br>save_dir="./temp",<br>do_tsne=True,<br>learning_rate=200,<br>use_GPU=gpu,<br>num_Cores=1,<br>num_Cores_tsne=1,<br>save_encoder_weights=False,<br>save_encoder_step=3,<br>use_ae_weights=False,<br>do_umap=False
Seurat	FindIntegrationAnchors	assay = NULL,<br>reference = NULL,<br>scale = TRUE,<br>normalization.method = c("LogNormalize", "SCT"),<br>sct.clip.range = NULL,<br>reduction = c("cca", "rpca"),<br>l2.norm = TRUE,

		dims = 1:30,<br>k.anchor = 5,<br>k.filter = 200,<br>k.score = 30,<br>max.features = 200,<br>nn.method = "annoy",<br>n.trees = 50,<br>eps = 0,<br>verbose = FALSE
	IntegrateData	new.assay.name = "integrated",<br>normalization.method = c("LogNormalize", "SCT"),<br>features = NULL,<br>features.to.integrate = NULL,<br>dims = 1:30,<br>k.weight = 100,<br>weight.reduction = NULL,<br>sd.weight = 1,<br>sample.tree = NULL,<br>preserve.order = FALSE,<br>eps = 0,<br>verbose = FALSE
Harmony	HarmonyMatrix	do_pca = TRUE,<br>npcs = 20,

		theta = NULL,<br>lambda = NULL,<br>sigma = 0.1,<br>nclust = NULL,<br>tau = 0,<br>block.size = 0.05,<br>max.iter.harmony = 10,<br>max.iter.cluster = 200,<br>epsilon.cluster = 1e-05,<br>epsilon.harmony = 1e-04,<br>plot_convergence = FALSE,<br>return_object = FALSE,<br>verbose = FALSE,<br>cluster_prior = NULL
scVI	scvi.model.SCVI	n_hidden=128,<br>n_Latent=10,<br>n_layers=1,<br>dropout_rate=0.1,
LIGER	optimizeALS	k=20,<br>lambda=5,<br>thresh=1e-6,<br>max.iter=30,<br>nrep=1,<br>use_unshared=FALSE,<br>rand.seed=1

	quantile_norm	quantiles=50,<br>min_cells=20,<br>knn_k=20,<br>dim.use=1:ncol(H[[1]]),<br>do.center=FALSE,<br>max_sample=1000,<br>eps=0.9,<br>refine_knn=TRUE,<br>“ ref_dataset” is traversed from 1 to n_batches.
BBKNN	bbknn.bbknn	neighbors_within_batch=3,<br>n_pcs=50,<br>trim=None,<br>approx.=True,<br>n_trees=10,<br>use_faiss=True,<br>metric=”angular”,<br>set_op_mix_ratio=1,<br>local_connectivity=1
FIRM	FIRM	dims=10,<br>all_genes=FALSE,<br>res_seq_SS2=seq(0.1,2,0.1),<br>res_seq_tenx=seq(0.1,2,0.1),<br>coreNum=30
scDML	scDML.scDMLModel.preprocess	cluster_method=”louvain”,<br>resolution=3.0,

		n_high_var=2000,<br>normalize_samples=False,<br>target_sum=1e4,<br>log_normalize=False,<br>normalize_feature=False,<br>pca_dim=100
	scDML.scDMLModel.integrate	merge_rile="rule2"
k-means	sklearn.cluster.k_means	sample_weight=None,<br>init='k-means++', precompute_distances='deprecated', n_init=20,<br>max_iter=300,<br>verbose=False,<br>tol=1e-4,<br>random_state=None,<br>copy_x=True,<br>n_jobs='deprecated',<br>algorithm="auto",<br>return_n_iter=False
PCA	sklearn.decomposition.PCA	copy=True,<br>whiten=False,<br>svd_solver='auto',<br>tol=0.0,<br>iterated_power='auto',<br>random_state=None
UMAP(python)	umap.UMAP	n_neighbors=30,

		min_dist=0.3,<br>metric='cosine',<br>n_components=2,<br>learning_rate=1.0,<br>spread=1.0,<br>set_op_mix_ratio=1.0,<br>local_connectivity=1,<br>repulsion_strength=1,<br>negative_sample_rate=5,<br>angular_rp_forest=False,<br>verbose=False

- The numbers of dimensions of the integration space are varying from 30 to 2000. For the purpose of comparison of methods fairly, we performs the k-means clustering in the 2-dimensional UMAP space with the ground truth number of clusters.

- For the batch effect correction methods, we used the default parameters of the specific package version installed on our machine, except the merge order or reference.

- The reference of RPCI is implied by the first matrix in the list of batches.

- The result of RPCI is to transform the data into a 50-dimensional principle components space in default. However, only the first k principle components are informatics dimension. We select the first 10 principle components of RPCI as its result, since the script code provided in the supplementary files of RPCI uses the first 10 principle to perform the downstream analysis, e.g. calculation of metrics, clustering and so on.

- As a practical trick of performing UMAP embedding, we first reduce the dimension of the integration matrix to 30-dimensional space using PCA, and then call the UMAP package to transform the data into 2-dimensional UMAP space. (the first 10 principle component of the

result of RPCI is directly feed to UMAP without PCA, since its principle component has been calculated in the integration method)

- The parameter of UMAP is referred to the coding of the visualization process of the code script of iMAP.

Supplementary Figures

Fig S1. UMAP projection of the results of Beaconet and eight methods with all possible reference batches and orderings of batches on the three cell line datasets. These batch-effect removal methods include Harmony, Seurat, MNNCorrect, iMAP, RPCI, FastMNN, DESC, and Scanorama.

Fig S2. UMAP projection of the results of unintegrated data and five methods with all possible reference batches and orderings of batches on the three cell line datasets. These batch-effect removal methods include BBKNN, scVI, scDML, FIRM, LIGER.

Fig S3. UMAP projection of the results of Beaconet and the eight methods with all possible reference batches and orderings of batches on the two DC datasets. These batch-effect removal methods include Harmony, Seurat, MNNCorrect, iMAP, RPCI, FastMNN, DESC, and Scanorama.

Fig S4. UMAP projection of unintegrated data and results five methods with all possible reference batches and orderings of batches on the two-batch DC datasets. These batch-effect removal methods include BBKNN, scVI, scDML, FIRM, LIGER.

Fig S5. The PMD metric for intuitive observing the single cell-level batch effect on the results of three cell line datasets. **a.** Beaconet. **b.** Scanorama. **c.** DESC. **d.** RPCI with reference batch 1. **e.** FastMNN with batch-ordering “2_1_3”. **f.** RPCI with reference batch 2. **g.** iMAP with batch-ordering “2_1_3”. **h.** RPCI with reference batch 3.

Fig S6. The PMD metric for intuitive observing the single cell-level batch effect on the results of two DC datasets. **a.** Beaconet. **b.** Scanorama. **c.** Seurat with reference batch 1. **d.** Seurat with reference batch 2. **e.** Harmony with reference batch 1. **f.** Harmony with reference batch 2. **g.** RPCI with reference batch 1. **h.** iMAP with reference batch 1.

Fig S7. The PMD metric for intuitive observing the single cell-level batch effect on the results of five human pancreatic datasets. **a.** Beaconet. **b.** Seurat with reference batch 1. **c.** Seurat with reference batch 5.

d. Scanorama. **e.** Scanorama **f.** iMAP with ordering “1_2_3_4_5”. **g.** RPCI with reference batch 4. **h.** RPCI with reference batch 3. **i.** FastMNN with ordering “1_2_3_4_5”.

We provide the visualization of all results in figshare: https://doi.org/10.6084/m9.figshare.20764843, including over 400 figure in “observing_md” folder. The figures in **Fig S4-S6** are representative data for exhibiting the effectiveness of PMD metric and the impact of selection of reference on integrated pancreatic dataset.

Fig S8. UMAP projection of five integrated human pancreatic datasets with artificial missing cell types using Beaconet. **a.** Miss alpha cells in “Baron” batch. **b.** Miss beta cells in “Baron” batch. **c.** Miss delta cells in “Baron” batch. **d.** Miss ductal cells in “Baron” batch. **e.** Miss gamma cells in “Baron” batch.

Fig S9. The UMAP projection of integrated and unintegrated Tabula muris datasets. **a.** The unintegrated two batches of Tabula muris datasets, colored by batches. **b.** The unintegrated two batches of Tabula muris datasets, colored by tissues. **c.** The integrated data of Tabula muris datasets using Beaconet, colored by batches. **d.** The integrated data of Tabula muris datasets using Beaconet, colored by tissues.

Fig S10. The visualization of unintegrated data of twelve tissues in Tabula muris. **a.** Bladder. **b.** Liver. **c.** Lung. **d.** Mammary Gland. **e.** Kidney. **f.** Heart. **g.** Heart and Aorta. **h.** Large_intestine. **i.** Limb_muscle. **j.** Brain_Myeloid. **k.** Brain Non-Myeloid. **l.** Fat.

Fig S11. The visualization of unintegrated data of seven tissues in Tabula muris. **a.** Marrow. **b.** Pancreas. **c.** Skin. **d.** Spleen. **e.** Thymus. **f.** Tongue. **g.** Trachea.

Fig S12. UMAP projection of twelve two-batch tissues in Tabula muris integrated using Beaconet. **a.** Bladder. **b.** Liver. **c.** Lung. **d.** Mammary Gland. **e.** Kidney. **f.** Heart. **g.** Heart and Aorta. **h.** Large_intestine. **i.** Limb_muscle. **j.** Brain_Myeloid. **k.** Brain Non-Myeloid. **l.** Fat.

Fig S13. UMAP projection of seven two-batch tissues in Tabula muris integrated using Beaconet. **a.** Marrow. **b.** Pancreas. **c.** Skin. **d.** Spleen. **e.** Thymus. **f.** Tongue. **g.** Trachea.

Fig S14. The effectiveness of the feature space of Beaconet. **a.** The expression pattern of the marker genes for major cell types in original data. **b.** The expression pattern of the marker genes for major cell types in the integrated data of Beaconet. **c.** The expression pattern of the differentially expressed genes for all cell types in original data. **d.** The expression pattern of the differentially expressed genes for all cell types in integrated data of Beaconet.

Note: 300 differentially expressed genes are included in the Fig S13c, d. The 300 genes contain the top-10 differentially expressed genes for 15 cell types in original dataset and integrated dataset. (300=15*10+15*10).

Fig S15. The fluctuation of the memory usage of the batch-effect removal methods. **a.** The expression pattern of the marker genes for major cell types in original data. **b.** The expression pattern of the marker genes for major cell types in the integrated data of Beaconet. **c.** The expression pattern of the differentially expressed genes for all cell types in original data. **d.** The expression pattern of the differentially expressed genes for all cell types in integrated data of Beaconet.

Note: The memory consumption of these methods were records using python package "memory_profiler". The figures were generated by command "mprof plot".

Fig S16. UMAP projection of the integrated data of the variant Beaconet on several datasets, including the three-batch cell line datasets, two-batch DC datasets, five-batch human pancreatic datasets, the seven tissues of mouse, including Lung, Mammary_gland, Marrow, Spleen, Limb_Muscle, Tongue and Trachea, on cell-type level and Tabula muris on tissue-level.

Note: the visualization of the full Beaconet on these datasets have provided in **Fig. 4a** and **Fig. S1, S3, S12, S13**.

Fig S17. Comparison of the distribution of merge divergence for the full Beaconet and the variant method that disable the BS-Norm module.

Note: We compared the integrated data of Beaconet and the variant method with the unintegrated data on the three-batch cell line datasets, two-batch DC datasets, five-batch human pancreatic datasets, the seven tissues of mouse, including Lung, Mammary_gland, Marrow, Spleen, Limb_Muscle, Tongue and Trachea, on cell-type level and Tabula muris on tissue-level.

Fig S18. Visualization of the integrated data during training process using human pancreatic datasets. **a.** Integration of "Mutaro" batch and "Segerstolpe" batch. **b.** Integration of "Mutaro" batch and "Wang" batch. **c.** Integration of "Segerstolpe" batch and "Wang" batch.

Fig S19. Beaconet is robust to the three scaling methods. The performance of Beaconet on three integration tasks with "scaling on the whole dataset", "scaling on each batch" and "no scaling" input data. **a.** UMAP visualization of integrated DC datasets in three scaling methods. The scatterplots were colored by cell type, batch label, PMD scores respectively. **b.** UMAP visualization of integrated Cell Line datasets in three scaling methods. **c.** UMAP visualization of integrated Human pancreas datasets in three scaling methods. **d.** Comparison of performance of Beaconet on three scaling methods using three integration tasks.

Fig. S20. Seurat integrates two-batch DC data with scaling on the whole dataset, scaling on each dataset, and no scaling.

Fig. S21. Seurat integrates three-batch cell line data with scaling on the whole dataset, scaling on each dataset, and no scaling.

Fig. S22. Seurat integrates five-batch human pancreas data with scaling on the whole dataset, scaling on each dataset, and no scaling.

Fig. S23. FastMNN integrates two-batch DC data with scaling on the whole dataset, scaling on each dataset, and no scaling.

Fig. S24. FastMNN integrates three-batch cell line data with scaling on the whole dataset, scaling on each dataset, and no scaling.

Fig. S25. FastMNN integrates five-batch human pancreas data with scaling on the whole dataset, scaling on each dataset, and no scaling.

Fig. S26. scDML integrates two-batch DC data, three-batch cell line data, and five-batch human pancreas data with scaling on the whole dataset, scaling on each dataset, and no scaling.

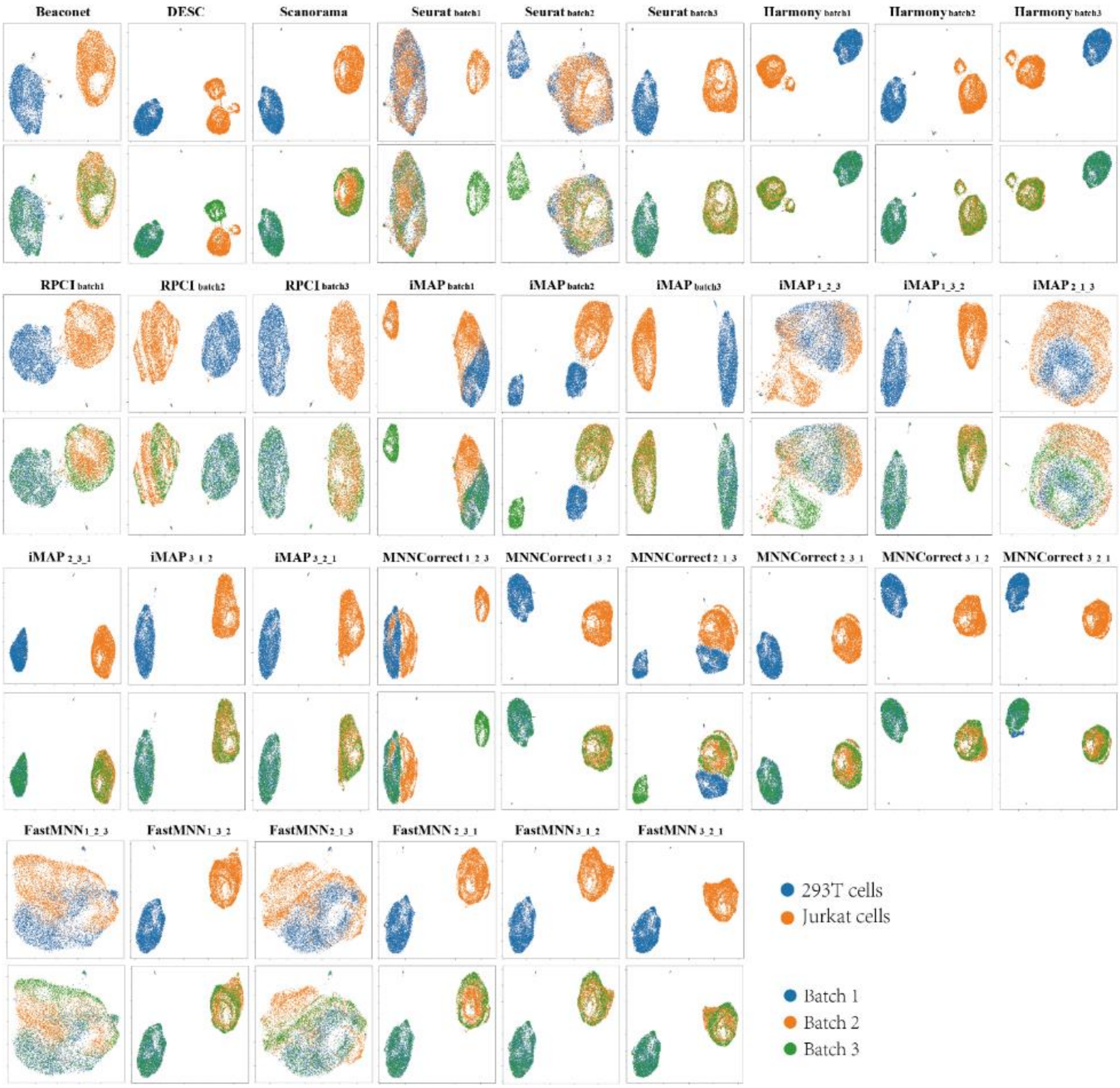

Fig S1. UMAP projection of the results of Beaconet and eight methods with all possible reference batches and orderings of batches on the three cell line datasets. These batch-effect removal methods include Harmony, Seurat, MNNCorrect, iMAP, RPCI, FastMNN, DESC, and Scanorama.

Note: The batch variance could be observed clearly by the UMAP visualization with assigning color by the cell types (the odd rows) and batches (the even rows).

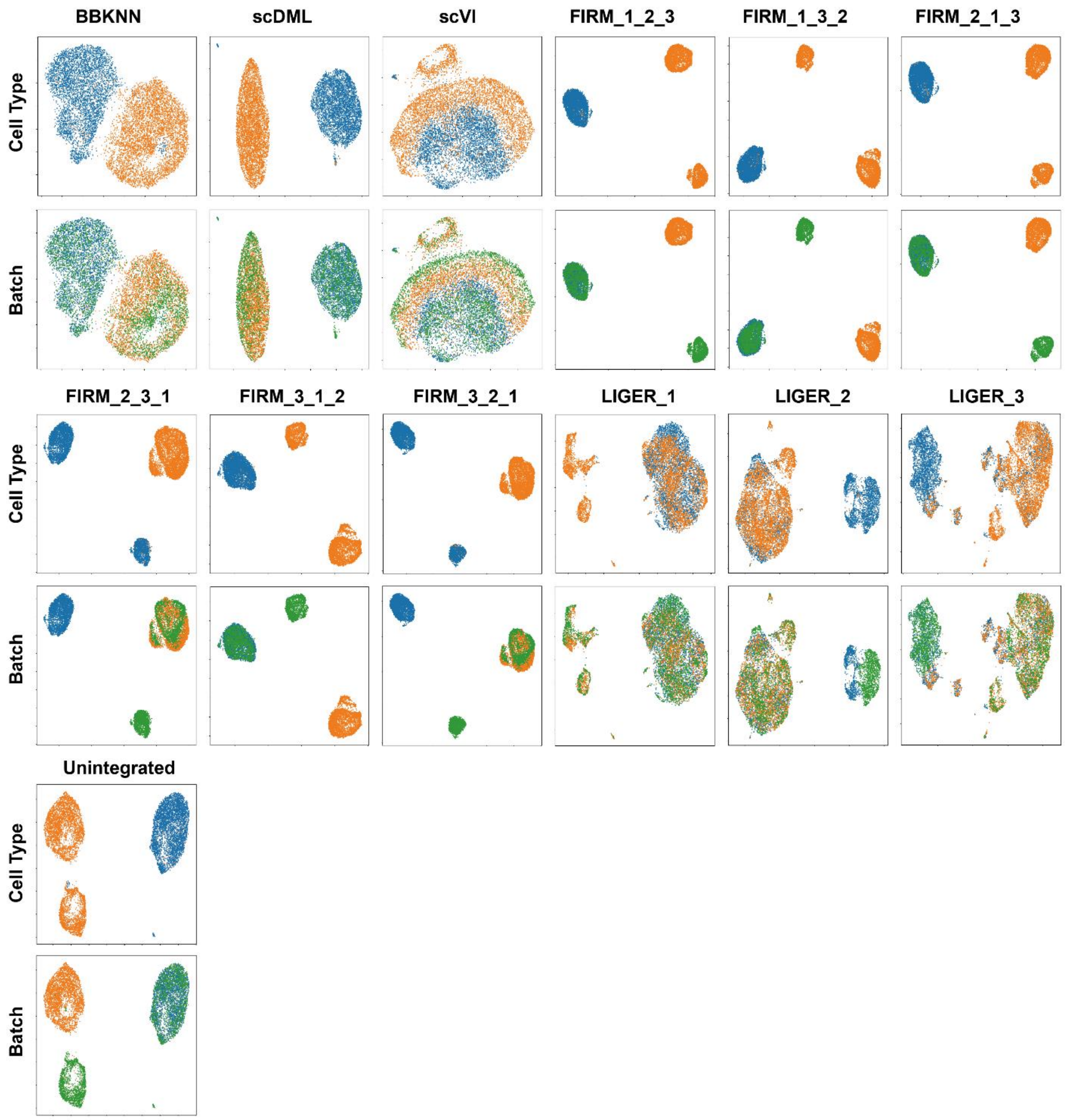

Fig S2. UMAP projection of the results of unintegrated data and five methods with all possible reference batches and orderings of batches on the three cell line datasets. These batch-effect removal methods include BBKNN, scVI, scDML, FIRM, LIGER.

Note: The batch variance could be observed clearly by the UMAP visualization with assigning color by the cell types (the odd rows) and batches (the even rows).

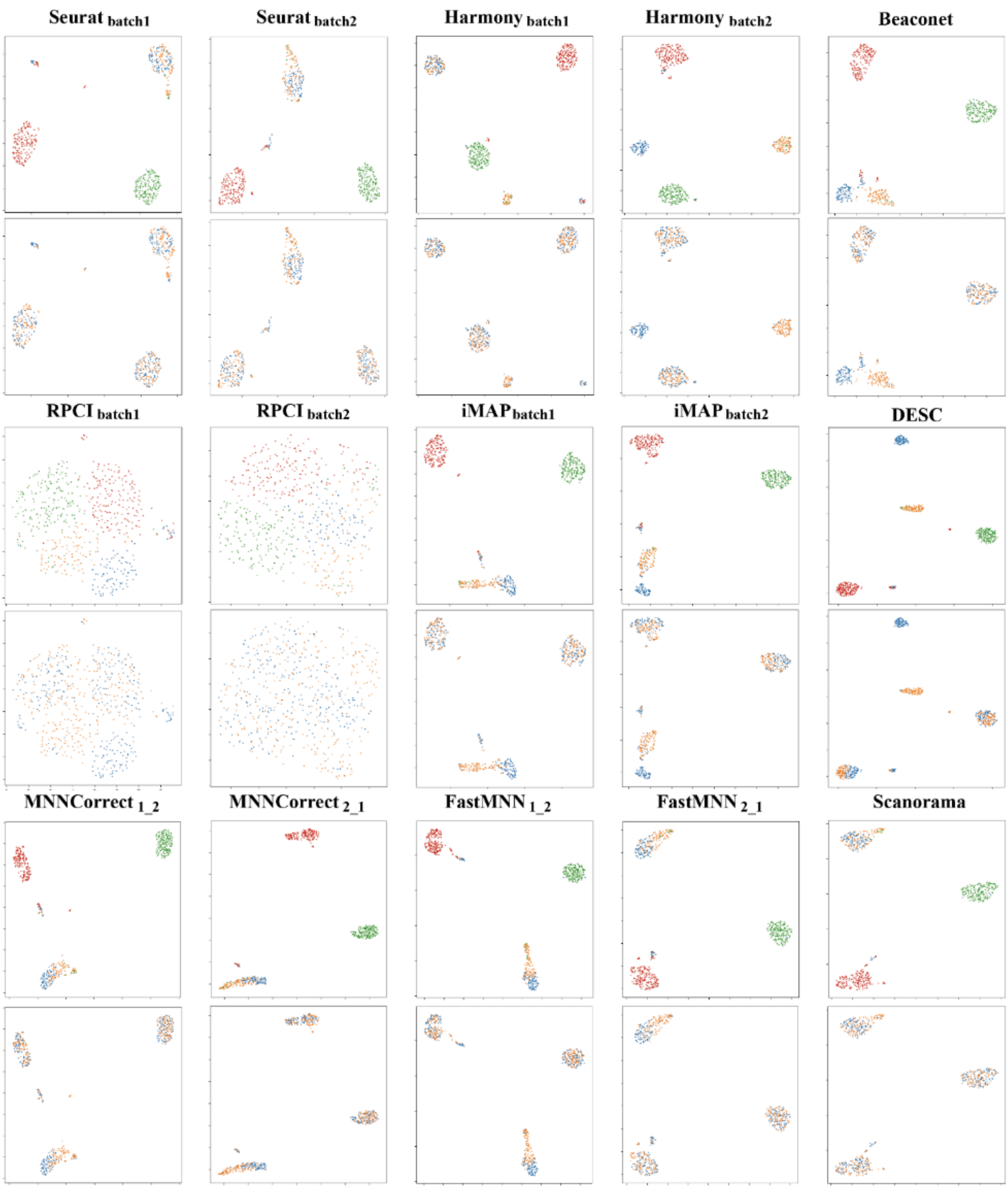

Fig S3. UMAP projection of the results of Beaconet and the eight methods with all possible reference batches and orderings of batches on the two-batch DC datasets. These batch-effect removal methods include Harmony, Seurat, MNNCorrect, iMAP, RPCI, FastMNN, DESC, and Scanorama.

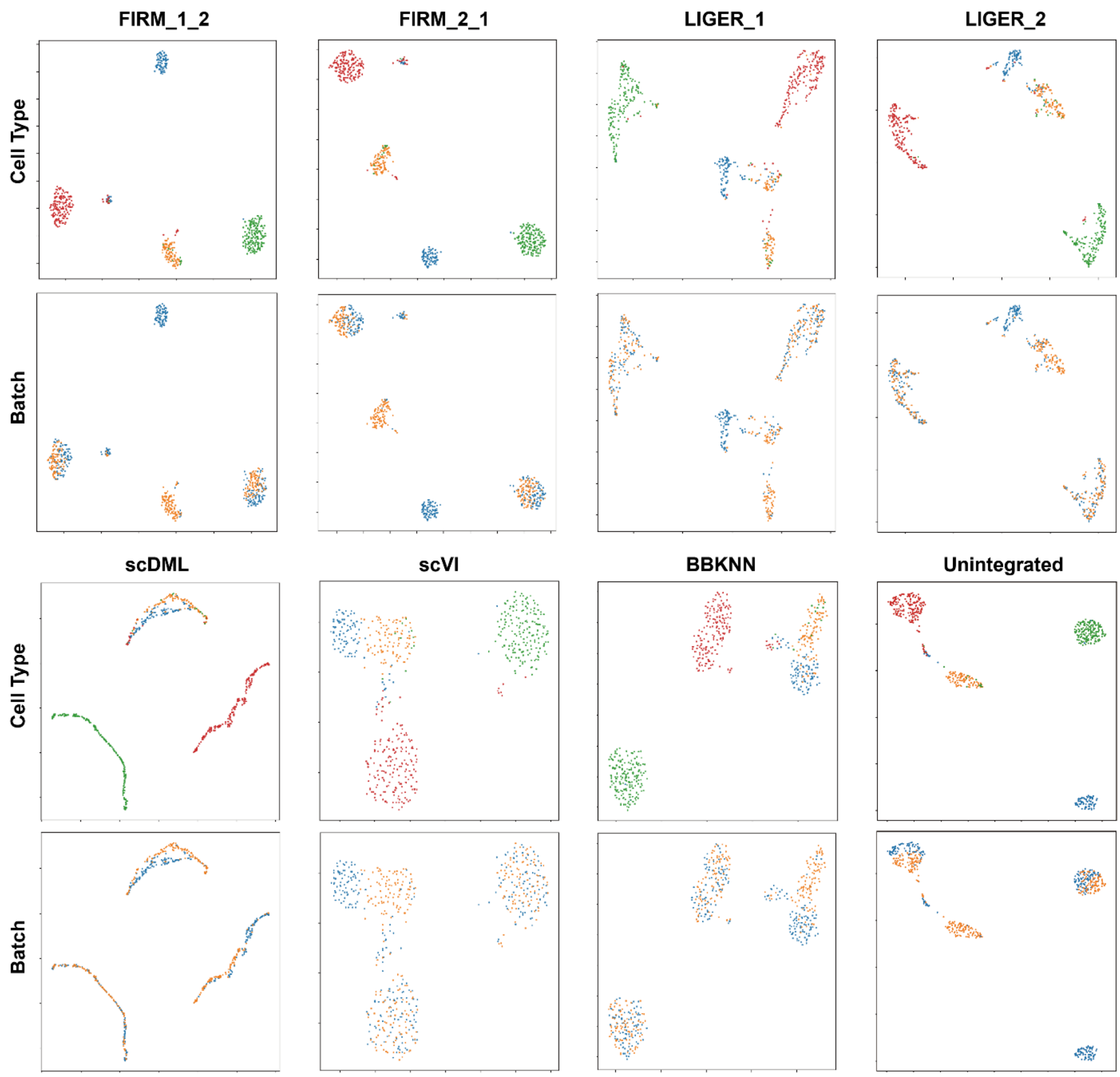

Fig S4. UMAP projection of unintegrated data and results five methods with all possible reference batches and orderings of batches on the two-batch DC datasets. These batch-effect removal methods include BBKNN, scVI, scDML, FIRM, LIGER.

Note: The batch variance could be observed clearly by the UMAP visualization with assigning color by the cell types (the odd rows) and batches (the even rows).

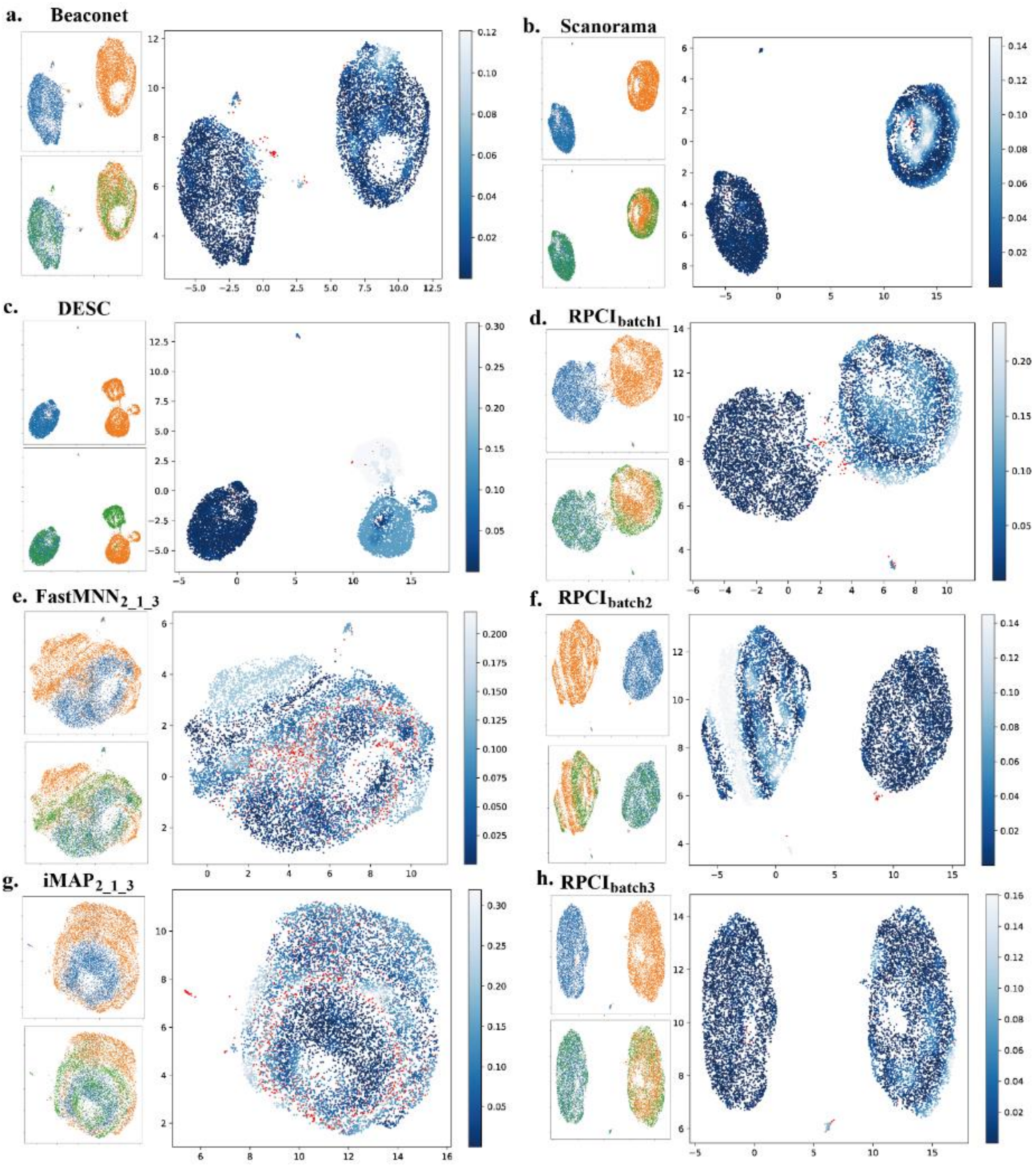

Fig S5. The PMD metric for intuitive observing the single cell-level batch effect on the results of three cell line datasets. **a.** Beaconet. **b.** Scanorama. **c.** DESC. **d.** RPCI with reference batch 1. **e.** FastMNN with batch-ordering “2_1_3”. **f.** RPCI with reference batch 2. **g.** iMAP with batch-ordering “2_1_3”. **h.** RPCI with reference batch 3.

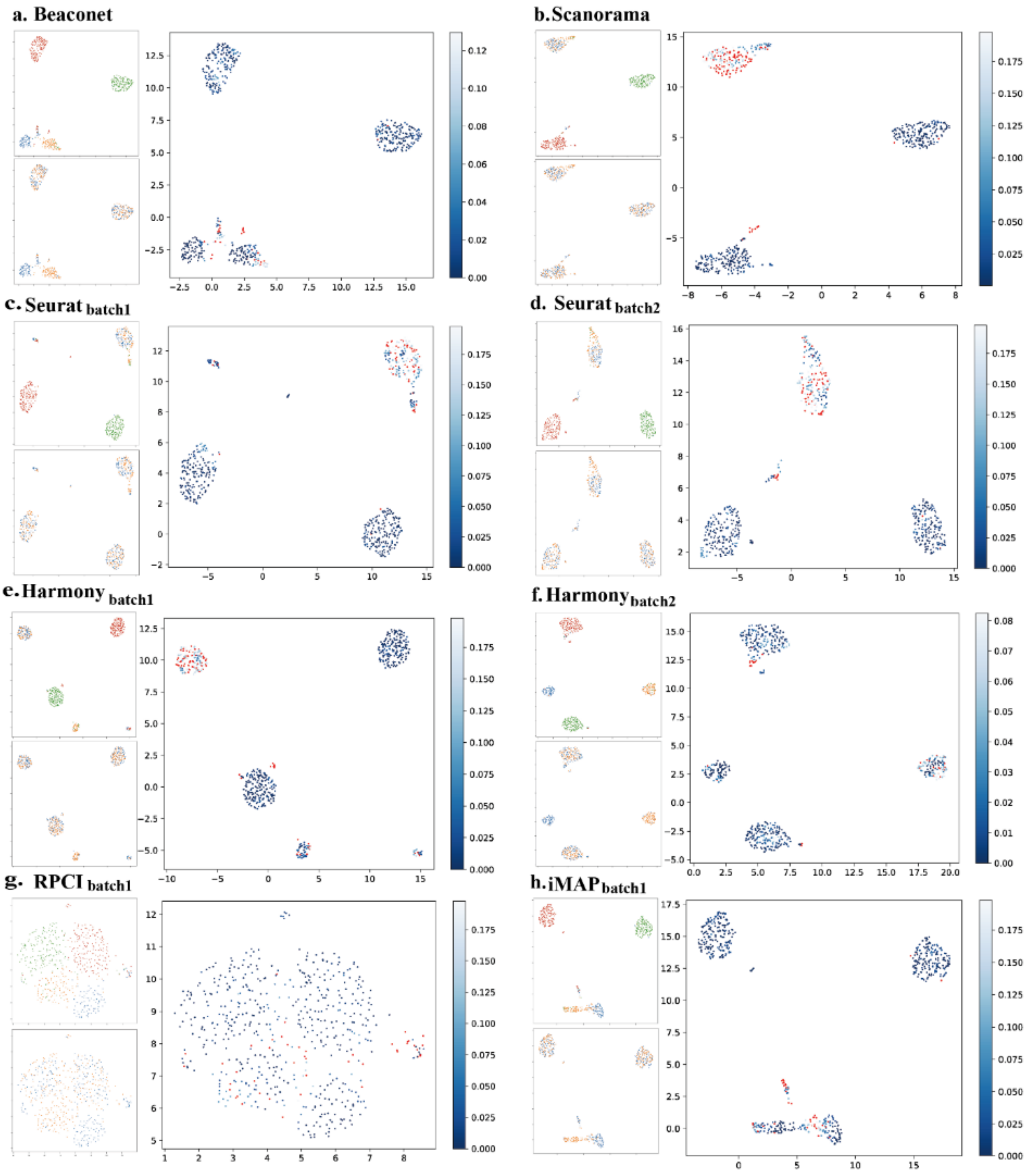

Fig S6. The PMD metric for intuitive observing the single cell-level batch effect on the results of two DC datasets. **a.** Beaconet. **b.** Scanorama. **c.** Seurat with reference batch 1. **d.** Seurat with reference batch 2. **e.** Harmony with reference batch 1. **f.** Harmony with reference batch 2. **g.** RPCI with reference batch 1. **h.** iMAP with reference batch 1.

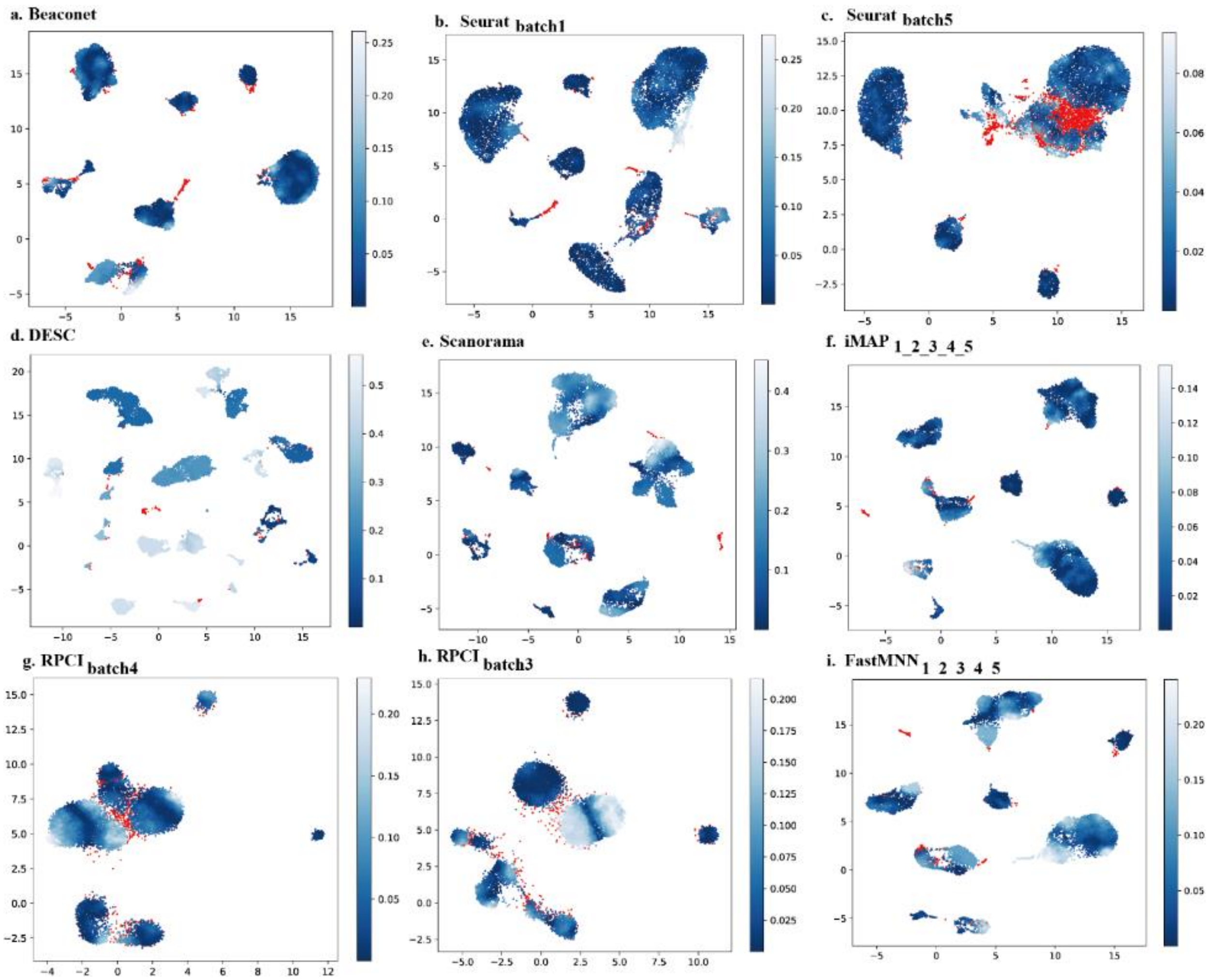

Fig S7. The PMD metric for intuitive observing the single cell-level batch effect on the results of five human pancreatic datasets. **a.** Beaconet. **b.** Seurat with reference batch 1. **c.** Seurat with reference batch 5. **d.** Scanorama. **e.** Scanorama **f.** iMAP with ordering “1_2_3_4_5”. **g.** RPCI with reference batch 4. **h.** RPCI with reference batch 3. **i.** FastMNN with ordering “1_2_3_4_5”.

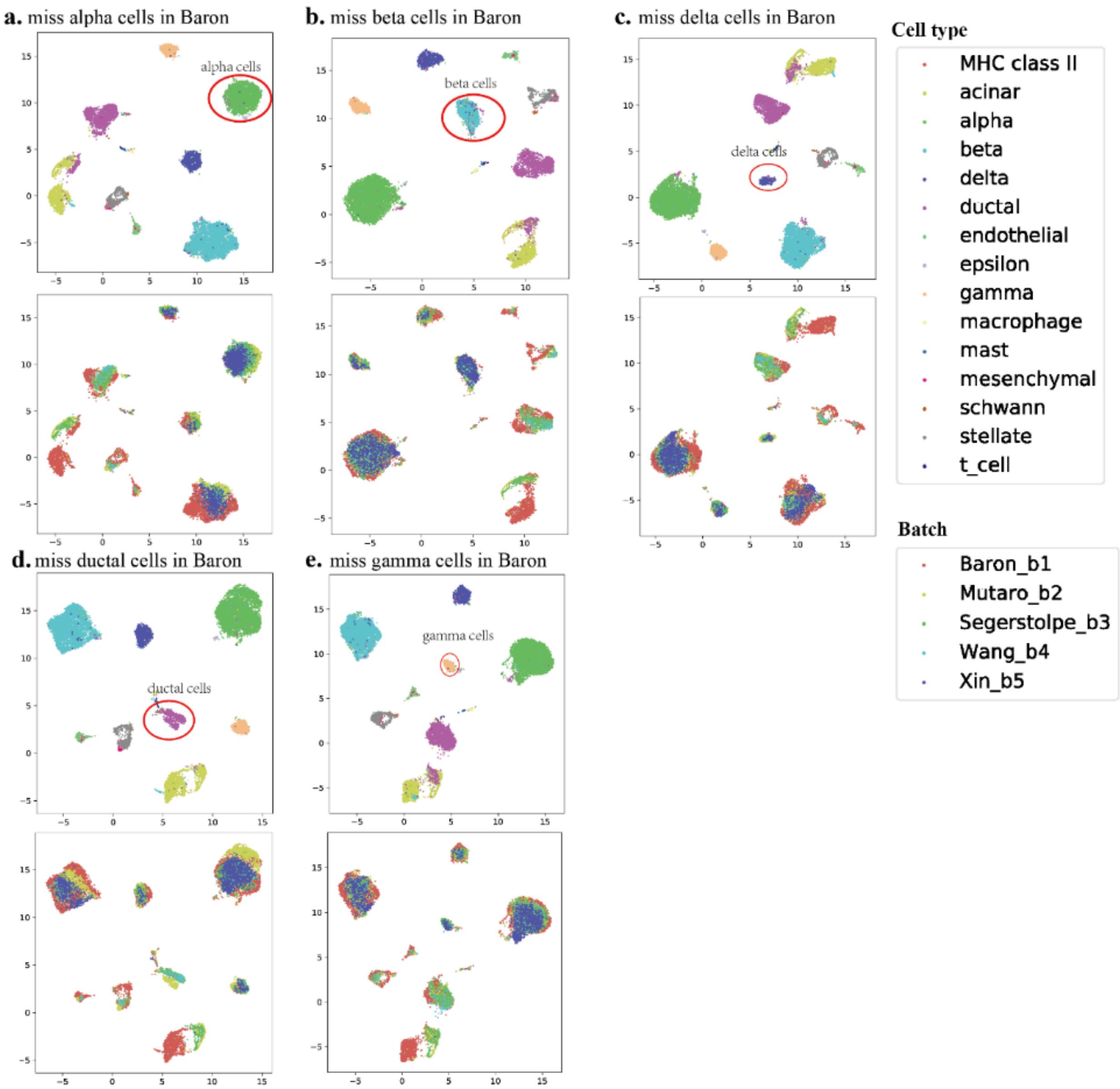

Fig S8. UMAP projection of five integrated human pancreatic datasets with artificial missing cell types using Beaconet. **a.** Miss alpha cells in “Baron” batch. **b.** Miss beta cells in “Baron” batch. **c.** Miss delta cells in “Baron” batch. **d.** Miss ductal cells in “Baron” batch. **e.** Miss gamma cells in “Baron” batch.

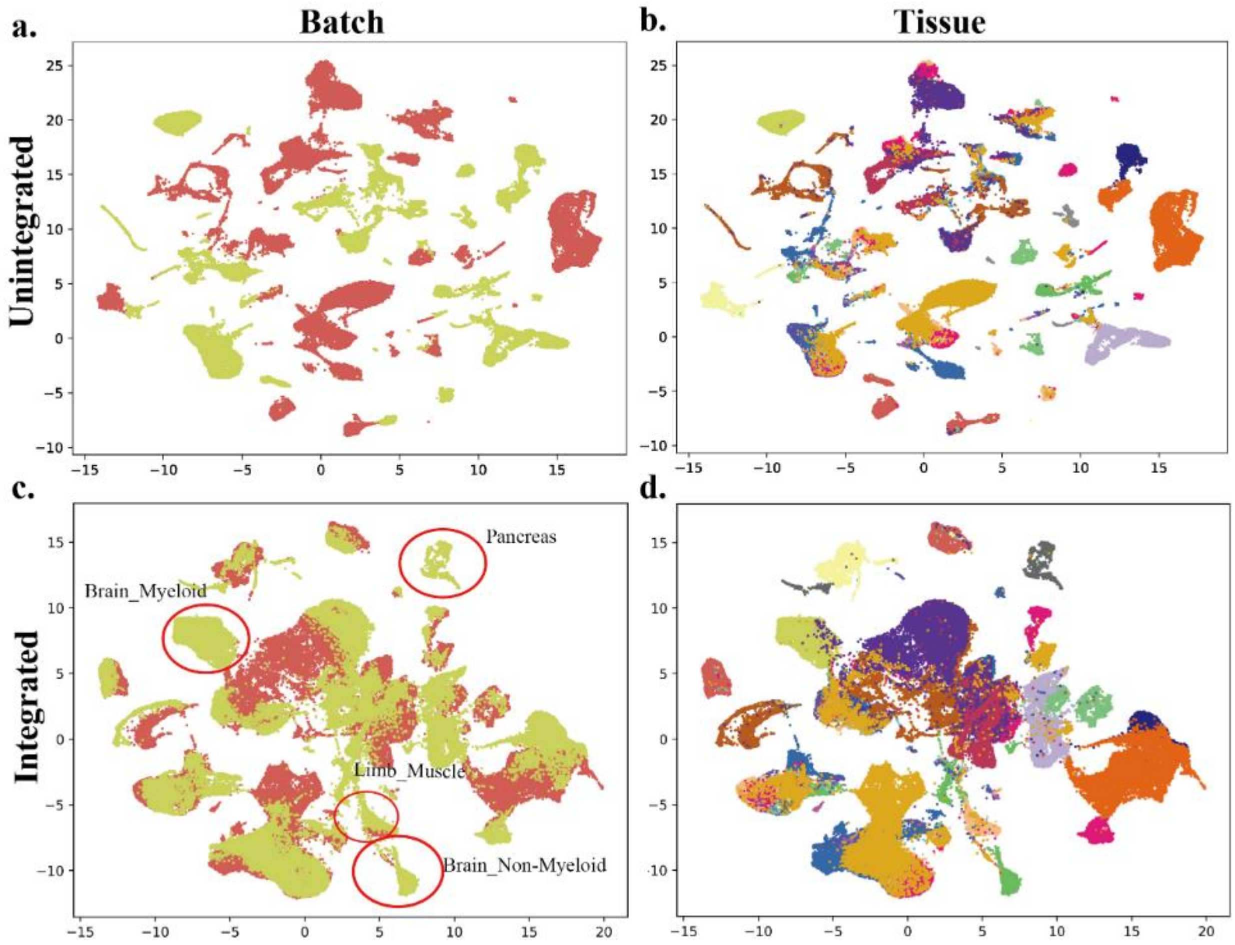

Fig S9. The UMAP projection of integrated and unintegrated Tabula muris datasets. **a.** The unintegrated two batches of Tabula muris datasets, colored by batches. **b.** The unintegrated two batches of Tabula muris datasets, colored by tissues. **c.** The integrated data of Tabula muris datasets using Beaconet, colored by batches. **d.** The integrated data of Tabula muris datasets using Beaconet, colored by tissues.

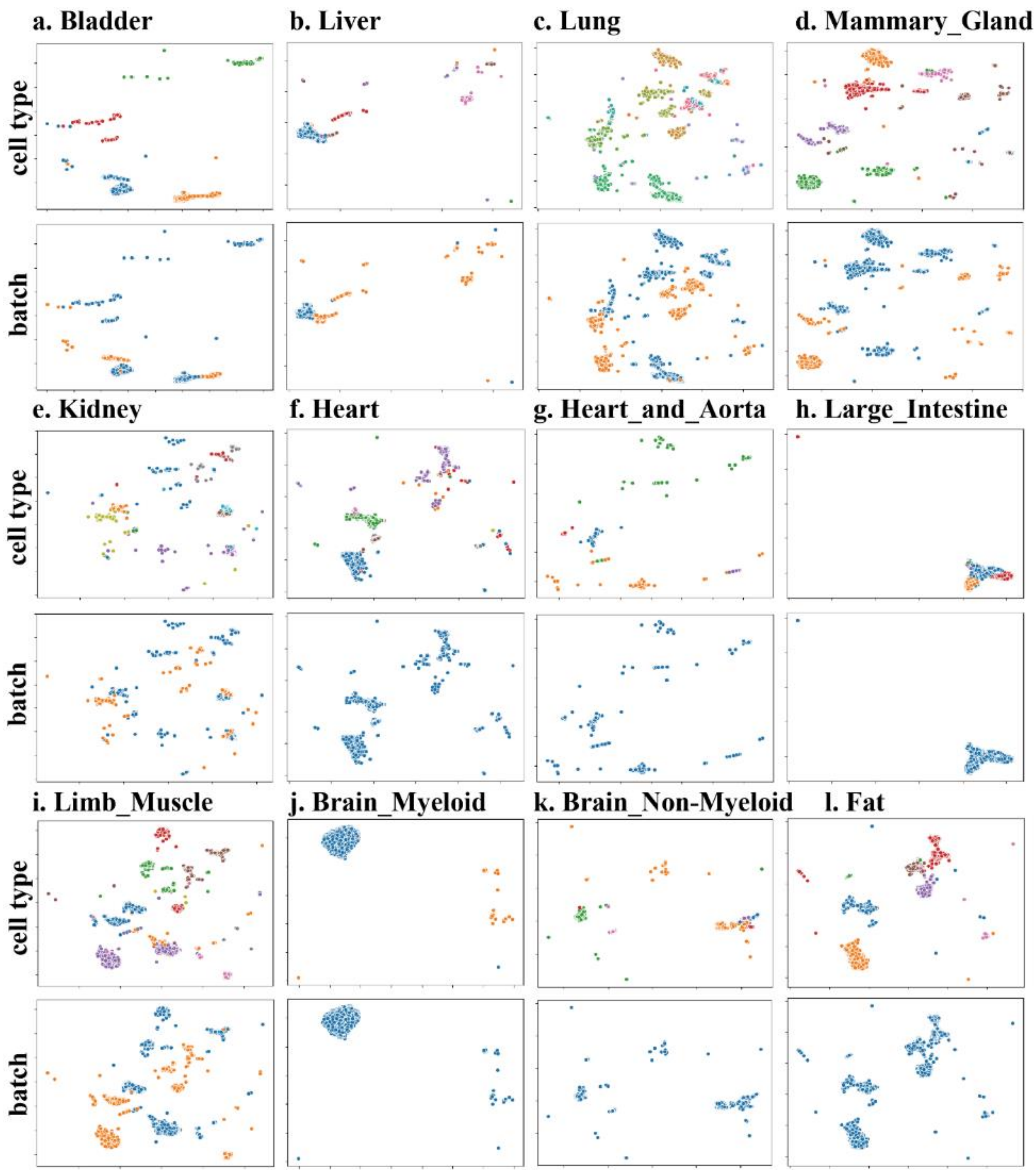

Fig S10. The visualization of unintegrated data of twelve tissues. **a.** Bladder. **b.** Liver. **c.** Lung. **d.** Mammary Gland. **e.** Kidney. **f.** Heart. **g.** Heart and Aorta. **h.** Large_intestine. **i.** Limb_muscle. **j.** Brain_Myeloid. **k.** Brain Non-Myeloid. **l.** Fat.

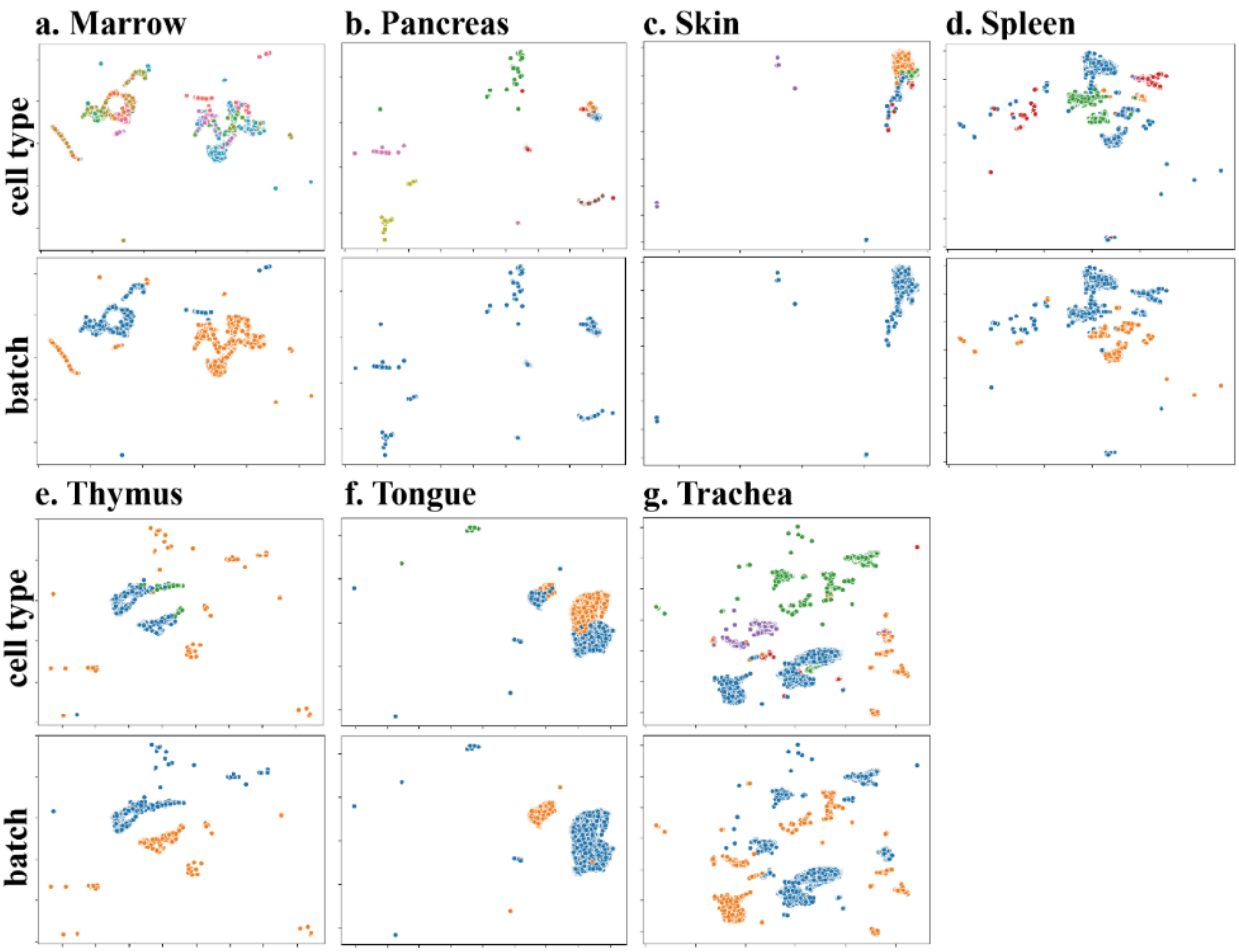

Fig S11. The visualization of unintegrated data of seven tissues. **a.** Marrow. **b.** Pancreas. **c.** Skin. **d.** Spleen. **e.** Thymus. **f.** Tongue. **g.** Trachea.

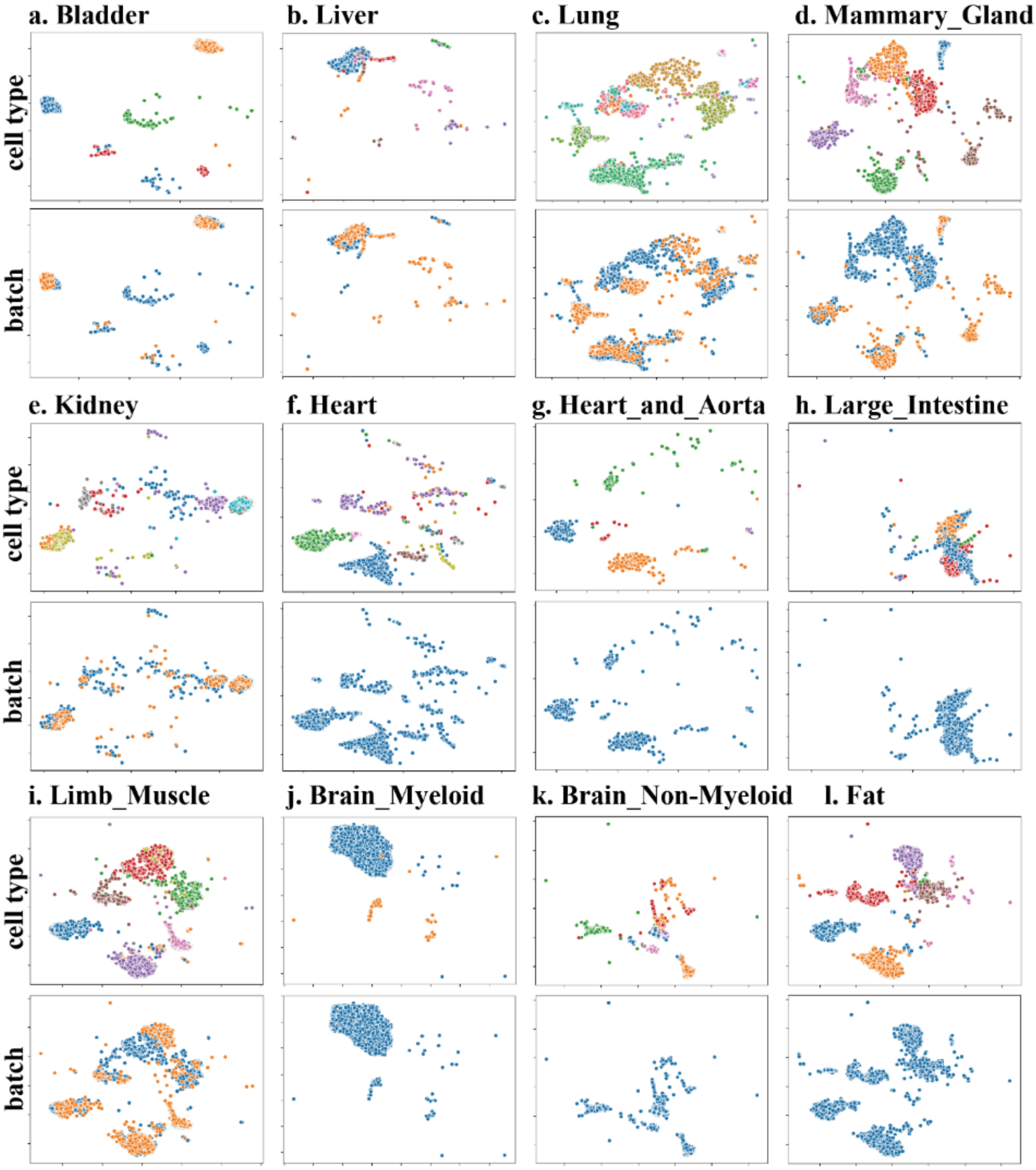

Fig S12. UMAP projection of twelve two-batch tissues integrated using Beaconet. **a.** Bladder. **b.** Liver. **c.** Lung. **d.** Mammary Gland. **e.** Kidney. **f.** Heart. **g.** Heart and Aorta. **h.** Large_intestine. **i.** Limb_muscle. **j.** Brain_Myeloid. **k.** Brain Non-Myeloid. **l.** Fat.

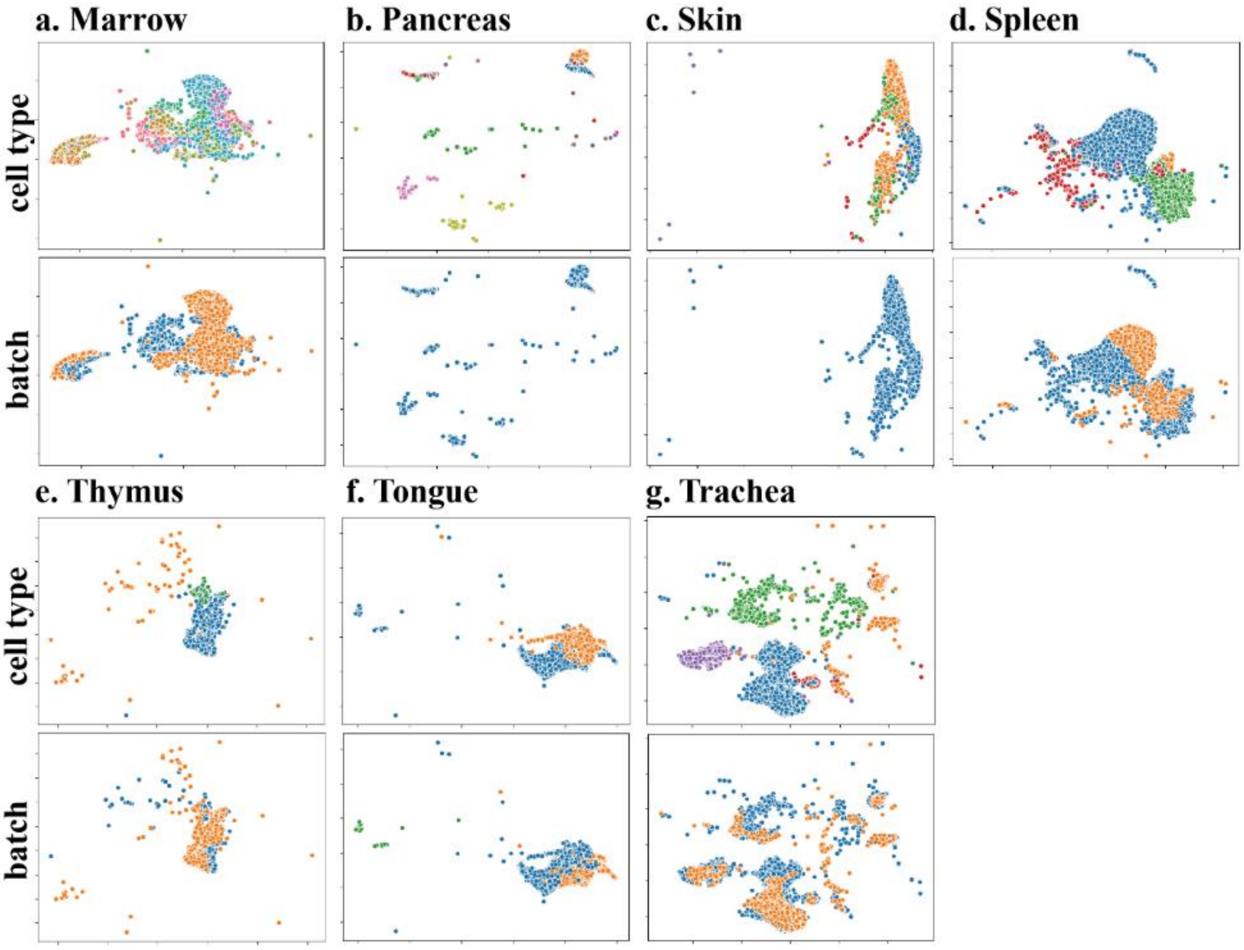

Fig S13. UMAP projection of seven two-batch tissues integrated using Beaconet. **a.** Marrow. **b.** Pancreas. **c.** Skin. **d.** Spleen. **e.** Thymus. **f.** Tongue. **g.** Trachea.

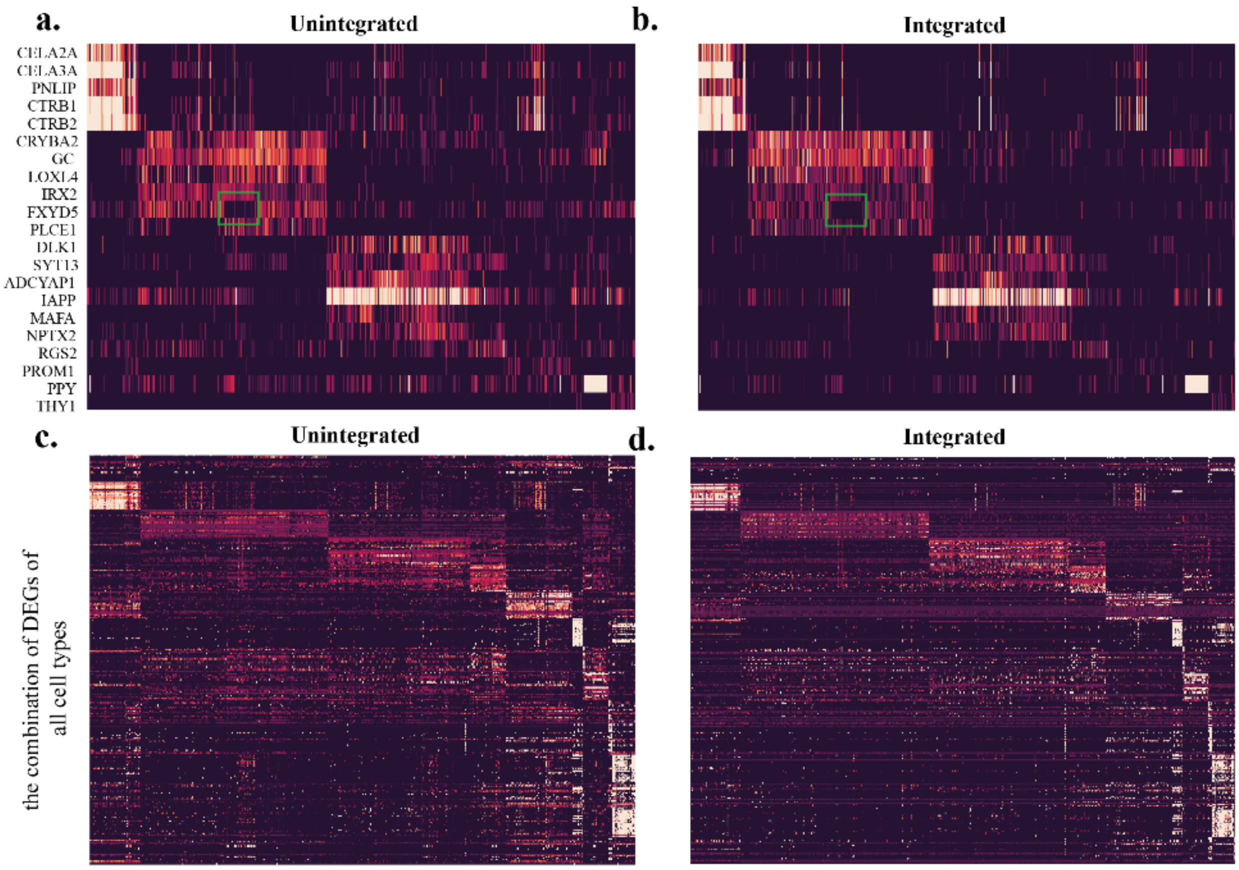

Fig S14. The effectiveness of the feature space of Beaconet. **a.** The expression pattern of the marker genes for major cell types in original data. **b.** The expression pattern of the marker genes for major cell types in the integrated data of Beaconet. **c.** The expression pattern of the differentially expressed genes for all cell types in original data. **d.** The expression pattern of the differentially expressed genes for all cell types in integrated data of Beaconet.

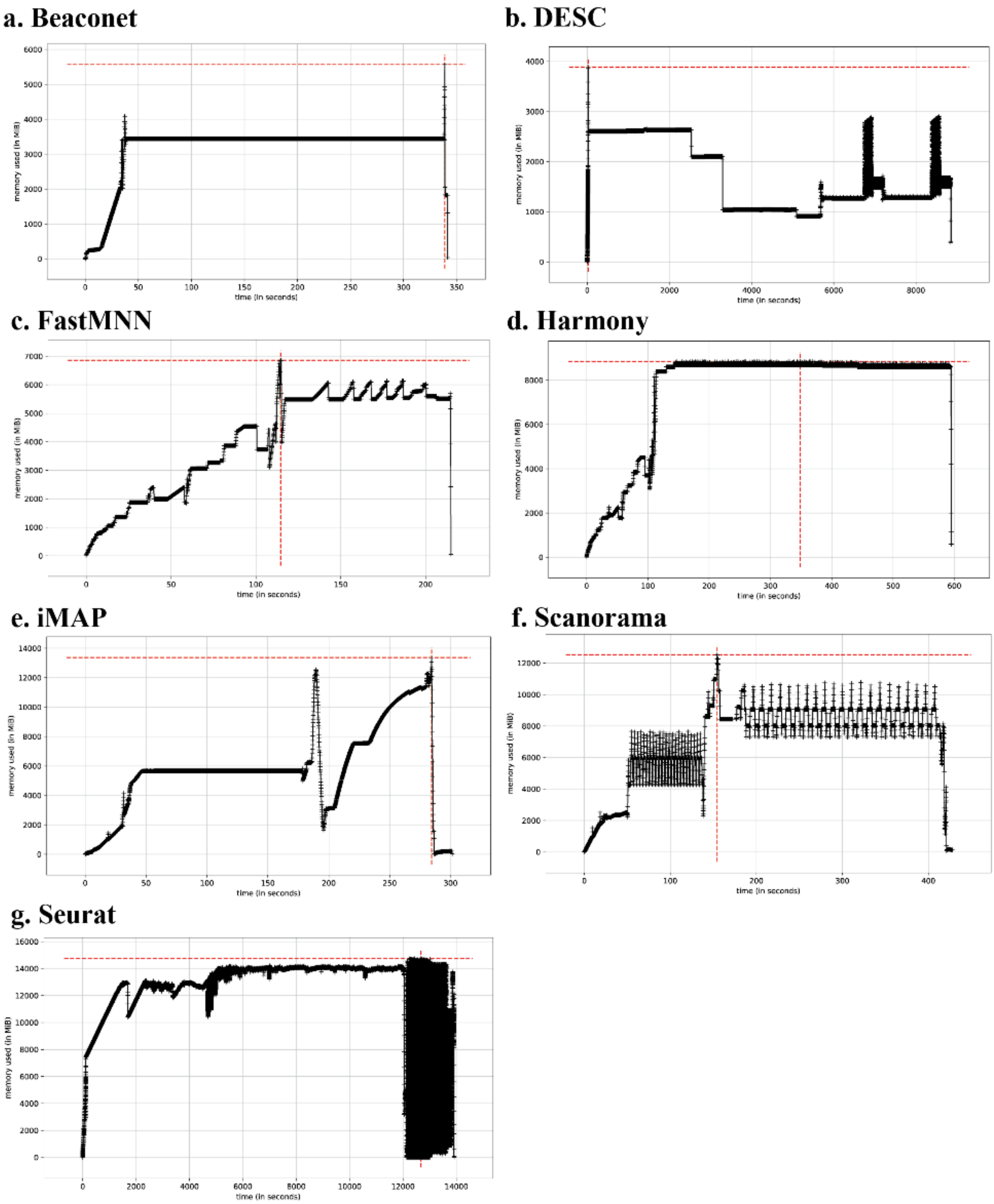

Fig S15. The fluctuation of the memory usage of the batch-effect removal methods. **a.** The expression pattern of the marker genes for major cell types in original data. **b.** The expression pattern of the marker genes for major cell types in the integrated data of Beaconet. **c.** The expression pattern of the differentially expressed genes for all cell types in original data. **d.** The expression pattern of the differentially expressed genes for all cell types in integrated data of Beaconet.

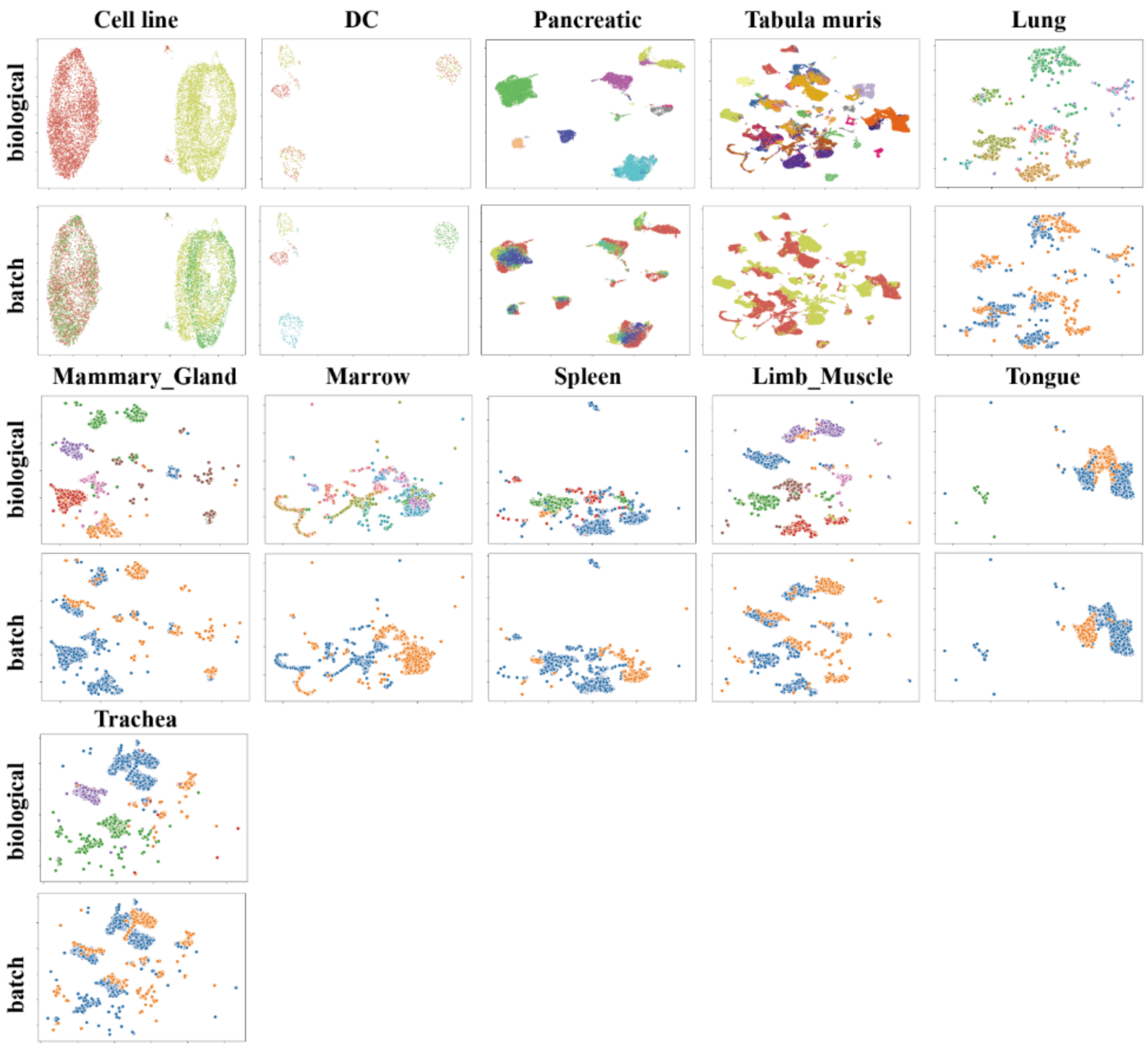

Fig S16. UMAP projection of the integrated data of the variant Beaconet on several datasets, including the three-batch cell line datasets, two-batch DC datasets, five-batch human pancreatic datasets, the seven tissues of mouse, including Lung, Mammary_gland, Marrow, Spleen, Limb_Muscle, Tongue and Trachea, on cell-type level and Tabula muris on tissue-level.

Note: the visualization of the full Beaconet on these datasets have provided in **Fig. 4a** and **Fig. S1, S3, S12, S13**.

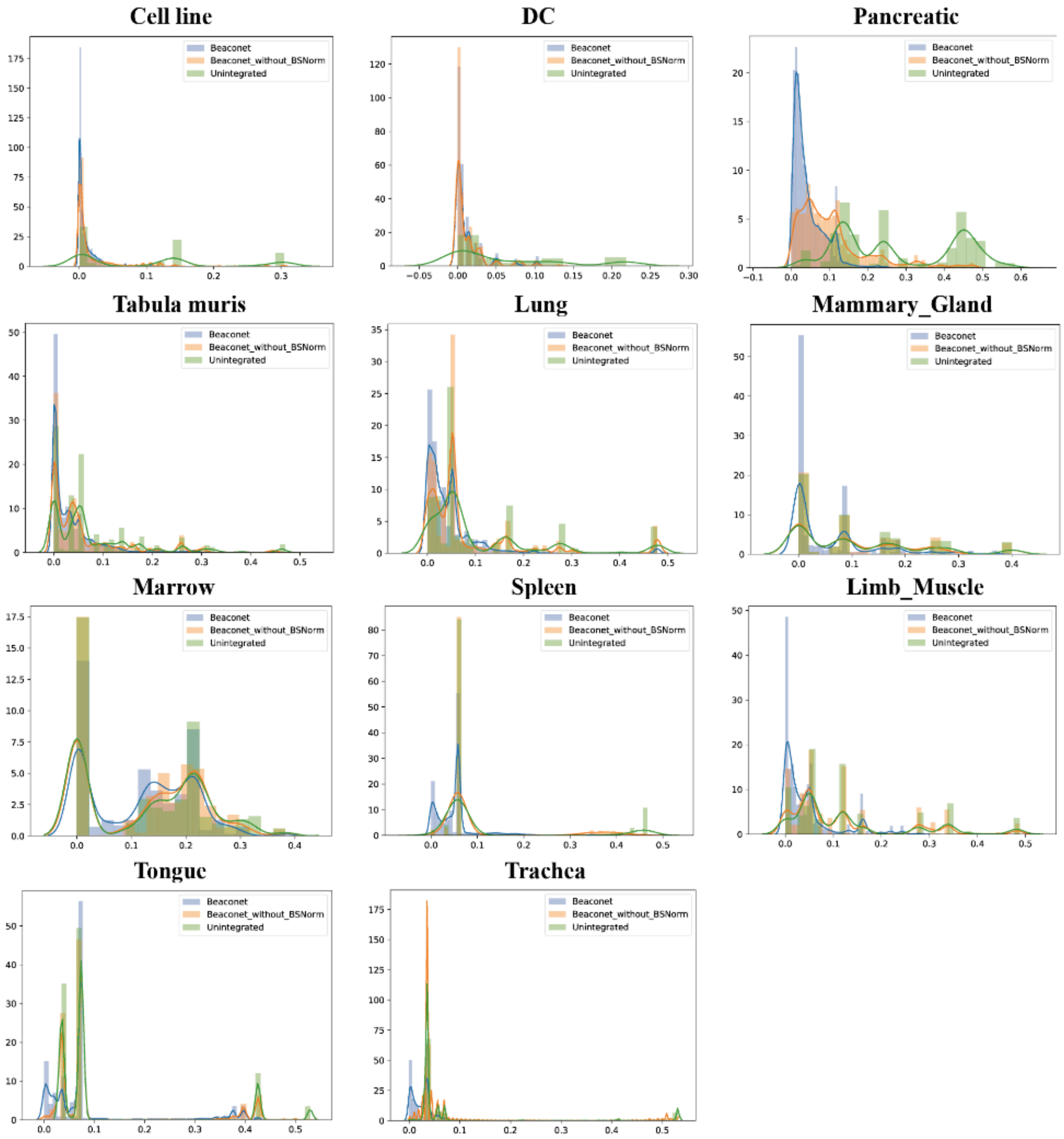

Fig S17. Comparison of the distribution of merge divergence for the full Beaconet and the variant method that disable the BS-Norm module.

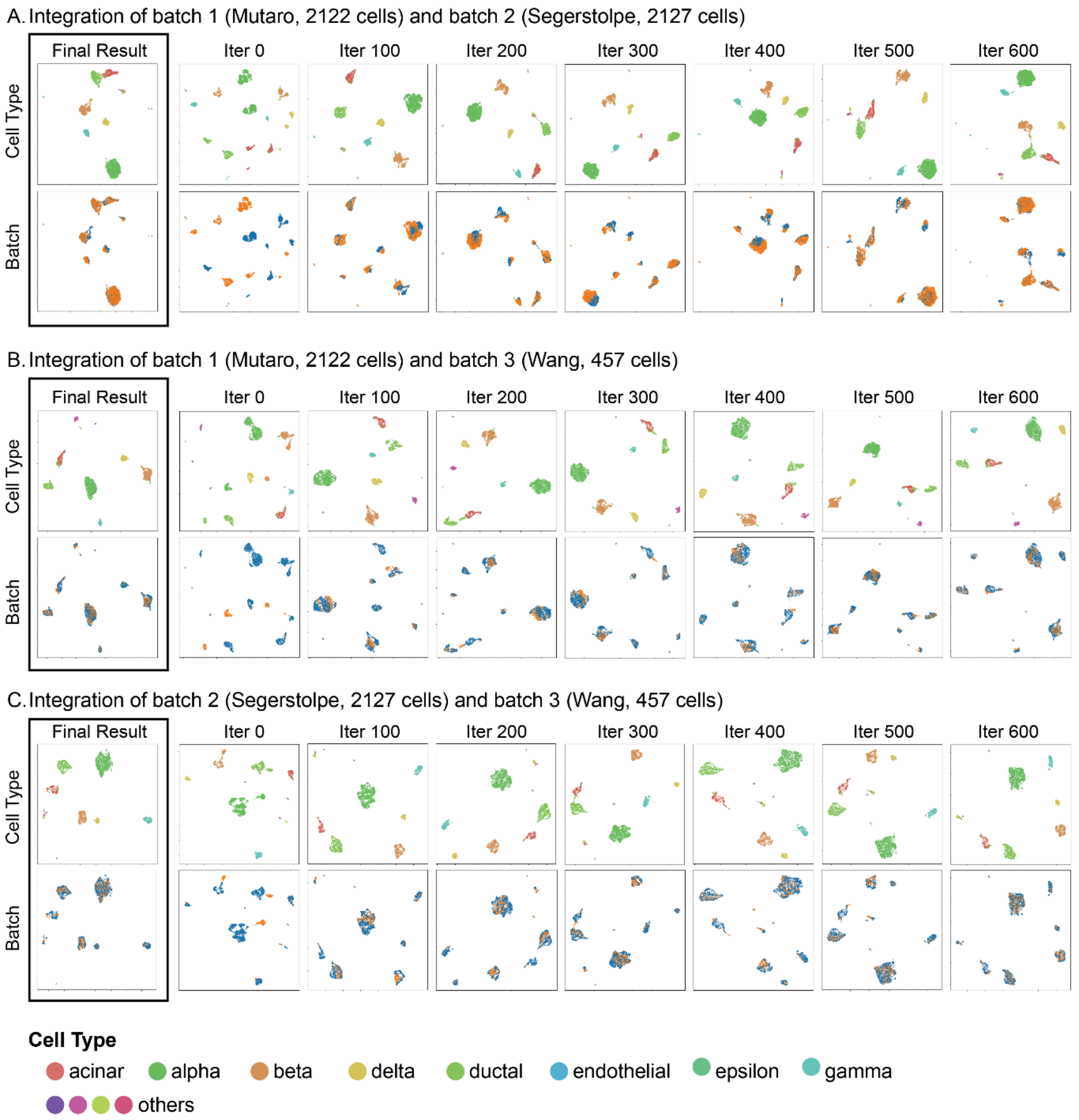

Fig S18. Visualization of the integrated data during training process using human pancreatic datasets. **a.** Integration of “Mutaro” batch and “Segerstolpe” batch. **b.** Integration of “Mutaro” batch and “Wang” batch. **c.** Integration of “Segerstolpe” batch and “Wang” batch.

The experiment in **Fig. S18** is to showcase the behavior of Beaconet during training process in order to aid in understanding Beaconet. We selected three batches human pancreatic datasets, including “Mutaro” [1], “Segerstolpe” [2], and “Wang” [3]. The number of cells in “Mutaro” and “Segerstolpe” are roughly balance (2122 cells and 2127 cells). The number of cells in batch

“Wang” (457 cells) is much smaller than the other two datasets. We visualized the intermediate results of every 100 iteration during training for three tasks (integrating two balanced batches “Mutaro” and “Segerstolpe”; integrating unbalanced batches “Mutaro” and “Wang”; integrating two unbalanced batches “Segerstolpe” and “Wang”). As shown in Fig. S18, in the first 100 iteration of training process, the centers of the same cell populations in different batches were roughly merged, and then, the clusters are mixed better gradually from the 200th to 600th iteration. The final integrated results shown in the first column show that the different batches of datasets are well-integrated. This result is a case for exhibiting that Beaconet gradually learns to reduce the batch difference between datasets in the training process.

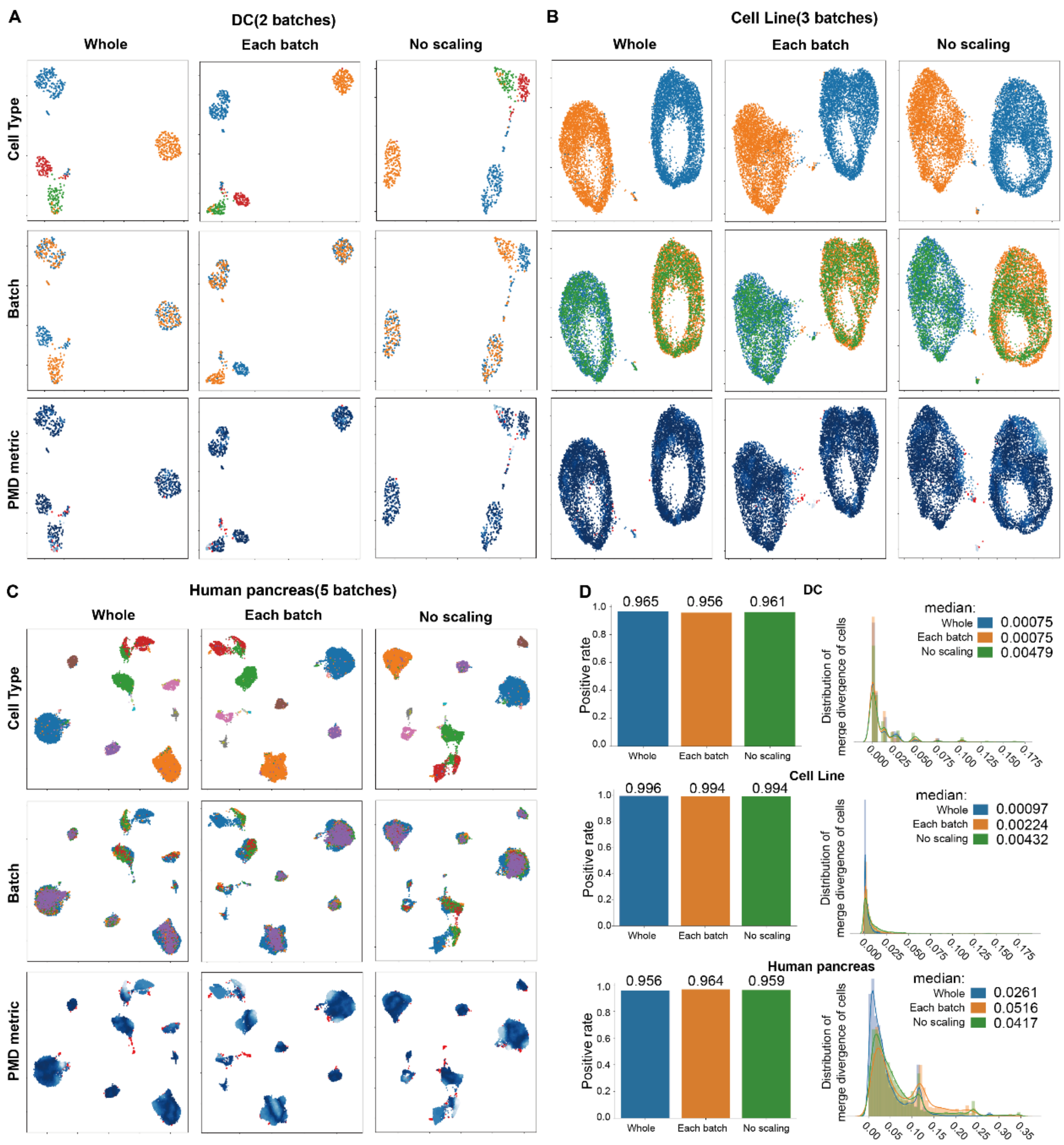

Figure S19. Beaconet is robust to the three scaling methods. The performance of Beaconet on three integration tasks with "scaling on the whole dataset", "scaling on each batch" and "no scaling" input data. **a.** UMAP visualization of integrated DC datasets in three scaling methods. The scatterplots were colored by cell type, batch label, PMD scores respectively. **b.** UMAP visualization of integrated Cell Line datasets in three scaling methods. **c.** UMAP visualization of integrated Human pancreas datasets in three scaling methods. **d.** Comparison of performance of Beaconet on three scaling methods using three integration tasks.

We have tested the integration capacity of Beaconet for different scaling methods by nine cases, which are combination of three scaling methods ("scaling on each batch", "scaling on the whole dataset" and

“no scaling at all”) and three integration tasks (two-batch DC data, three-batch cell line data, five-batch human pancreas data). For investigating the performance of Beaconet in practice, we did not fine-tune any hyper-parameters for different scaling methods and integration tasks.

Referring to the scaling strategies of FIRM [16] and LIGER [17], the genes were divided by standard deviation to scale to unit-variance but not centered to zero-mean by subtracting the mean for “scaling on each batch” and “scaling on the whole dataset”. It is because **Beaconet is originally designed for log-scaled expression data** and the non-positive input is not applicable (More details for the network architecture of Beaconet is available at Section 5.2). For “scaling on each batch”, the genes with zero-expression on individual batch were filtered before scaling. For scaling on the whole dataset and no scaling, genes with zero-expression for all cells on the whole dataset were filtered.

The results are summarized as **Fig. S19**. Beaconet successfully integrated the datasets for eight cases in the nine combinations of three integration tasks and three scaling methods. On DC data integration (**Fig. S19A**), Beaconet consistently distinguished the batch-specific cell types (red and green) with three scaling methods. On the five batches of human pancreatic data integration (**Fig. S19C**), the batch variations were removed and the purity of major cell types were preserved. On cell line data integration (**Fig. S19B**), Beaconet properly integrated the two monoclonal cell types in three batches for two scaling methods (“scaling on the whole dataset” and “no scaling”). In the result for “scaling on each batch” on cell line dataset, although Beaconet preserved the purity of cell groups, it did not merge the cells from different batches. It may result from that both of batch 1 and batch 2 have only a single cell type (293T cell and Jurkat cell respectively). When applying “scaling on each batch” for such datasets, the batch-specific system bias among batch were enhanced, and lead to harder matching of cells among batches in unsupervised integration manner. The positive rates and distribution of merge divergence scores of cells (**Fig. S19D**) also indicated that Beaconet preserved the purity of cell types in the nine integration results, and successfully removed batch variant in eight integration results.

Although Beaconet could work for most cases (eight of the nine cases) in **Fig. S19A-C**, we suggest to not scale on each batch before applying integration method, especially for datasets may have fewer shared cell populations among batches. It is because that scaling on each batch is a batch-wise operation that

handle and transform the data in different batches separately. It changes the relative distance of cells among batches and may lead to unnecessary complex batch bias. In the traditional scRNA-seq processing pipeline for downstream analysis, once we decided to integrate datasets from multiple experiments, it is a natural choice to apply an integration method to reduce batch effect, rather than applying some batch-wise processing for handling each batch separately. It is noted that the fundamental preprocessing such as log1p-transformation and normalization for total counts of cells are element-wise or cell-wise transformation. These operations handle each value or cell independently, and thus the processed results did not affect by other cells in the batch or dataset. “Scaling on the whole dataset” only changes the scaling ratio of the whole dataset, it does not change the relative distance of cells among batches.

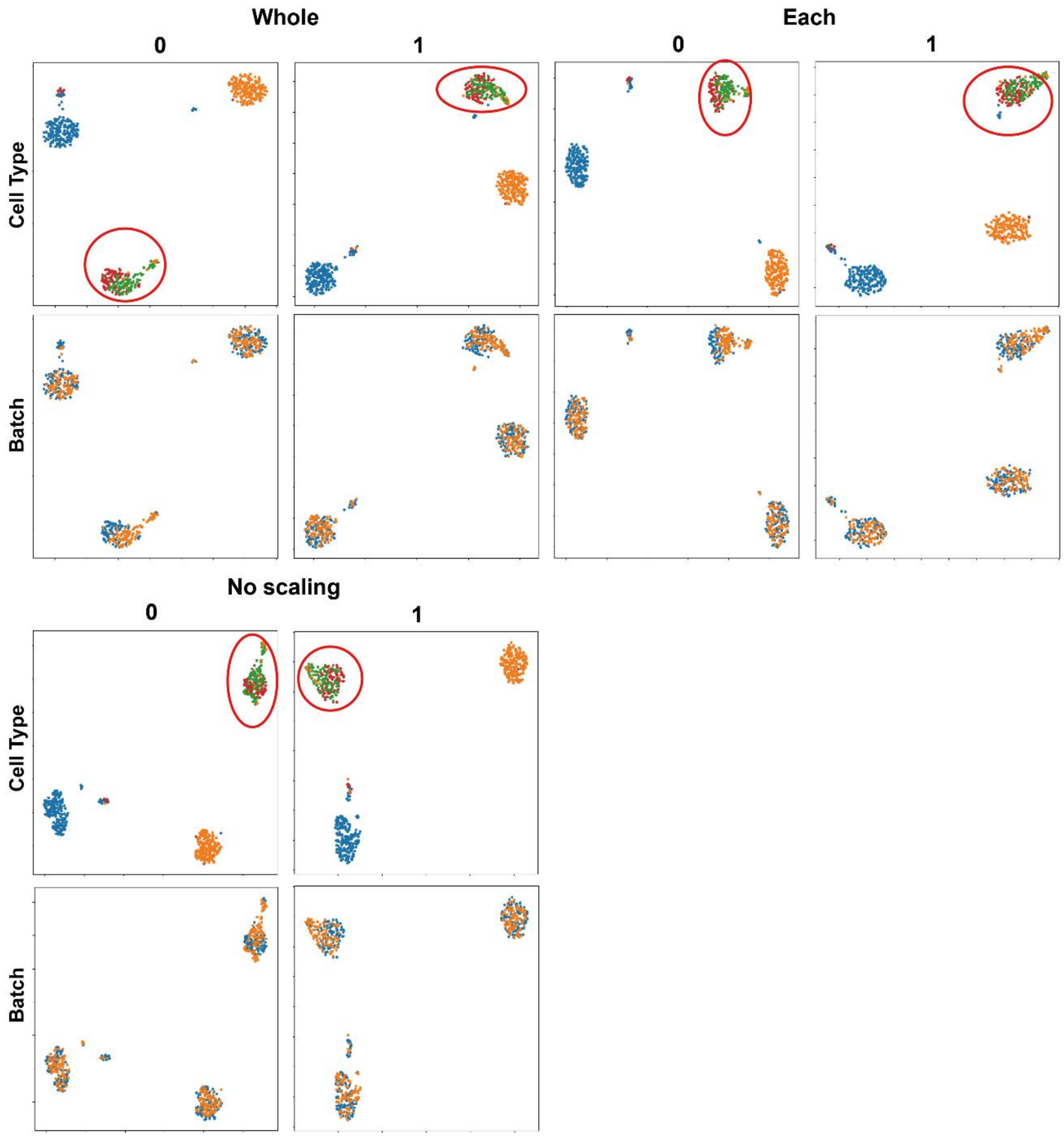

Fig. S20. Seurat integrates two-batch DC data with scaling on the whole dataset, scaling on each dataset, and no scaling.

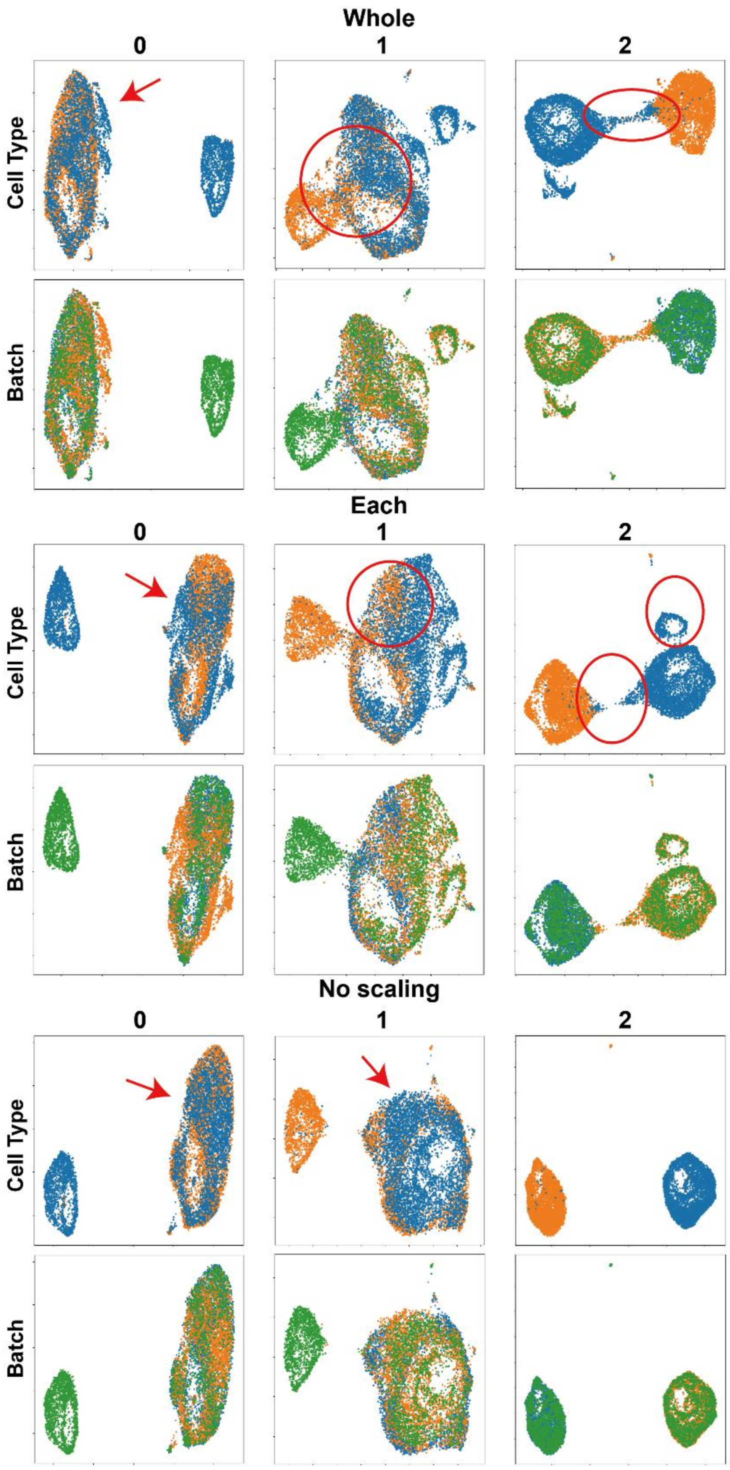

Fig. S21. Seurat integrates three-batch cell line data with scaling on the whole dataset, scaling on each dataset, and no scaling.

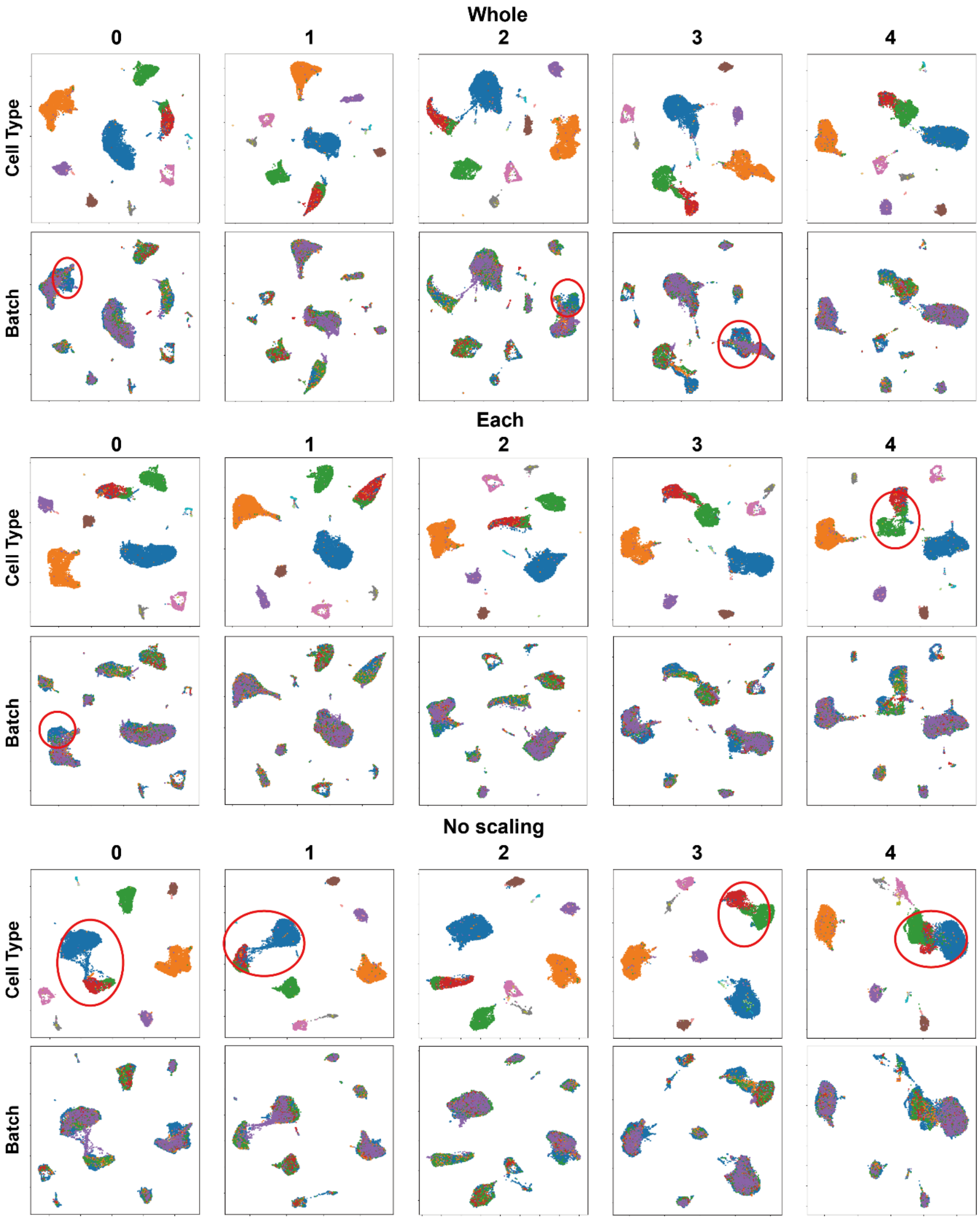

Fig. S22. Seurat integrates five-batch human pancreas data with scaling on the whole dataset, scaling on each dataset, and no scaling.

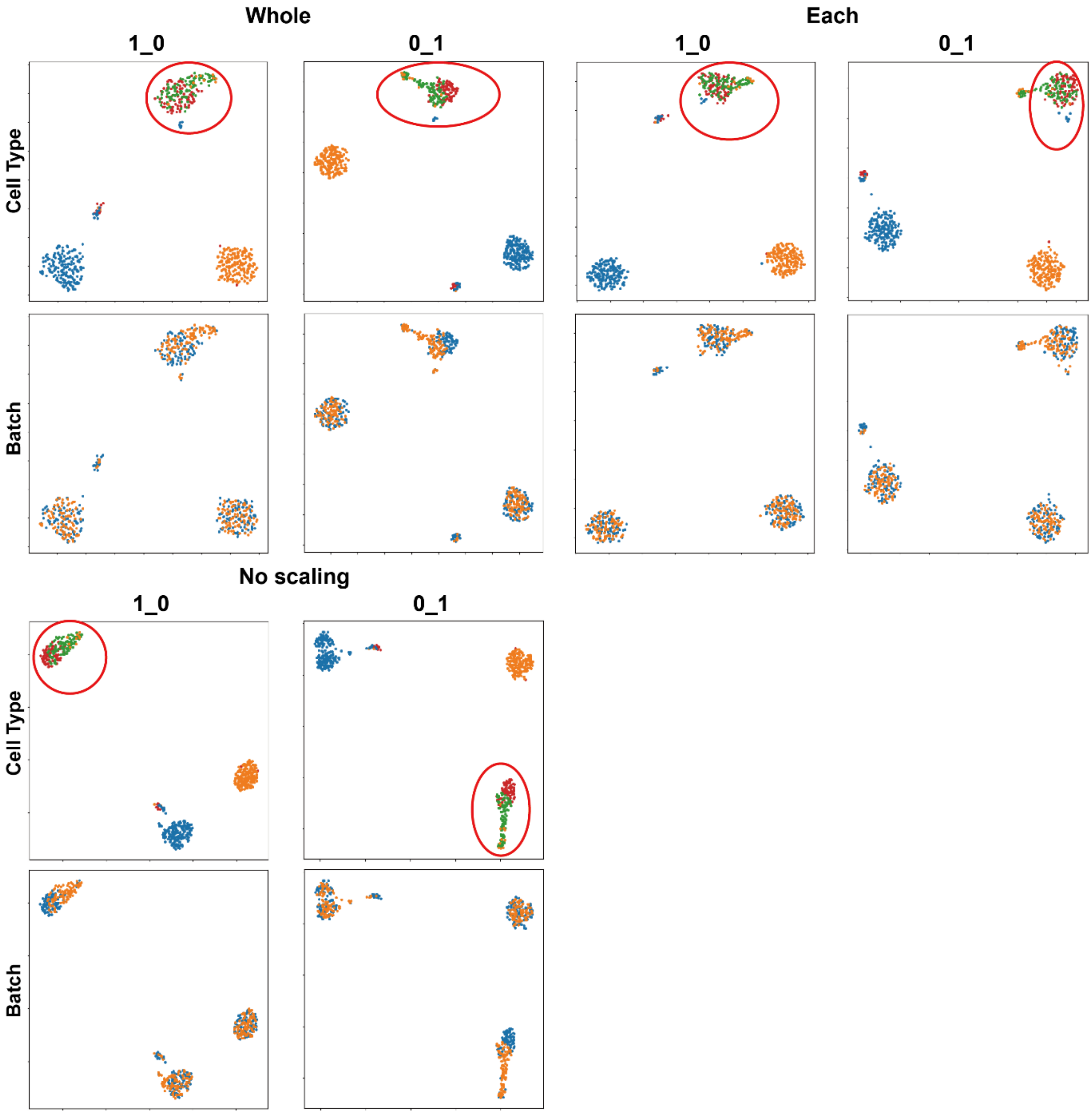

Fig. S23. FastMNN integrates two-batch DC data with scaling on the whole dataset, scaling on each dataset, and no scaling.

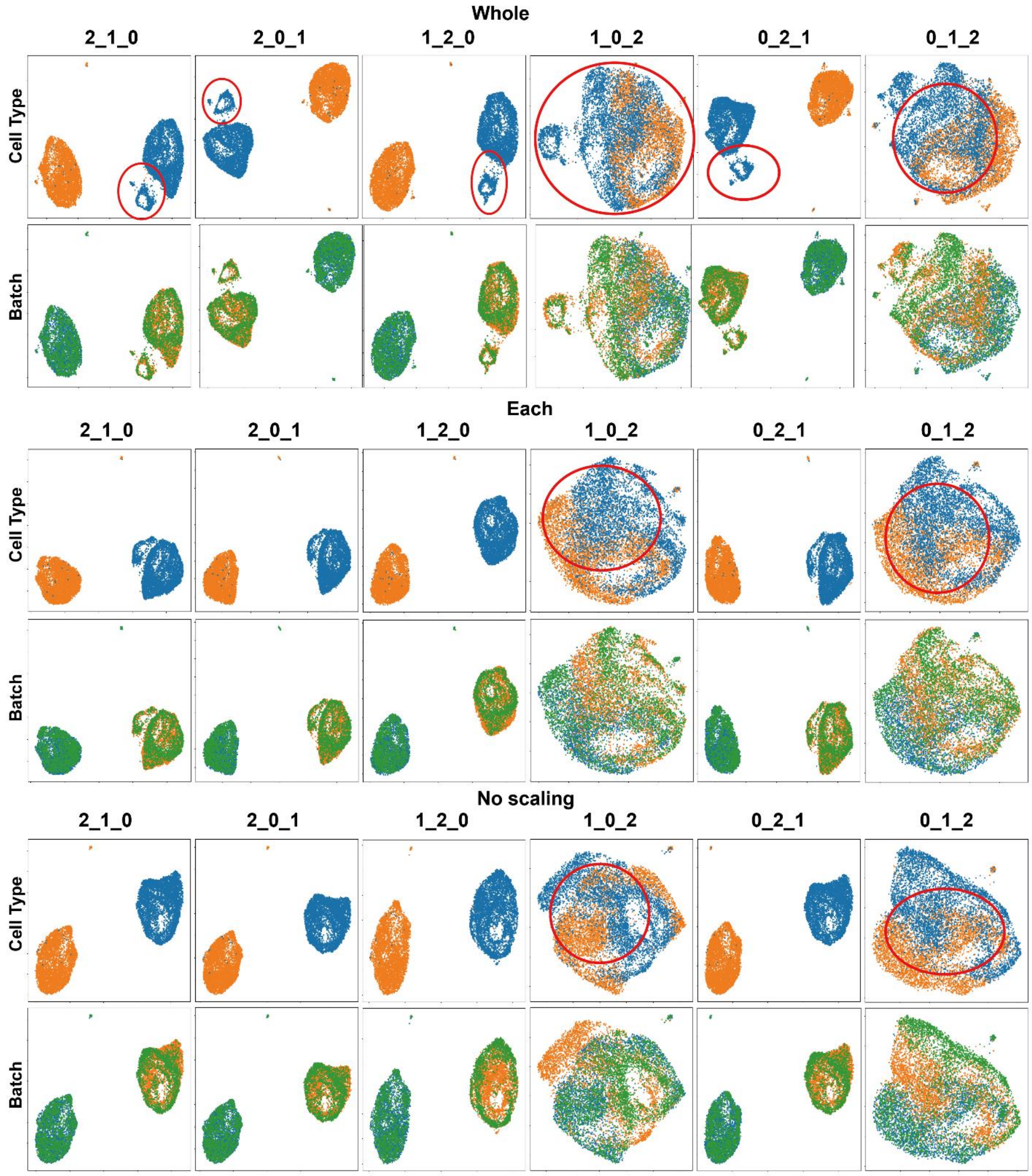

Fig. S24. FastMNN integrates three-batch cell line data with scaling on the whole dataset, scaling on each dataset, and no scaling.

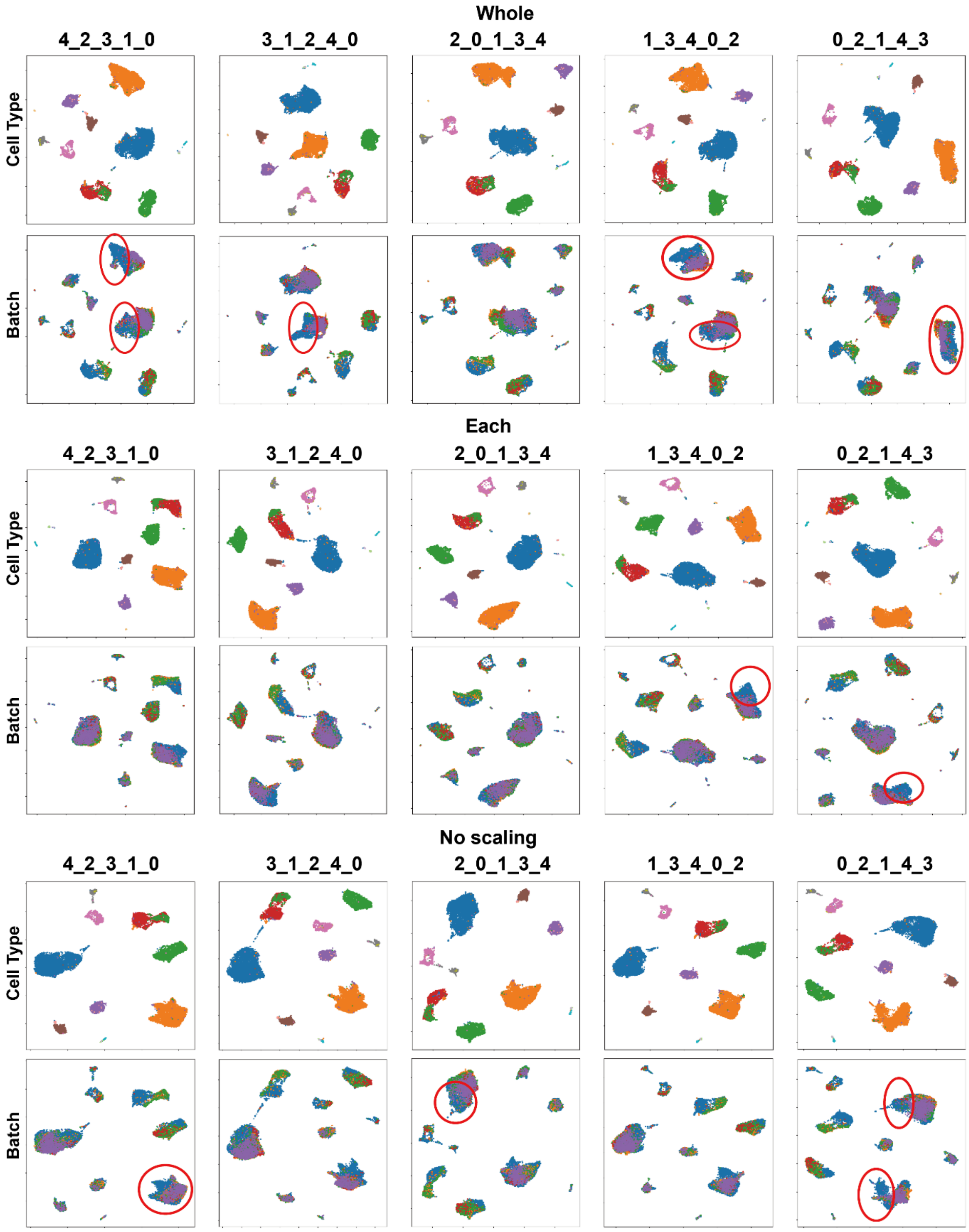

Fig. S25. FastMNN integrates five-batch human pancreas data with scaling on the whole dataset, scaling on each dataset, and no scaling. We randomly select five merge orders from the 120 possible orders by numpy.random.choice() function with random seed 0 for this integration task.

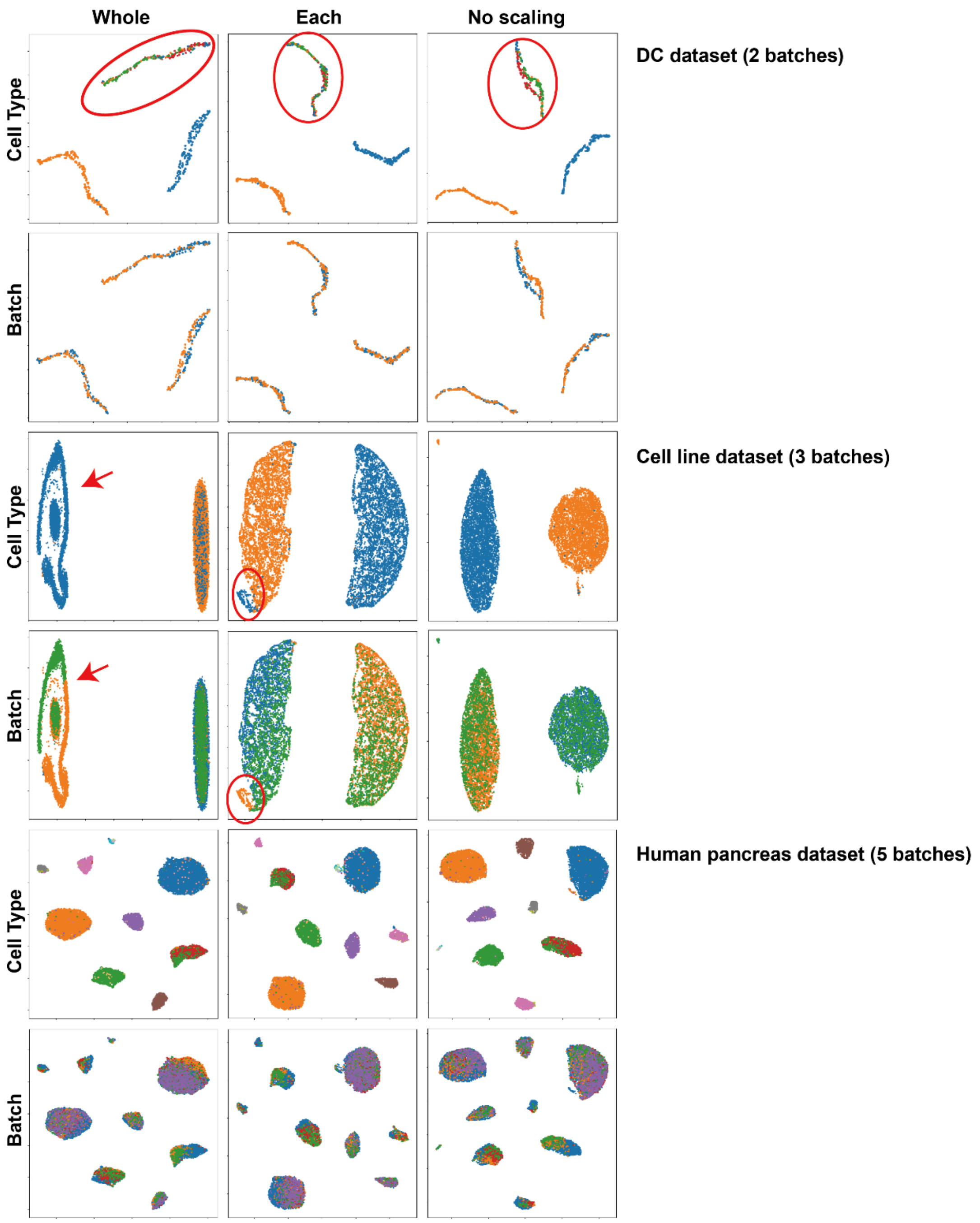

Fig. S26. scDML integrates two-batch DC data, three-batch cell line data, and five-batch human pancreas data with scaling on the whole dataset, scaling on each dataset, and no scaling.

Reference

1. Arjovsky M, Chintala S, Bottou L. Wasserstein generative adversarial networks. Int Conf Mach Learn. PMLR; 2017. p. 214–23.

2. Gulrajani I, Ahmed F, Arjovsky M, Dumoulin V, Courville AC. Improved training of wasserstein gans. Adv Neural Inf Process Syst. 2017;30.

3. Kingma DP, Ba J. Adam: A method for stochastic optimization. ArXiv Prepr ArXiv14126980. 2014;

4. Han Xu, Lin Gao. The implementation of Beaconet using Python. [Internet]. [cited 2022 Sep 10]. Available from: https://github.com/GaoLabXDU/Beaconet

5. Wang D, Hou S, Zhang L, Wang X, Liu B, Zhang Z. iMAP: integration of multiple single-cell datasets by adversarial paired transfer networks. Genome Biol. 2021;22:63.

6. Haghverdi L, Lun ATL, Morgan MD, Marioni JC. Batch effects in single-cell RNA-sequencing data are corrected by matching mutual nearest neighbors. Nat Biotechnol. 2018;36:421–7.

7. Wang CX, Zhang L, Wang B. One Cell At a Time (OCAT): a unified framework to integrate and analyze single-cell RNA-seq data. Genome Biol. 2022;23:102.

8. Büttner M, Miao Z, Wolf FA, Teichmann SA, Theis FJ. A test metric for assessing single-cell RNA-seq batch correction. Nat Methods. 2019;16:43–9.

9. Korsunsky I, Millard N, Fan J, Slowikowski K, Zhang F, Wei K, et al. Fast, sensitive and accurate integration of single-cell data with Harmony. Nat Methods. 2019;16:1289–96.

10. Tran HTN, Ang KS, Chevrier M, Zhang X, Lee NYS, Goh M, et al. A benchmark of batch-effect correction methods for single-cell RNA sequencing data. Genome Biol. 2020;21:12.

11. Liu Y, Wang T, Zhou B, Zheng D. Robust integration of multiple single-cell RNA sequencing datasets using a single reference space. Nat Biotechnol [Internet]. 2021; Available from: https://doi.org/10.1038/s41587-021-00859-x

12. Steinley D. Properties of the hubert-arable adjusted rand index. Psychol Methods. 2004;9:386.

13. Luecken MD, Büttner M, Chaichoompu K, Danese A, Interlandi M, Mueller MF, et al. Benchmarking atlas-level data integration in single-cell genomics. Nat Methods. 2022;19:41–50.

14. Zhao J, Wang G, Ming J, Lin Z, Wang Y, Agarwal S, et al. Adversarial domain translation networks for integrating large-scale atlas-level single-cell datasets. Nat Comput Sci. 2022;2:317–30.

15. Vinh NX, Epps J, Bailey J. Information theoretic measures for clusterings comparison: is a correction for chance necessary? Proc 26th Annu Int Conf Mach Learn. 2009. p. 1073–80.

16. Ming J, Lin Z, Zhao J, Wan X, The Tabula Microcebus Consortium, Yang C, et al. FIRM: Flexible integration of single-cell RNA-sequencing data for large-scale multi-tissue cell atlas datasets. Brief Bioinform. 2022;23:bbac167.

17. Welch JD, Kozareva V, Ferreira A, Vanderburg C, Martin C, Macosko EZ. Single-cell multi-omic integration compares and contrasts features of brain cell identity. Cell. 2019;177:1873–87.
